# Role of Nuclear SUMO-Targeted Ubiquitin Ligase Uls2 (Slx5-Slx8) in Quality Control of Cytosolic Proteins

**DOI:** 10.3390/biom16091322

**Published:** 2026-09-11

**Authors:** Friederike Profe-Austermann, Lennard-Maximilian Döring, Luisa Schmidt, Lena Avci, Maximilian Haka, Ganapathi Kandasamy, Judith Müller, Nils Johnsson, Marcus Krüger, R. Jürgen Dohmen

**Affiliations:** 1 Center of Molecular Biosciences, Institute for Genetics, Department of Biology, Faculty of Natural Sciences and Mathematics, University of Cologne, 50674 Cologne, Germany; lennarddoering@gmx.de (L.-M.D.); lena.avci@yahoo.de (L.A.); mhaka1@uni-koeln.de (M.H.); gxk400@miami.edu (G.K.); 2 Institute for Genetics, Cologne Excellence Cluster on Cellular Stress Responses in Aging-Associated Diseases (CECAD), 50674 Cologne, Germany; luisa.schmidt@sund.ku.dk (L.S.); marcus.krueger@uni-koeln.de (M.K.); 3 Department of Biology, Institute of Molecular Genetics and Cell Biology, Ulm University, 89081 Ulm, Germanynils.johnsson@uni-ulm.de (N.J.); 4 Center for Molecular Medicine (CMMC), University of Cologne, 50674 Cologne, Germany

**Keywords:** SUMO, STUbL, quality control, Slx5, Slx8, Nis1, Fir1

## Abstract

The interplay between the posttranslational modifiers SUMO and ubiquitin mediates the degradation of proteins. This crosstalk is conveyed by SUMO-targeted ubiquitin ligases (STUbLs). The best-studied STUbL in *Saccharomyces cerevisiae* is the heterodimer Slx5-Slx8 (Uls2). Uls2 mainly resides in the nucleus and is involved in stress tolerance, DNA repair and genome stability maintenance. So far, the majority of known Uls2 targets are nuclear proteins. The role of Uls2 in the targeting of cytosolic proteins remains to be elucidated. In this study, we identify Nis1 and Fir1 as two cytosolic proteins that become substrates of Uls2 in the absence of their partner polypeptides, which results in their SUMO-dependent aggregate formation in the nucleus. To uncover further Uls2 targets, we performed whole-cell proteomics of *slx5Δ* cells with and without oxidative stress. Comparison to wild-type cells revealed a large pool of potential Uls2 substrates, a subset of which is targeted upon oxidative stress, suggesting a quality control (QC) mechanism. Based on our results, we propose a role for Uls2 in a QC system targeting cytosolic proteins lacking their partners or that are damaged, for example, by oxidation.

## 1. Introduction

Attachment of the small ubiquitin-related modifier (SUMO) can have a multitude of effects on its targets. SUMOylation is able to activate or inactivate proteins, foster interaction between proteins, influence their localization, or lead to their degradation [1,2,3,4,5,6,7]. SUMO-mediated degradation depends on its crosstalk with its cousin ubiquitin. This crosstalk is conveyed by SUMO-targeted ubiquitin ligases (STUbLs) [1,8]. STUbLs are defined by two motifs. They carry multiple SUMO-interacting motifs (SIMs) and a RING domain. SIMs are short peptide sequences consisting of a hydrophobic core adjacent to acidic residues. The hydrophobic core interacts with a hydrophobic groove on SUMO and thus fosters the interaction between the SIM-containing protein and SUMO [2,3]. The RING domain creates a binding platform for ubiquitin-conjugating (E2) enzymes, thereby conveying the ubiquitylation of target proteins. Via their SIMs, STUbLs recognize polySUMOylated proteins, and then recruit a ubiquitin-conjugating E2 enzyme with their RING domain. This leads to the ubiquitylation of the SUMO chain on the substrate, which causes degradation of the so-modified protein by the proteasome [8,9,10,11]. *S. cerevisiae* has three STUbL systems: Rad18 and the ubiquitin ligases for SUMO conjugates (Uls) Uls1 and Uls2 [9,12,13]. Rad18 was shown to have STUbL activity only toward the sliding clamp protein PCNA [12]. Aside from the characteristics of a STUbL (SIMs and RING), Uls1 has domains shared with SWI2/SNF2 family translocases, and its functions have been linked to DNA replication stress as well as responses to double-strand breaks [9,14,15]. Substrates of Uls1 STUbL activity include Srs2 and Kar9 [16,17]. Uls1 targeting of Kar9 is relevant mainly in the absence of Uls2, consistent with some functional overlap of Uls1 and Uls2 [9].

The best-studied STUbL in *S. cerevisiae* is Uls2, a heterodimer consisting of the subunits Slx5 (alias Hex3) and Slx8 [10,18]. Both subunits carry a RING domain; Slx8 has one SIM, whereas Slx5 has a total of four SIMs [9]. As a complex, Slx5 and Slx8 (Uls2) reside in the nucleus, and Slx5 has been reported to localize to nuclear foci as well as at DNA double-strand breaks [19]. Uls2 is involved in a wide variety of cellular processes, such as maintenance of genome stability, which has been extensively studied [8,18,20,21,22]. Furthermore, Uls2 is involved in stress tolerance together with the segregase Cdc48. Uls2 is recruited by SUMOylated Euc1, leading to ubiquitin hot spots, which are then extracted by Cdc48 [23]. Furthermore, Uls2 has been implicated in DNA repair and cell cycle control [24,25,26]. Known targets of Slx5-Slx8 include DNA-associated proteins, such as Cse4, Kar9, and Bir1 [17,26,27], as well as transcription factors, such as Mot1 and Matα2 [28,29]. So far, the majority of targets of Uls2 are nuclear proteins. The role of Uls2 in the targeting of cytosolic proteins remains to be elucidated.

To further investigate the impact of Uls2 on the cell proteome, we aimed to identify more targets of Uls2 with a focus on cytosolic proteins. Therefore, we examined a SUMO-chain reporter construct. Here, we demonstrated that SUMO chains lead to the targeting of a cytosolic protein through Uls2 by promoting its nuclear localization. Furthermore, we show that the endogenous yeast proteins Nis1 and Fir1, when in excess of their partner proteins, are targeted by Uls2 in the same manner. Based on these results, we postulate a role for Uls2 in protein quality control (PQC). To find more endogenous substrates of Uls2, we employed a proteomic approach that provided a list of 280 proteins displaying elevated levels in cells lacking functional Uls2. The chaperone Hsp82 was verified as one such target of Uls2 by Western blotting. To investigate the role of Uls2 in PQC, we subjected wild-type cells and cells with a loss of Uls2 function to oxidative stress. Here, we identified 64 proteins that are putative targets of Uls2-mediated PQC, laying the groundwork for future studies.

## 2. Materials and Methods

### 2.1. Growth and Lysis of Yeast Cells

Rich (YP) and selective (S) minimal media with 2% glucose, galactose, or raffinose were prepared as described [30]. The growth of yeast cells in liquid media was monitored by photometric measurement of optical density at 600 nm (OD_600_). Overnight cultures were diluted to an OD_600_ of ~0.25 and grown into exponential phase until 0.5–1 OD_600_. In strains with proteins encoded by genes placed under the control of the promoter P*_GAL1_*, cells were precultured in YP medium containing 2% raffinose followed by growth in YP medium with 2% glucose or 2% galactose for 5 h. Cultures for stress treatments were grown in YP medium containing glucose. After four hours, H_2_O_2_ was added to the culture at a final concentration of 200 µM, followed by further incubation for 45 min at 30 °C. After the incubation, cells were harvested and pellets stored at −20 °C. All *S. cerevisiae* strains used are listed in Table S1. All plasmids used to transform yeast cells are listed in Table S2. Plasmids used in this study are based on different backbones, namely pUC21, YIplac211, pRS316 and YEplac181 [31,32,33].

Different lysis methods were performed depending on the application and the size of the protein. As a standard lysis protocol, yeast pellets were resuspended in Laemmli buffer (50 µL per OD_600_ unit, which corresponds to cells from 1 mL culture with an OD_600_ of 1) and heated for 5 min at 95 °C [34]. Samples used for monitoring Fir1 were prepared using post-alkaline lysis, which included a short treatment with NaOH prior to resuspension in Laemmli buffer [35]. To dissolve Fir1 aggregates, a urea-based glass bead lysis was used as described by Rolli and colleagues [36]. Briefly, cell pellets were resuspended in urea buffer (8 M Urea, 200 mM Tris, pH 7.4) and lysed using glass beads. Then 20% SDS was added to a final concentration of 4% SDS, followed by 5 min of incubation at 65 °C. Debris was pelleted, and the supernatant was stored at −20 °C until further use. Nis1 samples were prepared using alkaline lysis followed by TCA precipitation as follows: cells were resuspended in 1 mL of water, and 166 µL NaOH (2 M) and 12.5 µL β-mercaptoethanol were added. After brief vortexing and 15 min of incubation on ice, proteins were precipitated by the addition of 150 µL TCA (55%). After further incubation on ice, the proteins were pelleted, and the supernatant was removed. Lastly, the protein pellets were resuspended in hydroxyurea buffer (8 M Urea, 5% SDS, 200 mM Tris, pH 6.8) and incubated for 20 min at 45 °C under vigorous shaking [37].

### 2.2. Time-Course Experiment

For time-course experiments, yeast cultures were grown overnight in YP medium containing 2% raffinose and 0.5% glucose. Overnight cultures were diluted to an OD_600_ of 0.4 in YP raffinose and grown for 3 h. Then yeast cells were washed using YP medium containing galactose and diluted to an OD_600_ of 0.5. Cells were imaged immediately after addition of YP galactose medium and after 1 h, 2 h, 3 h, 4 h, and 18 h. Yeast cells were imaged as described in Section 2.6, Fluorescence microscopy.

### 2.3. His-Pulldowns

For His-pulldowns, 150 units OD_600_ of yeast were used. The cells were grown as described above, and pellets were stored at −80 °C until further use. Before the pulldown, pellets were lysed using glass beads with denaturing buffer (8 M urea, 10 mM Tris, 100 mM Na_2_PO_4_, pH 8.0) as described above. His-pulldowns were performed under denaturing conditions using PureCube Co-NTA Magnetic beads (Cube Biotech, Monheim, Germany) according to the manufacturer’s protocol, ‘Purification of His-tagged proteins under denaturing Conditions’. Briefly, after glass bead lysis, the supernatant was transferred onto the equilibrated magnetic beads, followed by binding overnight at room temperature. Then the beads were washed three times using washing buffer (8 M urea, 10 mM Tris, 100 mM Na_2_PO_4_, pH 6.3) and a magnetic rack. Finally, the proteins were eluted using 150 µL elution buffer containing 500 mM imidazole (8 M urea, 10 mM Tris, 100 mM Na_2_PO_4_, 500 mM imidazole, pH 7.4) for 1.5 h. The eluate was collected, and the beads were boiled in Laemmli buffer to collect non-eluted protein.

### 2.4. Western Blots

Protein extracts were separated by SDS-PAGE using acrylamide percentages depending on the size of the proteins under study (Fir1 6%; Nis1 and Hsp82 8%; others 12%), followed by Western blot detection as described [38]. Briefly, the transfer of proteins to nitrocellulose membranes (0.2 µm Amersham Protran, Cytiva, Marlborough, MA, USA) was performed by wet blotting. As a transfer buffer, a boric acid buffer was used (50 mM boric acid, 10% MeOH, pH 8.5). Blotting was performed at 4 °C for 1 h at 100 V and 400 mA. As a size standard, the PageRulerTM Plus Prestained Protein Ladder (Thermo Fisher Scientific, Waltham, MA, USA) was used. After blotting, Revert Total Protein Stain (LI-COR, Lincoln, NE, USA) was used to determine the loading and blotting of samples. Antibodies with their used concentrations are listed in Table S3. LI-COR Odyssey Imager was used to scan membranes, and the signals were quantified and analyzed using Image Studio Ver 5.2 (LI-COR, Lincoln, NE, USA). Statistical analysis and visualization were performed using GraphPad Prism software (v8.4.3, 2020; GraphPad Software, San Diego, CA, USA). A non-paired, parametric Student’s *t*-test was performed as a statistical analysis.

### 2.5. Antibody Production Against Yeast Hsc82-6His Protein

The *Saccharomyces cerevisiae HSC82* gene was cloned into a pET11a-based *E. coli* expression vector using the NdeI and BamHI restriction sites. The recombinant Hsc82-6His protein was produced in strain the BL21 codon plus and purified by Immobilized Metal Chelate Affinity Chromatography (IMAC) using Ni-NTA superflow resin (Qiagen, Hilden, Germany) as described [39]. The purified Hsc82-6His protein was subsequently submitted to BioGenes GmbH (Berlin, Germany) for the generation of polyclonal antibodies in rabbits. The specificity of the antibody was tested using cell extracts derived from deletion mutants (*hsp82Δ* and *hsc82Δ*) as well as from cells expressing Hsp82-GFP (Figure S9). *HSP82* was tagged using a PCR-amplified cassette of the plasmid pYM-N17 [40,41].

### 2.6. Fluorescence Microscopy

Live cells were immobilized before microscopy on 1 mm agarose pads [42]. Cells were analyzed by fluorescence microscopy using the fluorescence microscope Zeiss Axioplan 2 with the corresponding software AxioVision Rel 4.7 (Zeiss, Jena, Germany). For magnification, the objective Zeiss Apochromat 100×/1.4 oil was used. GFP and mCherry/dsRed were used in combination with the following filters: F41-054 HQ-Cy2 HQ480/40 (excitation) Q505LP HQ527/30 (emission), and F41-035 HQ-DsRed HQ565/30 (excitation) Q585LP HQ620/60 (emission). Microscope pictures were stored and processed using OMERO.web 5.31.1 (2005-2026 University of Dundee & Open Microscopy Environment) hosted by the CECAD/CMMC imaging facility.

### 2.7. Proteomics

For whole-cell proteomics, quadruplicates of the strains JD47-13C and YKU148 were grown and treated on individual days, frozen, and processed together. To extract proteins from cells, lysis with glass beads and hydroxyurea buffer (8 M Urea, 4% SDS, 10 mM Tris, pH 7.4) was performed. Cell debris was pelleted and the supernatant was transferred to fresh microcentrifuge tubes. The protein concentration was measured at 280 nm using the Nanodrop One (Thermo Fisher Scientific, Waltham, MA, USA). Samples containing 4 µg of protein were prepared for mass spectrometry (MS) analyses.

Samples were digested following the standard SP3 protocol [43]. Briefly, 20 μg of each washed SP3 bead (Sera-Mag^TM^, Cytiva, Marlborough, MA, USA) was added to each well. This was followed by adding one sample volume of acetonitrile (ACN). The magnetic beads were incubated for 8 min and 2 min on a magnet, followed by two washing steps with 70% EtOH, and one with 100% ACN. Digestion of the samples was performed with 10 μL of 20 ng LysC and 40 ng trypsin dissolved in 50 mM ammonium bicarbonate at 37 °C, 750 rpm overnight. Samples were acidified using 100 μL of 0.1% formic acid (FA), followed by a clean-up with house-made SDB-RPS tips.

Desalted peptides were reconstituted in 5% FA, 2% ACN in HPLC water. Peptide separation was performed on a nanoElute (upgraded, Bruker, Billerica, MA, USA) equipped with a 10 cm PepSep column (150 μm inner diameter, 1.5 μm particle size) with an applied 50 min gradient. Mobile phases were composed of 0.1% FA as solvent A and 0.1% FA in ACN as solvent B. The HPLC system was coupled to a timsTOF pro 2 using a CaptiveSpray source (both Bruker, Billerica, MA, USA). Samples were measured in dia-PASEF mode with automated ion mobility calibrated using three ions of Agilent ESI-Low Tuning Mix following vendor specifications. The dia-PASEF window ranged in dimension 1/k0 0.7–1.45, with 16 × 2 Th windows and in dimension *m*/*z* from 350 to 1250.

The mass spectrometry proteomics data have been deposited in the ProteomeXchange Consortium via the PRIDE partner repository with the dataset identifier PXD080687 [44]. Acquired spectra were analyzed with DIA-NN 1.8.1 using a library-free search against the UniProt *S. cerevisiae* database (April 2024). Mass ranges were set according to the settings of the mass spectrometer; mass deviation was set to 15. Data were further processed using R (V 4.2.2), with the libraries tidyverse, diann, data.table, magrittr, FactoMineR, factoextra and ggplot2, gprofiler, missForest, ggplot2. An in-house modified R script based on the version by V. Demichev was used to calculate MaxLFQ values (Github page, cit MaxLFQ) [45,46]. Data input was filtered for unique peptides, q-Value < 0.01, Lib.Q.Value < 0.01, PG.Q.Value < 0.01, Global.Q.Value < 0.01, Quantity.Quality > 0.7, Fragment.count ≥ 4. Statistical analysis was performed on the MaxLFQ-normalized data with R (V 4.2.2) and Perseus (V.1.6.5.0) and visualized with Instant Clue (V 0.10.10.20210315). Data completeness of 70% was calculated for each group. Missing values were imputed by the random forest algorithm for groups with 70% data completeness; for groups with more than 30% missing values, the random forest algorithm was downshifted by 0.3, with a width of 1.5. Further analysis was performed in Perseus (V 1.6.5.0) and InstantClue (V 0.10.10.20211105) [47]. ANOVA analysis and Student’s *t*-test analysis were performed with an FDR of less than 0.05 and 500 randomizations. Quality control of iRT peptides was performed in Skyline-Daily (V 22.21.391).

## 3. Results

### 3.1. SUMO-Chains Target a GFP-Reporter to the Nucleus

**Figure 1:**
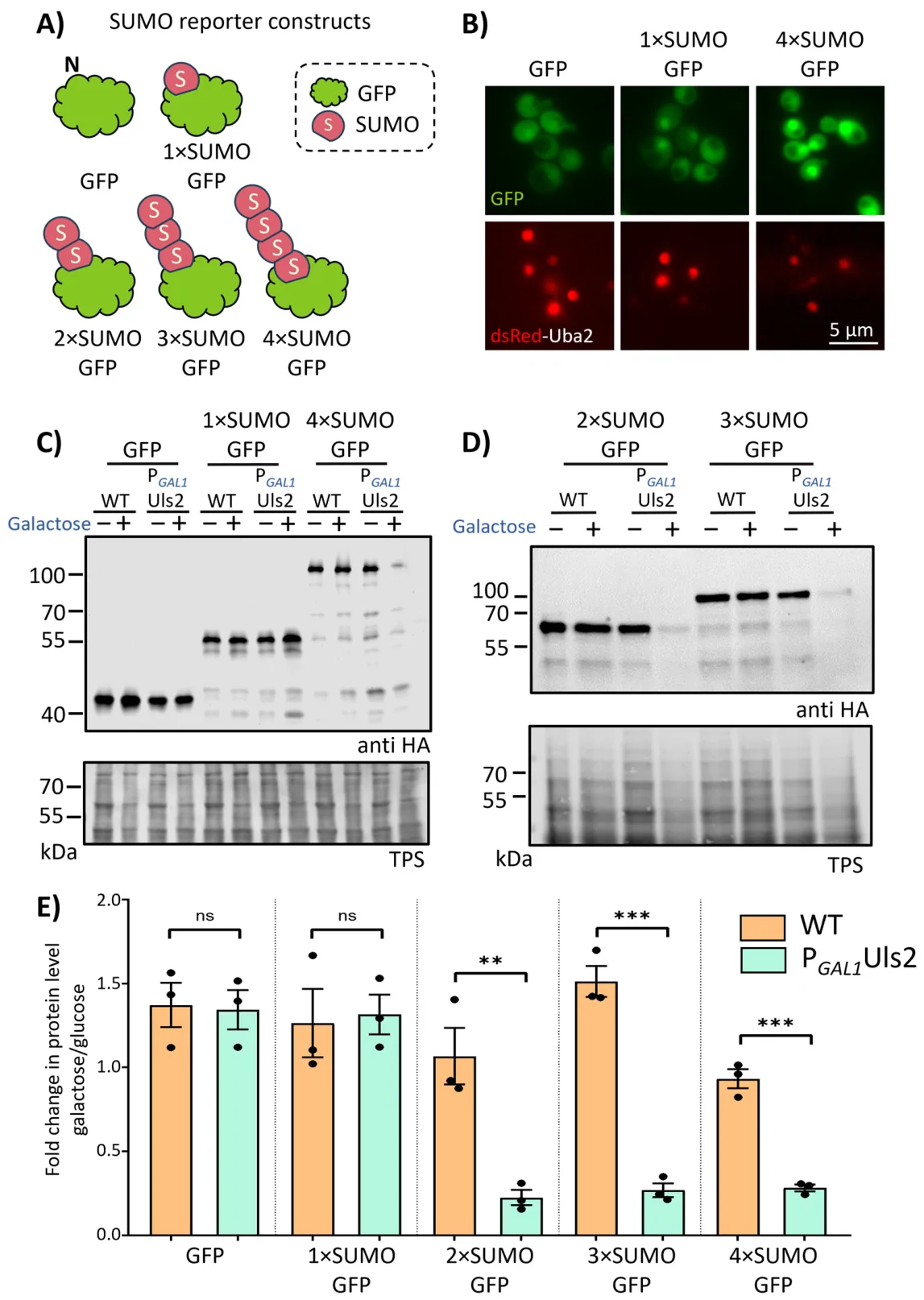
SUMO chains target a GFP reporter to the nucleus. (**A**) Schematic representation of the GFP reporter constructs used in (**B**). GFP tagged N-terminally with linear SUMO fusions ranging from one to four SUMO moieties. (**B**) SUMO chains target a GFP reporter to the nucleus. A *CEN* plasmid expressing GFP with 1×SUMO, 4×SUMO, or without any SUMO was introduced into a wild-type (WT) yeast strain (detected in the upper panels). Cells of the obtained transformants were examined using live-cell fluorescence microscopy after growth in synthetic deficient (S) medium. A plasmid expressing dsRed-Uba2 provided a nuclear marker (detected in the lower panels). Scale bar: 5 µm. (**C**) Protein level of 4×SUMO-GFP is decreased in cells with overexpressed Uls2. Cell lysates of GFP reporters without SUMO, with 1×SUMO, or with 4×SUMO, in the WT or P*_GAL1_ULS2* background, were compared by Western blotting. Cells were lysed after growth for 8 h in synthetic media supplemented with either 2% glucose or 2% galactose. (**D**) Protein levels of 2× and 3×SUMO-GFP are decreased in cells with overexpressed Uls2. Cell lysates of GFP reporters with 2×SUMO and with 3×SUMO in the WT or P*_GAL1_ULS2* background were compared by Western blot. Cells were lysed after growth in S medium, supplemented with either 2% glucose or 2% galactose for 8 h. The original Western blot images are available in the Supplementary Materials. (**E**) Quantifications of the Western blots depicted in (**B**,**C**). The graph shows protein level ratios of cells grown in glucose compared to galactose for the SUMO_n_-GFP constructs in the WT and P*_GAL1_ULS2* backgrounds. Student’s *t*-test: n = 3; ns: *p* > 0.05; **: *p* ≤ 0.01; ***: *p* ≤ 0.001. TPS: Total protein stain.

To investigate if we could force a cytosolic protein to become a Uls2 target, we examined a GFP reporter fused N-terminally either to a single SUMO or to SUMO chains ranging between two and four SUMO moieties (Figure 1A). GFP without any SUMO served as a control. Using fluorescence microscopy, we first explored how these modifications influence the localization of the GFP reporter. We observed a correlation between the length of the SUMO modification and the efficiency of nuclear targeting of the GFP reporter (Figure 1B). Next, we asked if the GFP reporter is targeted by Uls2 upon nuclear localization. Using Western blot analyses, we investigated the levels of the different GFP reporters in a yeast strain where both subunits of Uls2 are encoded from endogenous genes that were brought under the control of the P*_GAL1_* promoter (P*_GAL1_ULS2*). This setup enables the simultaneous repression or induction of both genes by using glucose or galactose (in the absence of glucose) as a carbon source, respectively [48]. Using this setup, we observed that the GFP reporters with one or no SUMO moiety remained stable, independent of Uls2 repression or induction. A GFP reporter carrying a 4×SUMO chain, by contrast, showed a significantly decreased abundance upon Uls2 induction (Figure 1C,E). To determine how many SUMO moieties are required for Uls2 targeting, GFP reporter constructs with two and three SUMO moieties were investigated by Western blot (Figure 1D). Here, we could observe that a chain of two SUMO moieties is sufficient for Uls2 targeting and leads to a similar reduction in protein level as a SUMO chain with four moieties (Figure 1E).

Together, these results showed that the addition of a SUMO chain can promote the relocalization of a GFP reporter from the cytosol to the nucleus, where it is recognized as a substrate by the Uls2 STUbL. This raised the question of whether ubiquitylation by Uls2 is required for retention of the reporter in the nucleus. Therefore, we investigated the localization of the 4×SUMO-GFP reporter in the P*_GAL1_ULS2* strain under shut-off conditions. Here, we found that 4×SUMO-GFP still localizes to the nucleus in the absence of Uls2 (Figure S1), suggesting that STUbL-mediated ubiquitylation is not required for nuclear accumulation of this reporter.

### 3.2. Uls2 Targets the Nuclear Fraction of the Bud Neck Protein Nis1

The above-mentioned finding that SUMOylation promotes nuclear translocation and STUbL-mediated targeting of a cytosolic reporter protein raised the question of whether such a mechanism is relevant for endogenous proteins. Because exposure of SUMO interaction motifs (SIMs) of proteins is known to promote their SUMOylation [4], we focused on cytosolic proteins with known SIMs. To identify such endogenous cytosolic substrates of Uls2, we chose proteins based on three criteria: (1) they contain a SUMO-interacting motif (SIM), (2) there are indications that the proteins are SUMOylated, and (3) they show a cytosolic localization with some nuclear relocalization under certain conditions. The first protein we investigated based on these criteria was Nis1. Nis1 has a SIM and was found as an interactor of SUMO in a yeast-two-hybrid screen, and high molecular weight (HMW)-SUMO conjugates accumulate upon overexpression of Nis1, indicating that it is SUMOylated [9,13]. It has also been shown that, apart from its localization at the bud neck and in the cytosol, Nis1 accumulates in the nucleus upon overexpression or upon deletion of its partner Nba1 [49].

**Figure 2:**
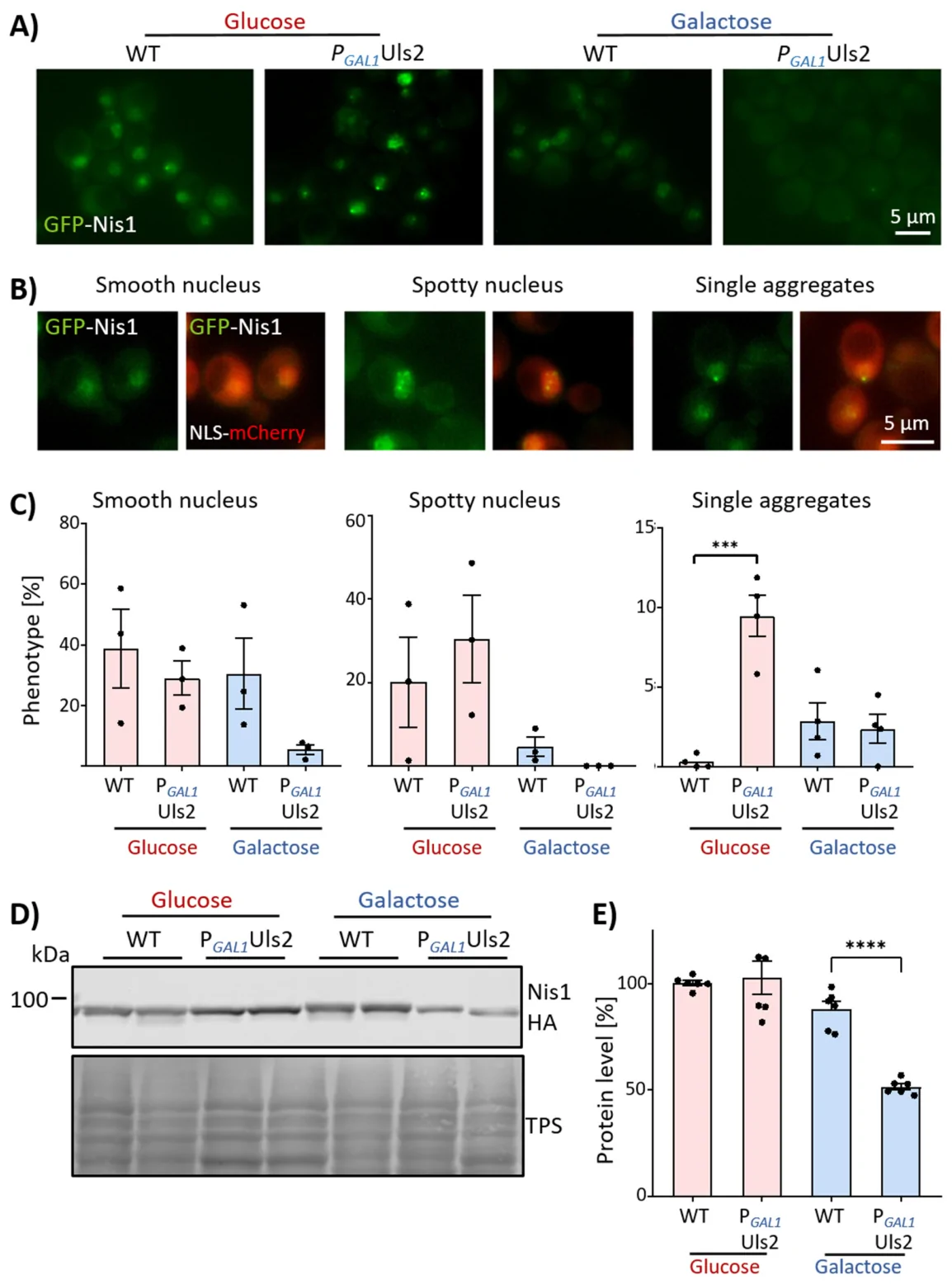
Uls2 targets the nuclear fraction of Nis1. (**A**) Overexpressed Nis1 accumulates in cells when Uls2 is shut off, and disappears in cells with overexpressed Uls2. A cassette containing Nis1 N-terminally tagged with GFP expressed from the constitutive P*_TDH3_* promoter was integrated at the *LEU2* locus in wild-type (WT) or P*_GAL1_SLX5* P*_GAL1_SLX8* (P*_GAL1_ULS2*) background. Live cells were examined using a fluorescence microscope after growth in YP medium supplemented with either 2% glucose or 2% galactose for 5 h. All images were adjusted with +10% brightness and +20% contrast using PowerPoint (Microsoft). Scale bar: 10 µm. (**B**) Nis1 nuclear localization displayed three distinct phenotypes. Nuclear localization phenotypes of Nis1 co-expressed with a nuclear marker NLS-mCherry were characterized as cells with a ‘smooth nucleus’, a ‘spotty nucleus’ or ‘single aggregates’. All images were adjusted with +10% brightness and +20% contrast using PowerPoint (Microsoft). Scale bar: 5 µm. (**C**) Quantitative analysis of the phenotypes depicted in (**B**) of the cells shown in (**A**). (**D**) Protein levels of overexpressed Nis1 are decreased in cells with induced Uls2. Cell lysates of Nis1 expressed from the P*_TDH3_* promoter and tagged with N-terminal GFP and C-terminal HA in the WT and P*_GAL1_ULS2* background were compared by anti-HA Western blotting. Cells were lysed after growth in YP medium supplemented with either 2% glucose or 2% galactose for 5 h. The original Western blot images are available in the Supplementary Materials. (**E**) Quantitative analysis of Western blot shown in (**D**). Student’s *t*-test: n = 6; ***: *p* ≤ 0.001; ****: *p* ≤ 0.0001. TPS: Total protein stain.

To investigate whether nuclear Nis1 is targeted by Uls2, localization and abundance of overexpressed Nis1, N-terminally tagged with GFP, were examined in the WT and P*_GAL1_ULS2* strains using fluorescence microscopy (Figure 2A). We verified the results reported by Perez and Thorner (2019) [49], who showed that overexpressed Nis1 localizes to the nucleus. Furthermore, we observed that Nis1 displayed three distinct nuclear phenotypes: Cells wherein Nis1 was evenly distributed in the nucleus (‘smooth nucleus’), cells with multiple small accumulations of Nis1 throughout the nucleus (‘spotty nucleus’), and lastly cells with one or two aggregates aside from an otherwise even nuclear distribution of the GFP signal (‘single aggregates’) (Figure 2B). These phenotypes varied between the two strains and the two growth conditions. Notably, in comparison to wild-type cells, cells of the P*_GAL1_ULS2* strain with a ‘spotty nucleus’ and with ‘single aggregates’ increased from 20 to 30%, and 1 to 9%, respectively, upon inhibition of Uls2 (glucose media) (Figure 2C). By contrast, upon overexpression of Uls2 (galactose media), cells with a ‘smooth nucleus’ decreased to 5%, and cells with a ‘spotty nucleus’ disappeared completely. In addition, the overall fluorescence, including cytosolic signals, was strongly reduced in these cells (Figure 2A). Together, these data revealed that nuclear Nis1 accumulated under Uls2 shut-off conditions and disappeared upon overexpression of Uls2. To verify the targeting of Nis1 by Uls2, we investigated the abundance of N-terminally GFP-tagged and C-terminally HA-tagged Nis1 using Western blotting. The results support the microscopy data (Figure 2D,E). Quantification of HA signals revealed that Nis1 levels decreased significantly upon Uls2 overexpression (Figure 2E).

To verify the data obtained in strains with inducible Uls2, we investigated endogenous Nis1 tagged with neongreen in the *slx5∆ slx8∆* strain (Figure S2). Here, we found the levels of cytoplasmic Nis1-neongreen to be increased in comparison to the wild-type strain. This result indicates that endogenous levels of the Nis1 reporter are targeted by Uls2 in wild-type cells.

**Figure 3:**
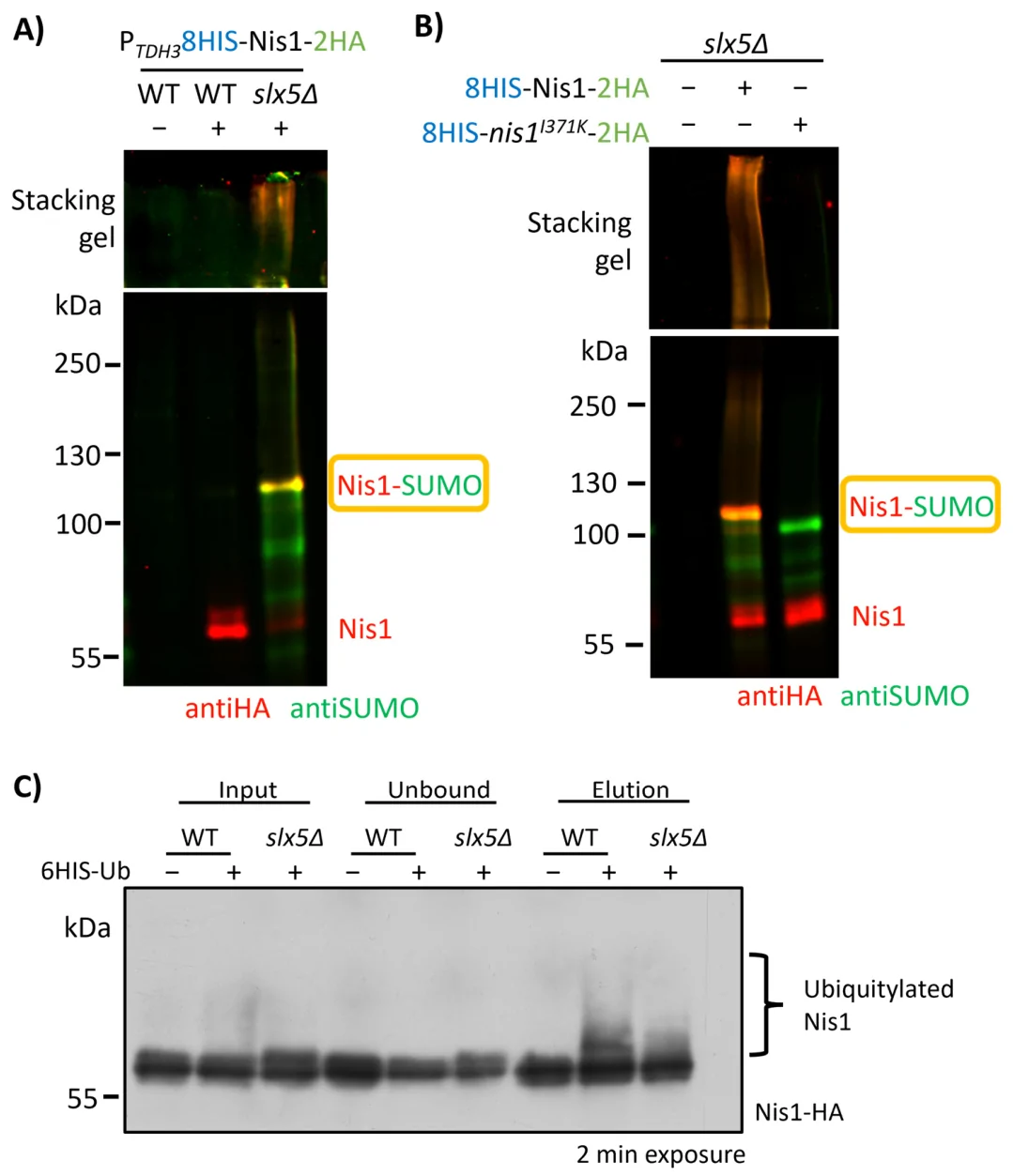
Posttranslational modifications of Nis1 in *slx5Δ* background suggest targeting by Uls2. (**A**) SUMOylated Nis1 is stabilized in *slx5Δ* strain. Pulldown of 8HIS-Nis1-2HA expressed from the P*_TDH3_* promoter brings down a distinct SUMO-modified version of Nis1-2HA from extracts derived from *slx5Δ* cells, in addition to high-molecular-weight (HMW) SUMO conjugates visible in the stacking gel blot. Pulldowns were performed under denaturing conditions in the presence of 8 M urea buffer. Western blot detection employed anti-SUMO and anti-HA antibodies to detect sumoylated forms of Nis1-HA. SUMO and Nis1 are displayed in green and red, respectively, in the Western blot. Bands staining for both antibodies appear yellow indicated by the yellow box. (n = 1). Images show an overlay of anti-SUMO (green) and anti-HA signals (red). (**B**) SUMOylated forms of Nis1 and HMW SUMO-conjugates are abolished upon mutation of its SIM. Pulldown of 8HIS-Nis1-2HA or 8HIS-nis1-I391K-HA expressed from P*_TDH3_* in the *slx5Δ* background. Pulldowns were performed under denaturing conditions in the presence of 8 M urea buffer. Western blot detection was as in panel A (n = 1). (**C**) Ubiquitylated forms of Nis1 are reduced in a *slx5∆* strain. Pulldown of 6HIS-Ubiquitin expressed from P*_GAL1_* promoter copurifies endogenous Nis1-6HA and higher molecular forms of Nis1, which are reduced in the *slx5Δ* background. Pulldowns were performed under denaturing conditions in the presence of 8 M urea buffer. Western blot detection was performed with an anti-HA antibody to detect Nis1-6HA. (n = 2). The original Western blot images are available in the Supplementary Materials.

To verify whether Nis1 is SUMOylated, we performed pulldowns under denaturing conditions (8 M urea) investigating *slx5Δ* cells expressing 8His-Nis1-HA (Figure 3). This experiment yielded a SUMOylated form of Nis1 (Figure 3A), the occurrence of which depended on its SIM, as it was absent when extracts from a strain expressing the Nis1-I391K mutant version were used in the pulldown (Figure 3B). In these experiments, we also observed a striking accumulation of HMW SUMO-conjugates in the stacking gels when pulldowns were performed with extracts from *slx5∆* (Figure 3A,B). To test whether we can demonstrate Uls2-dependent ubiquitylation of Nis1, we performed pulldown experiments with cells expressing 6His-ubiquitin and endogenous levels of Nis1-6HA. In cells with wild-type Uls2, we detected a ubiquitin smear on Nis1, and a reduction of ubiquitylation in *slx5∆* (Figure 3C). Both the accumulation of SUMOylated Nis1 and the reduction of ubiquitylated Nis1 in *slx5∆* further supported the targeting of Nis1 by endogenous Uls2 in wild-type cells.

### 3.3. Uls2 Targets Distinct Nuclear Aggregates of the Bud Neck Protein Fir1

**Figure 4:**
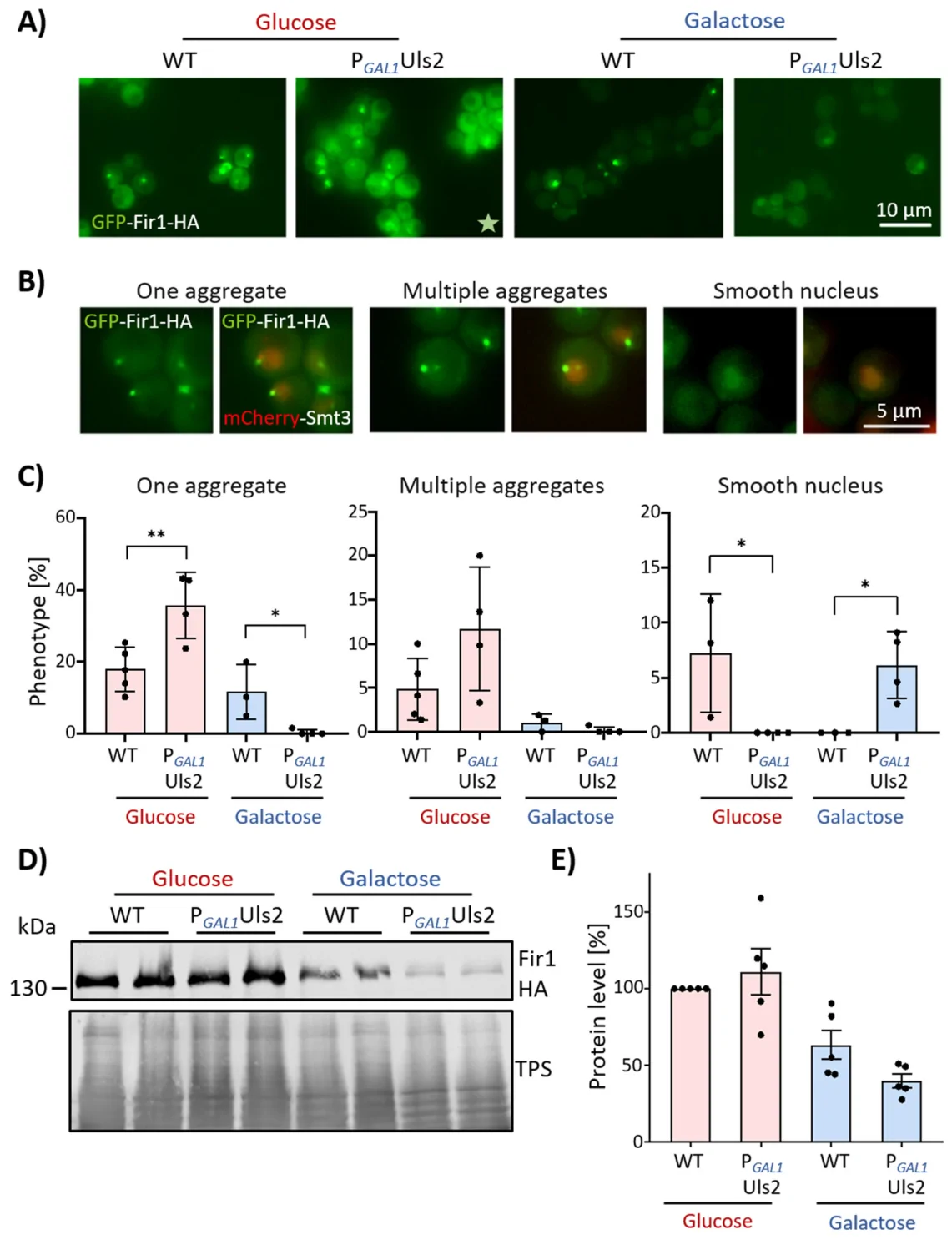
Uls2 targets the nuclear fraction of Fir1. (**A**) Overexpressed Fir1 accumulates in cells when Uls2 is shut off and disappears in cells with overexpressed Uls2. A cassette expressing Fir1 N-terminally tagged with GFP and C-terminally tagged with HA under the constitutive P*_TDH3_* promoter was integrated at the *LEU2* locus in wild-type (WT) or P*_GAL1_SLX5* P*_GAL1_SLX8* (P*_GAL1_ULS2*) background. Live cells were examined using fluorescence microscopy after growth in YP medium supplemented with either 2% glucose or 2% galactose for 5 h. All images, except for P*_GAL1_ULS2* with glucose (indicated by a green star), were adjusted with +30% brightness and +20% contrast using PowerPoint (Microsoft). For direct visual comparison of all four images with identical brightness and contrast settings, see Figure S7. Scale bar: 10 µm. (**B**) Fir1 nuclear localization displayed three distinct phenotypes. Nuclear localization phenotypes of Fir1 co-expressed with mCherry-*SMT3,* serving as a nuclear marker, were characterized as cells with ‘one aggregate’, with ‘multiple aggregates,’ or with a ‘smooth nucleus’. All images were adjusted with +30% brightness and +20% contrast using PowerPoint (Microsoft). Scale bar: 5 µm. (**C**) Quantitative analysis of the phenotypes depicted in (**B**) of the cells shown in (**A**). (**D**) Protein levels of overexpressed Fir1 are decreased in cells with overexpressed Uls2. Lysates of cells overexpressing Fir1 from P*_TDH3_* tagged N-terminally with GFP and C-terminally with HA in the WT and P*_GAL1_ULS2* background were compared by anti-HA Western blotting. Cells were lysed after growth in YP medium supplemented with either 2% glucose or 2% galactose for 5 h. The original Western blot images are available in the Supplementary Materials. (**E**) Quantitative analysis of Western blot shown in (**D**). Student’s *t*-test: n = 5; *: *p* ≤ 0.05; **: *p* ≤ 0.01. TPS: Total protein stain.

Based on the same criteria as for Nis1, we chose Fir1 as another candidate protein. (1) Fir1 contains a SIM [9,13]. (2) Fir1 binds to SUMO via its SIM and is SUMOylated [9,50]. (3) Fir1 is a mainly cytosolic protein that shuttles between the bud tip and the bud neck, where it interacts with SUMOylated septins before it relocates between the split septin rings, promoted by interaction with the Spa2 protein [51]. Fir1 levels are then downregulated at the end of cytokinesis by the Cdh1-activated ubiquitin ligase APC [52]. In the absence of Spa2, a fraction of Fir1 relocalizes to the nucleus, as we could detect in a *cdh1∆* strain background (Figure S3). Similarly, as for Nis1, overexpressed N-terminally GFP-tagged Fir1 was investigated in the wild-type and P*_GAL1_ULS2* strains using fluorescence microscopy (Figure 4A). Fir1 predominantly localized in the nucleus when overexpressed. Like Nis1, Fir1 displayed distinct nuclear phenotypes depending on the strain and growth condition. Three different nuclear phenotypes were observed for Fir1: (1) cells with one large aggregate in or at the side of the nucleus (‘one aggregate’), (2) cells with more than one large aggregate (‘multiple aggregates’), and (3) cells with an even distribution of GFP-Fir1 throughout the nucleus (‘smooth nucleus’) (Figure 4B). Shut-off of Uls2 (glucose media) increased both aggregate phenotypes, whereas Uls2 overexpression (galactose media) abolished Fir1 aggregates nearly completely (Figure 4C). This indicated that Uls2 specifically targets Fir1 aggregates. In order to study the stability of these aggregates, we performed time-course experiments monitoring GFP-Fir1 in the P*_GAL1_ULS2* strain and, as a control, in the *slx5Δ* strain after galactose induction (Figure S4). This approach revealed a reduction in the number of Fir1-containing aggregates upon galactose induction of Uls2 expression starting after 4 h. After 18 h of induction, nearly no Fir1 aggregates were detectable in the P*_GAL1_ULS2* strain. In contrast, the number of aggregates remained stable in *slx5Δ* after addition of galactose, verifying that targeting of Fir1 depends on induction of Uls2. Furthermore, we investigated GFP-Fir1-HA signals and localization in *slx5Δ* compared to wild-type cells (Figure S5). This revealed an accumulation of aggregates in *slx5Δ* cells compared to the wild type. Additionally, we analyzed the Fir1-SIM mutant and found that it did not form any aggregates in either wild-type or *slx5Δ* cells. Instead, we observed increased cytosolic levels of GFP-Fir1^I760K^ in *slx5Δ* cells (Figure S5).

To support the microscopy results, we analyzed the levels of GFP-Fir1-HA in the P*_GAL1_ULS2* strain (Figure 4D). Anti-HA Western blotting also supported a reduction of Fir1 under Uls2 overexpression conditions, although the effect was more evident in the microscopy experiments (Figure 4A). We presume that this is due to the aggregates of Fir1, which may have partially escaped detection in the SDS-PAGE/Western blot experiment.

To further investigate if Fir aggregates indeed escaped our detection due to the lysis procedure, we compared Fir1 levels in *slx5Δ* cell extracts produced either by boiling or by urea-based protein extraction (Figure S6). In comparison to the wild type, reduced amounts of Fir1 were detected in boiled extracts from *slx5Δ* cells (Figure S6A,B). In contrast, the urea-based extraction revealed higher levels of Fir1 in *slx5Δ* cells compared to the wild type (Figure S6C,D). These data support our interpretation that Fir1 aggregates are, at least in part, not soluble using our standard lysis methods.

So far, we have shown that three proteins, the SUMO_2-4x_-GFP reporter, Nis1 and Fir1, localize to the nucleus and are targeted by Uls2. For Fir1, we could observe strong aggregate formation. These aggregates are targeted by Uls2. This led us to ask if SUMOylation as well as the SIM of Fir1 cause aggregate formation.

### 3.4. Fir1 Aggregates Colocalize with SUMO and Their Formation Is SUMOylation and SIM-Dependent

**Figure 5:**
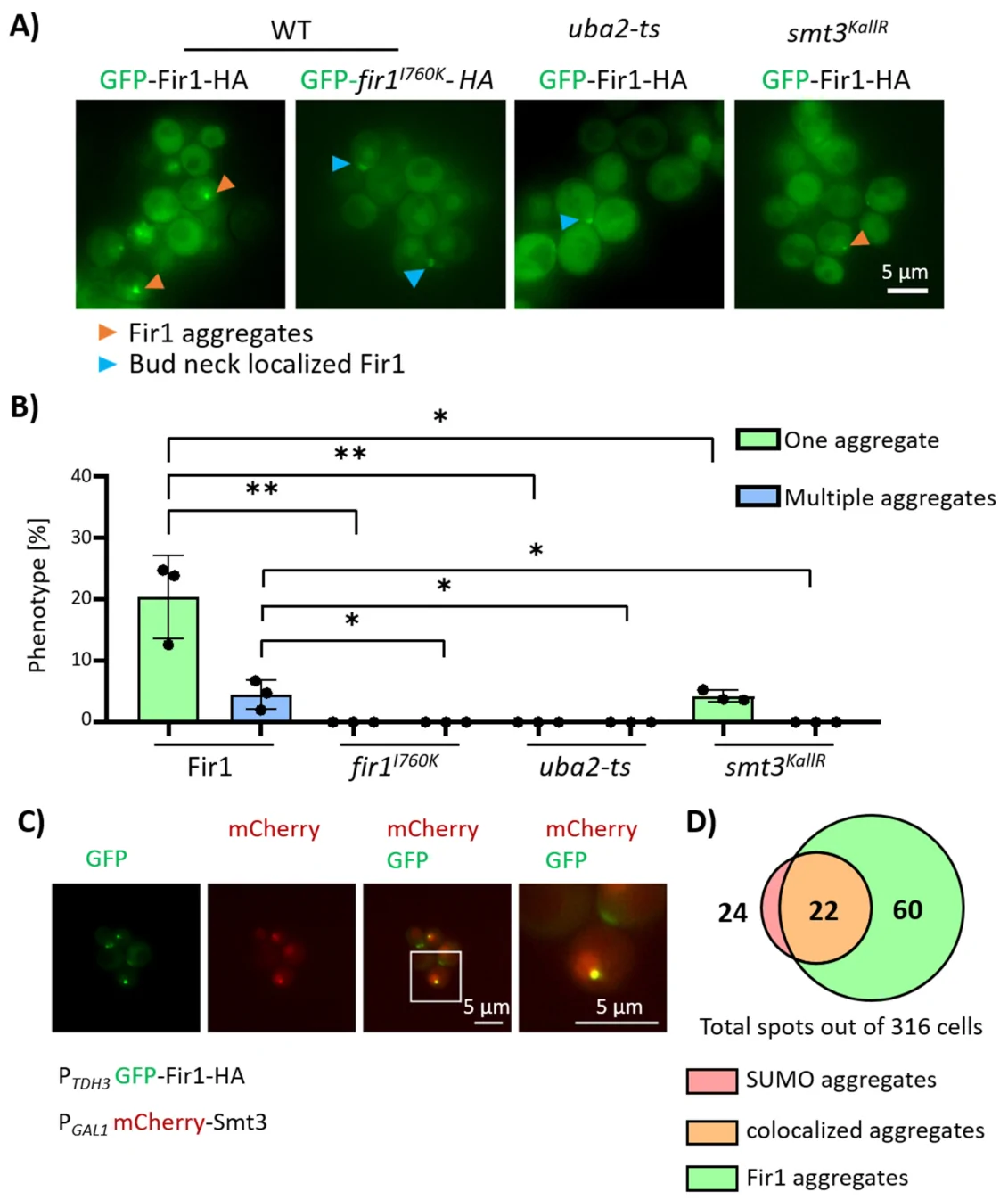
Fir1 aggregates colocalize with SUMO and their formation is SUMOylation- and SIM-dependent. (**A**) Aggregate formation of Fir1 depends on its SIM, on SUMOylation, and is promoted by polySUMOylation. A cassette expressing Fir1 or the Fir1 SIM mutant Fir1-I760K, N-terminally tagged with GFP and C-terminally tagged with HA, expressed from the constitutive P*_TDH3_* promoter, was integrated at the *LEU2* locus in the wild-type (WT), the E1 enzyme mutant *uba2-ts*, or the polySUMOylation-deficient strain *smt3^KallR^*. Live cells were examined using fluorescence microscopy after growth in YP medium supplemented with 2% glucose. All images were adjusted with +30% brightness and +20% contrast using PowerPoint (Microsoft). Scale bar: 5 µm. (**B**) Quantitative analysis of the phenotypes depicted in Figure 3B of the cells shown in (**A**). (**C**) SUMO colocalizes with Fir1 aggregates. A high-copy (2 µm) and *URA3*-marked plasmid encoding SUMO tagged with mCherry, expressed from the P*_GAL1_* promoter, was introduced into a strain with integrated P*_TDH3_*GFP-FIR1-HA. Live cells were examined using fluorescence microscopy after growth in synthetic (S) medium lacking uracil, supplemented with 2% galactose. All images were adjusted with +30% brightness and +20% contrast using PowerPoint (Microsoft). Scale bar: 5 µm. (**D**) Quantitative analysis of Fir1 and SUMO aggregates shown in (**C**). Student’s *t*-test: n = 3; *: *p* ≤ 0.05; **: *p* ≤ 0.01.

To investigate the involvement of Fir1’s SIM and SUMOylation in aggregate formation, localization of GFP-Fir1 was examined in different mutants using fluorescence microscopy (Figure 5A). The role of the SIM of Fir1 was investigated by using a SIM mutant (*fir1^I760K^*), wherein the hydrophobic core of the SIM was disrupted by a positively charged lysine. Strikingly, no aggregates were detectable in cells expressing GFP-Fir1-I760K. The role of SUMOylation was investigated using a temperature-sensitive strain (*uba2-ts*) expressing a functionally impaired Uba2, which is a subunit of the SUMO-activating enzyme (E1) heterodimer Uba2-Aos1 [53]. As for the SIM mutant, and equally strikingly, GFP-Fir1 aggregate formation was abolished by the *uba2* mutation. We conclude that the SIM of Fir1 as well as SUMOylation are necessary for Fir1 aggregate formation. Next, we asked if polySUMOylation is also required for aggregate formation. To this end, we employed a SUMO mutant that is unable to form chains because all lysine residues are mutated to arginine (*smt3^KallR^*) [54]. In this mutant, aggregate frequency was reduced, and the intensity of the observed aggregates decreased compared to the wild type. These observations suggest that, even though not strictly necessary, polySUMOylation promotes Fir1 aggregate formation or stability.

Lastly, we asked if SUMO itself is part of the aggregates. Therefore, we co-expressed mCherry-SUMO together with GFP-Fir1 and investigated their localization (Figure 5C). Indeed, we could observe that, aside from its localization throughout the nucleus, mCherry-SUMO accumulated at nuclear sites of aggregated Fir1. Taken together, our data show that Fir1 forms SIM- and SUMOylation-dependent aggregates, which include SUMO itself. These aggregates are then targeted by Uls2.

### 3.5. Proteomic Approach Reveals Impact of Uls2 Loss of Function on the Proteome

So far, we have shown that Uls2 targets a SUMO chain reporter as well as two endogenous proteins. Our next goal was to assemble a wider list of putative cellular Uls2 targets, including cytosolic proteins. To achieve this, we chose a mass spectrometry-based proteomic approach. To minimize proteome changes through the use of different carbon sources, we chose to compare the proteome of the wild-type strain with a *slx5Δ* strain, the latter of which stabilizes targets of Uls2. Because we noticed, as described above, that Nis1 and Fir1 are targeted when they are overexpressed and are in excess of their partner protein, we speculated that Uls2 might serve as part of a quality control mechanism for proteins either lacking proper interaction with partner proteins or with abnormal folding or localization due to other reasons. We hypothesized that this quality control mechanism might include proteins that become damaged due to cellular stress.

**Figure 6:**
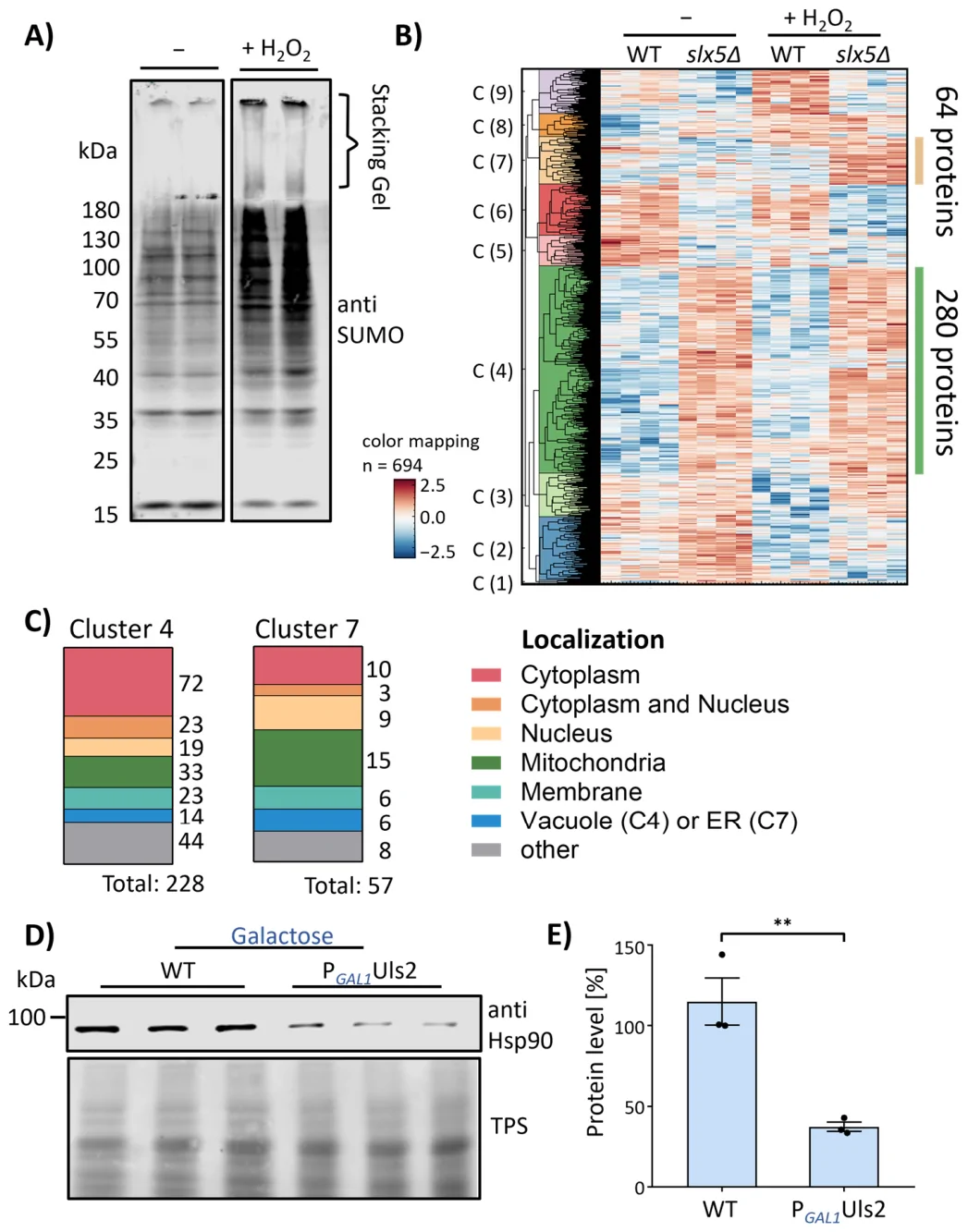
Proteomic approach reveals impact of Uls2 (Slx5-Slx8) on proteome. (**A**) SUMOylation is increased in cells treated with H_2_O_2_. Lysates of wild-type (WT) cells, either untreated or treated with 200 µM H_2_O_2_, were compared by anti-SUMO/Smt3 Western blotting. Cells were grown in YP medium for 4 h, then treated with 200 µM H_2_O_2_ and grown for another 45 min. The lanes 1–2 and 3–4 are biological replicates. (**B**) *Slx5∆* influences the proteome landscape. Hierarchical clustering of proteins obtained from whole-cell mass spectrometry. Whole-cell extracts of WT and *slx5Δ* cells, with and without oxidative stress, were prepared and analyzed by mass spectrometry. (**C**) Proteins of clusters 4 and 7 show diverse localizations. The graphic shows the number of proteins from cluster 4 or 7 associated with the respective localization (see legend). Annotations for subcellular localizations of the proteins in clusters 4 and 7 were downloaded using Uniprot ID mapping (accessed 25 June 2026). Proteins without annotations of subcellular localization were omitted from analysis. (**D**) Levels of the Hsp90 chaperones are decreased in cells with overexpressed Uls2. Hsp90 levels in WT and P*_GAL1_ULS2* cells were compared by anti-Hsc82 (detecting both Hsc82 and Hsp82, see Figure S9) Western blotting. Cells were lysed after growth in YP medium supplemented with 2% galactose for 5 h. Antibody specificity was verified using deletion mutants (Figure S9). Lanes 1–3 and 4–6 are from biological replicates. (**E**) Quantitative analysis of Western blot is shown in (**D**). n = 3; Student’s *t*-test: **: *p* ≤ 0.01. TPS: Total protein stain. The original Western blot images is available in the Supplementary Materials.

To investigate whether Uls2 also targets proteins upon stress, we compared untreated WT and *slx5Δ* cells with cells subjected to oxidative stress (200 µM H_2_O_2_). First, we assessed the effects of oxidative stress on SUMOylation using Western blotting. This analysis revealed that SUMOylation was significantly increased upon oxidative stress treatment (Figure 6A and Figure S8), similar to what was observed for SUMO2/3 conjugates in mammalian cells [55]. In particular, HMW-SUMO conjugates, some of which remain in the stacking gel, accumulated to high levels. To identify substrates of Uls2 with and without stress conditions, we performed a whole-cell proteome analysis. Hierarchical clustering of the obtained MS data revealed that the deletion of *SLX5* drastically altered the proteome landscape (Figure 6B and Table S4). Cluster 4 and 6 contained proteins with increased and decreased abundance upon *SLX5* deletion, respectively. Cluster 4 included 280 proteins that are potential targets of Uls2 because their levels are significantly increased in *slx5∆* compared to wild-type cells. Interestingly, cluster 7 included 64 proteins, the levels of which increased in *slx5Δ* only upon stress treatment, indicating that these proteins could be targets of a Uls2-mediated quality control mechanism acting upon oxidatively damaged proteins. To examine the effect of Uls2 on cytosolic proteins, we analyzed the subcellular localization of proteins in clusters 4 and 7 (Figure 6C and Table S5). We found that both clusters include cytosolic proteins. In cluster 4, the cytosolic proteins make up the largest group with a total of 72 proteins (31.6%). Additionally, the analysis revealed 33 mitochondrial proteins that are impacted by deletion of *SLX5*. We conclude that Uls2 is able to influence the protein abundance of a large set of proteins with diverse subcellular localization.

To verify that proteins from cluster 4 are potential targets of Uls2, we selected the chaperone Hsp90 for further analysis because it was already reported as a substrate of SUMOylation as well as ubiquitylation [50,56,57,58]. In *S. cerevisiae*, there are two paralogs of Hsp90, namely Hsc82 and Hsp82, which are closely related but distinctly regulated by heat stress [59,60]. We employed a polyclonal antibody that we raised against Hsc82, which recognizes both Hsc82 and Hsp82 (Figure S9), to monitor whether levels of endogenous Hsp90 are affected by Uls2 overexpression. Western blot analysis using this antibody revealed a strong reduction of Hsp90 upon overexpression of Uls2 (Figure 5D,E). These results indicated that our proteome approach is suitable to identify cellular targets of Uls2. Our proteome data point to a large pool of Uls2 targets with a subset of stress-induced Uls2 substrates.

## 4. Discussion

In this study, we show that SUMO fusion mediates the nuclear localization of GFP reporters (Figure 1). The endogenous cytosolic proteins Nis1 and Fir1, similarly, relocalize to the nucleus in a SUMOylation-dependent manner when they lack their partner subunits (Figure 2 and Figure 3 and Figure S3) [49]. In line with these results, SUMO has been reported to be involved in the nuclear localization of a variety of proteins. Examples include the nuclear import of the Polo-like Kinase 1 and Lipin-1α, which is facilitated by their SUMOylation [61,62]. Furthermore, for the insulin-like growth factor-1 receptor (IGF-1R), SUMOylation was shown to facilitate its nuclear localization [63]. IGF-R1 was shown to be SUMOylated by RanBP2 at the nuclear pore complex, leading to its nuclear import [64]. For most proteins, the mechanisms by which SUMOylation promotes or enhances nuclear import or export remain largely unknown [7]. To identify karyopherins and investigate mechanistic details of SUMO-promoted nuclear translocation will therefore be interesting topics for future studies.

Another aim of this study was to identify new targets of the STUbL Uls2 with a special focus on cytosolic proteins that are SUMOylated and then translocated to the nucleus. Our results from fluorescence microscopy and Western blot analyses identified three novel targets of Uls2: Nis1, Fir1, and Hsp82. Additionally, we could show, using linear SUMO_n_-GFP reporters, that their relocalization to the cell nucleus leads to their proteolytic targeting by the STUbL Uls2. Two SUMO moieties are sufficient for this targeting mechanism. A similar requirement for at least two SUMO moieties was previously observed for the human STUbL RNF4 [65].

**Figure 7:**
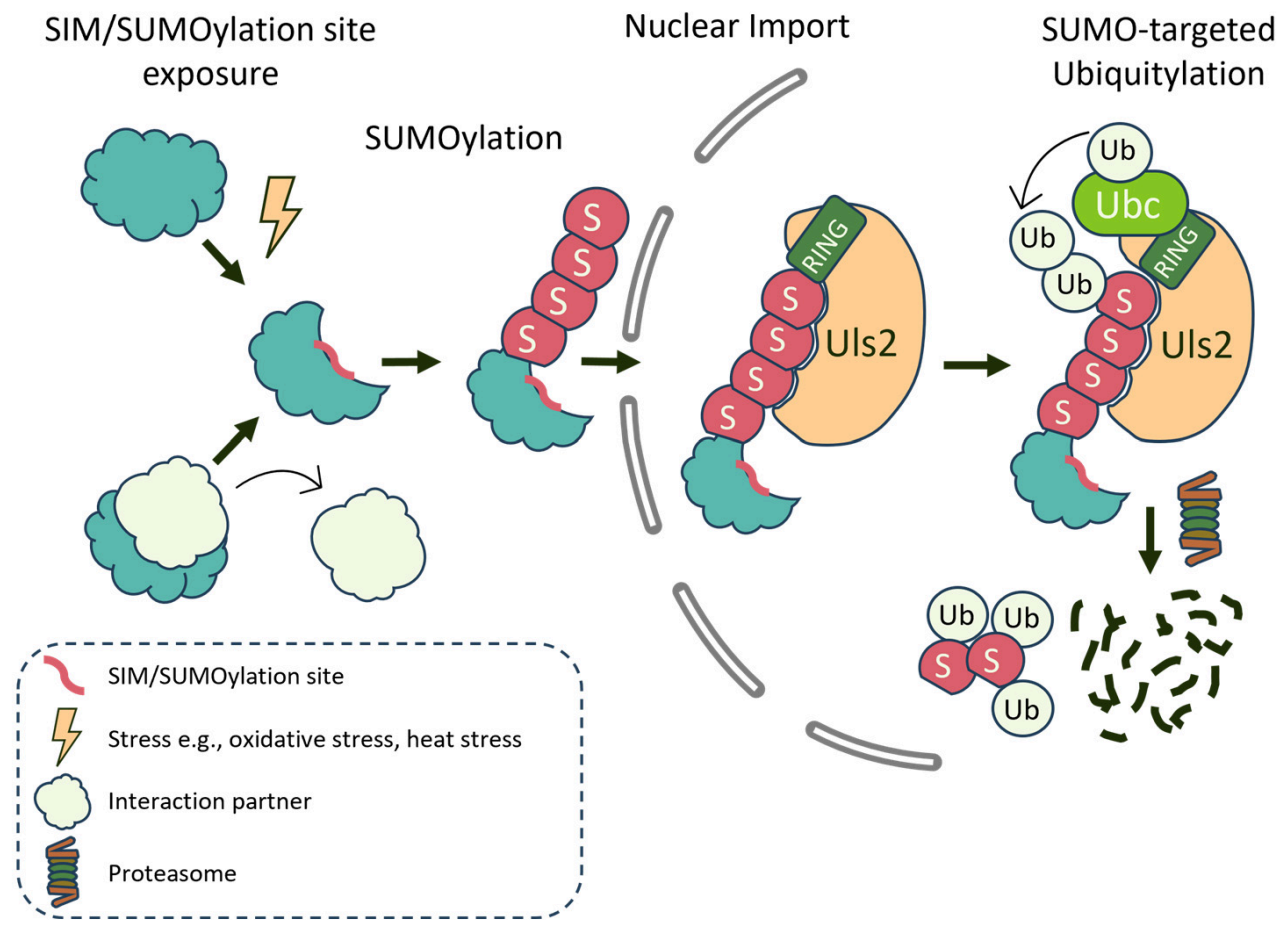
Hypothetical model of the Uls2 STUbL in cellular quality control. Uls2 serves as part of a quality control (QC) mechanism for proteins or their subunits containing SIM- and SUMOylation sites that are usually hidden within the structure. In the absence of a partner polypeptide of a protein or under folding stress, an internal SIM or SUMOylation site is exposed. This site promotes SUMOylation of the protein and subsequently its relocalization to the nucleus. There, the SUMO chain is recognized by Uls2, leading to ubiquitylation. The ubiquitin tag then promotes recognition and degradation of the protein by the proteasome.

Interestingly, both Nis1 and Fir1 were targeted by Uls2 under conditions of their overexpression, causing their synthesis in excess of their partner proteins Nba1 and Spa2, respectively (Figure 1 and Figure 2). Additional evidence indicated that absence of Uls2 (*slx5∆*) also has effects on endogenous levels of these proteins or their SUMO-dependent ubiquitylation (Figure 3). Since targeting of both proteins was more striking when they were overexpressed relative to their partner protein, we hypothesize that Uls2 might serve as part of a quality control (QC) mechanism mediating the degradation of unmatched or defective proteins (Figure 7). We speculate that such a disbalance relative to a partner protein (simulated by overexpression), or misfolding caused by stress, might lead to an exposure of hidden SIMs or SUMOylation motifs. The localization of the SIM in Fir1 (residues 759–767) [9] appears to be consistent with this interpretation, as it is part of the segment found to mediate binding to Spa2 (residues 741–876) [51]. No reliable structural information on Nis1, alone or in complex with Nba1, is currently available. Further work is therefore required to verify whether the absence of Nba1 leads to an exposure of the Nis1 SIM or putative SUMO attachment sites.

In our model, the exposure of SIMs leads to SUMOylation of a protein and its relocalization into the nucleus. SIMs are known to enhance SUMOylation of proteins because they mediate interaction with SUMO-loaded SUMO E2 enzyme Ubc9 [4]. Transfer of SUMO to substrates is promoted by SUMO E3 ligases. The ligases Siz1 and Siz2 have overlapping substrates, including cytoplasmic proteins, such as septins [66,67], and therefore are likely mediators of the QC mechanism described in the present work. Siz1 is the main SUMO ligase for septins and, interestingly, itself a protein that shuttles between the cell nucleus and the cytosol depending on the stages of the cell cycle. Uls2 was shown to downregulate nuclear Siz1 when the cells enter mitosis [68]. In our model, the nuclear Uls2 recognizes the polySUMO tag of proteins relocalized from the cytosol to the nucleus and mediates their ubiquitylation. Subsequently, the proteins are degraded by the proteasome. Consistent with this hypothesis, we demonstrated that Nis1 and Fir1 were targeted to the nucleus upon overexpression, were prone to aggregation, and were targeted by Uls2. For Nis1, we observed in a previous study, that accumulation of HMW-SUMO conjugates in a Nis1 overexpression strain depends on its SIM [9]. For Fir1, we could show in this study that formation of aggregates is both SUMO- and SIM-dependent. Interestingly, the Uls2 subunit Slx5 has been observed in nuclear foci by Cook and colleagues [19]. These foci appear to be similar to the aggregates that we observed for Fir1, suggesting a common mechanism of their formation. Furthermore, it has been shown that the human functional homolog of Uls2, RNF4, is involved in the regulation of stress granule dynamics and has been proposed to mediate an adaptive stress response pathway [69]. STUbL action in such protein QC pathways may either occur early on the soluble form of a protein, thus promoting its degradation before it engages in aggregate formation, or on proteins already present in nuclear aggregates. Formation of the latter is apparently promoted by poly-SUMOylation, as observed for Fir1 (Figure 5). SUMOylation and ubiquitylation of proteins present in such aggregates might trigger their disassembly by the SUMO- and ubiquitin-targeted segregase Cdc48, facilitating subsequent degradation by the proteasome [70,71,72,73,74]. Our time-course experiments monitoring GFP-Fir1 levels suggest that Uls2 can act on preexisting nuclear aggregates because they start to disappear four hours after Uls2 induction.

Uls2 has previously been described as being involved in QC of the Mot1 protein [28]. However, Nis1 and Fir1 are, unlike Mot1, not inherently nuclear proteins. Both are only nuclear upon overexpression or absence of their respective partners [49]. It appears as if Nis1 and Fir1 are solely shuttled to the nucleus to be targeted by Uls2 as part of a QC system. A similar mechanism has been reported before, according to which stress-induced misfolded cytosolic proteins are transported into the nucleus through chaperone-assisted nuclear import, where they are sequestered by the aggregase Btn2 into intra-nuclear quality departments (INQs) [75]. Malinovska et al. (2012) discussed that relocalization of misfolded proteins could serve as a mechanism to ensure an equal distribution across the PQC systems [76]. Furthermore, it has been reported that mitochondrial proteins, upon an import deficiency, can relocalize to the nucleus where they are targeted to the proteasome as part of a so-called nuclear-associated mito-protein degradation (mitoNUC) [77]. Interestingly, it has been described that mitochondrial import deficiency and impaired proteostasis lead to SUMOylation of diverse mitochondrial proteins [78], some of which were also detected in our proteomic approach, such as Tim9 or Nde1 (Table S4), suggesting that at least a part of mitoNUC might be SUMO- and Uls2-dependent.

Both Nis1 and Fir1 have a timed inhibitory function at the bud neck in the cell cycle, which may justify their need for a relocalization. Nis1 is involved in inhibiting the reuse of an old bud site, and Fir1 is involved in inhibiting cytokinesis if certain processes are not yet completed [79,80]. Fir1 overexpression has direct consequences on the growth of cells, which become clustered because cytokinesis is not finished properly [79]. Uls2-mediated quality control might serve as a mechanism to fine-tune Nis1 and Fir1 levels by eliminating molecules that are not interacting with their appropriate partners to avoid any unwanted side effects.

To find putative additional endogenous substrates of the Uls2 STUbL, we employed an *slx5∆* strain to compare its proteome with a wild-type strain either under unstressed conditions or after induction of oxidative stress. Based on this approach, we assembled a list of proteins, levels of which are changed in the absence of Uls2 under one of these conditions (Figure 6, Table S4). This approach, however, not only identifies direct substrates of Uls2 but also proteins, levels of which changed as a consequence of regulatory responses caused by the absence of the STUbL. Impairment of Uls2 function is known to have a rather wide range of effects on chromatin, DNA damage repair, cell cycle, transcription, and stress tolerance [20,23,26,71,81]. The whole-cell proteomic data summarized in Table S4 provide a rich resource for further studies on the cellular functions and substrates of Uls2.

## 5. Conclusions

We identified three novel endogenous cellular targets of Uls2 and also provided a list of additional putative targets that remain to be studied in the future. We propose that Uls2 serves as part of a QC system for proteins with exposed SIMs or SAS. The SUMO-dependent mechanism of relocalization for QC parallels other known QC systems and expands our understanding of QC for cytosolic proteins. Details of the mechanism of nuclear import of such SUMOylated or SIM-containing proteins remain to be elucidated.

## Data Availability

The original contributions presented in this study are included in the article/Supplementary Material. Further inquiries can be directed to the corresponding authors.

## Supplementary Materials

The following supporting information can be downloaded at https://www.mdpi.com/article/10.3390/biom16091322/s1, Table S1: Yeast Strains used in this study; Table S2: Plasmids used in this study; Table S3: Antibodies used in this study; Table S4: Hierarchical Cluster (Excel); Table S5: Localization Cluster 4 and Cluster 7 (Excel); Figure S1: PolySUMO reporter localizes to the nucleus when *SLX5* and *SLX8* are shut off; Figure S2: Endogenous Nis1 accumulates to higher levels in a *slx5∆ slx8∆* strain; Figure S3: Fir1 relocalizes to the cell nucleus in the absence of Spa2; Figure S4: Fir1 levels decrease after Uls2 induction; Figure S5: Fir1 aggregates accumulate in a *slx5Δ* strain; Figure S6: Levels of insoluble Fir1 are increased in a *slx5Δ* strain; Figure S7: Comparison of microscope images from Figure 5A with images adjusted uniformely to show the much stronger GFP-Fir1 signals after repression of Uls2; Figure S8: Oxidative stress increases high-molecular-weight SUMO conjugates; Figure S9: Verification of Hsp90 antibody specificity; Original western blot images of the Figure 1, Figure 2, Figure 3, Figure 4 and Figure 6 are available.

## Abbreviations

The following abbreviations are used in this manuscript:

PQC	Protein Quality Control
QC	Quality Control
STUbL	SUMO-targeted ubiquitin ligase
SUMO	Small Ubiquitin related modifier
