# Supplementary material for: Role of Nuclear SUMO-Targeted Ubiquitin Ligase Uls2 (Slx5-Slx8) in Quality Control of Cytosolic Proteins
Source: Biomolecules. 2026 Sep 11;16(9):1322. doi: 10.3390/biom16091322 (PMC13604690; doi:10.3390/biom16091322)
Supplement: Supplementary file 1 [file biomolecules-16-01322-s001.zip › Figures with Full Blots & Pictures.pdf]

Figures with full western blots & full unedited microscope pictures

Figure 1

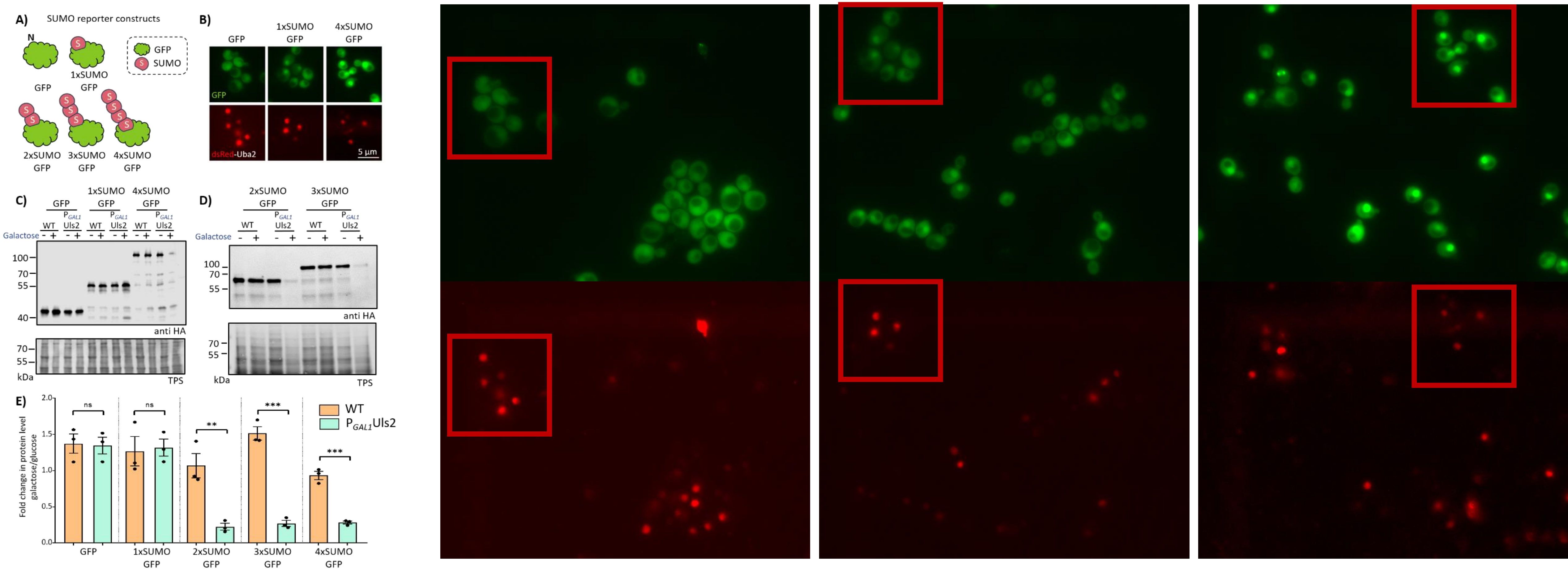

A)
SUMO reporter constructs
N
GFP
SUMO
GFP
1xSUMO GFP
2xSUMO GFP
3xSUMO GFP
4xSUMO GFP
B)
GFP
1xSUMO GFP
4xSUMO GFP
dsRed-Uba2
5 µm
C)
D)
2xSUMO GFP
3xSUMO GFP
WT
Uls2
Galactose
anti HA
TPS
kDa
100
70
55
40
E)
Fold change in protein level galactose/glucose
ns
**
***
WT
Uls2

Figure 1

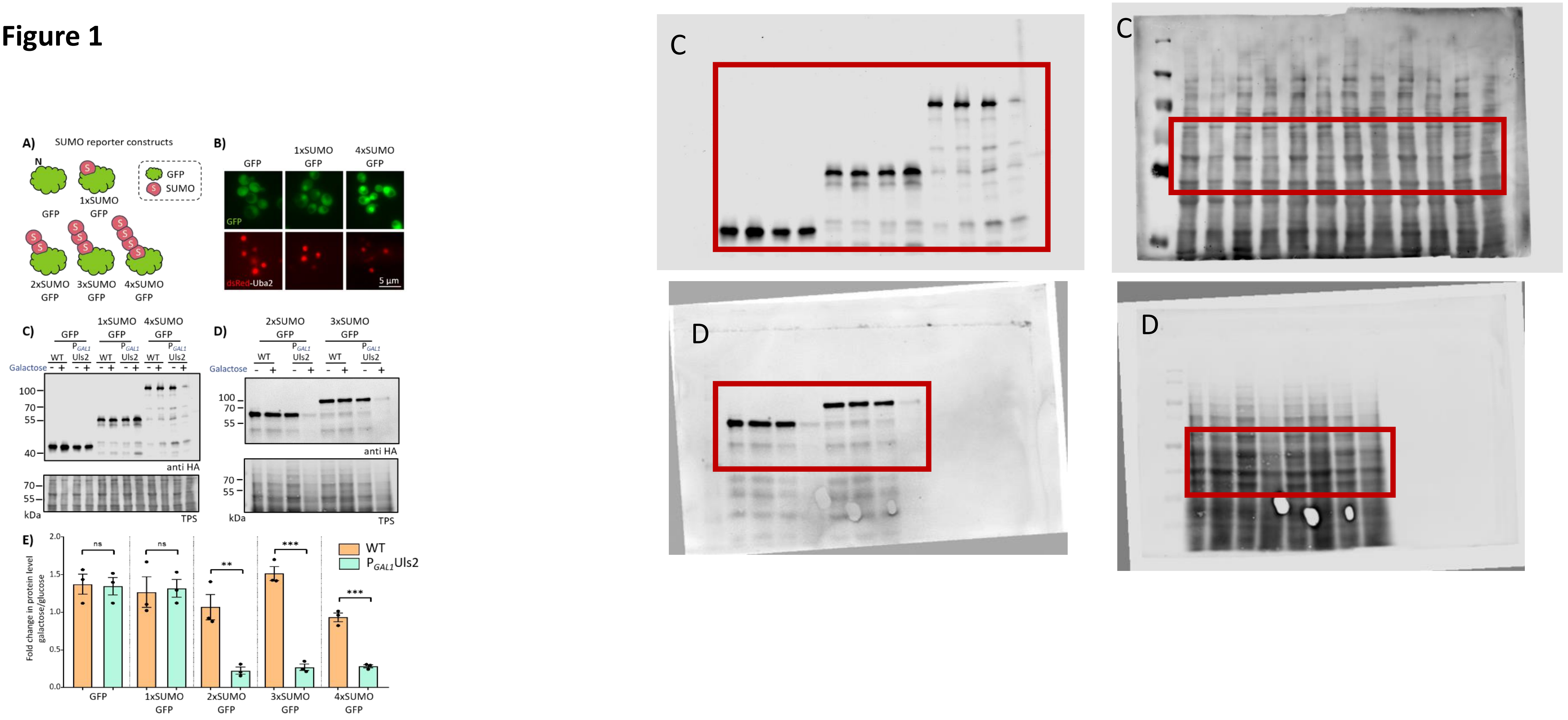

A) SUMO reporter constructs
N
GFP
SUMO
GFP
1xSUMO GFP
2xSUMO GFP
3xSUMO GFP
4xSUMO GFP
B)
GFP
1xSUMO GFP
4xSUMO GFP
dsRed-Uba2
5 µm
C)
Galactose
WT
Uls2
anti HA
TPS
kDa
D)
2xSUMO GFP
3xSUMO GFP
E)
Fold change in protein level galactose/glucose
ns
**
***
WT
C
D

Figure 2

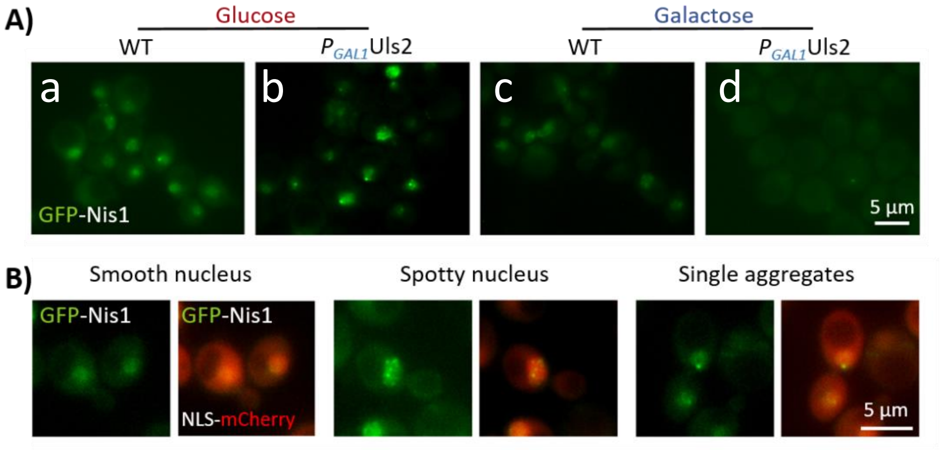

A)
Glucose
Galactose
WT
P_{GAL1}Uls2
WT
P_{GAL1}Uls2
a
b
c
d
GFP-Nis1
5 µm
B)
Smooth nucleus
Spotty nucleus
Single aggregates
GFP-Nis1
GFP-Nis1
NLS-mCherry
5 µm

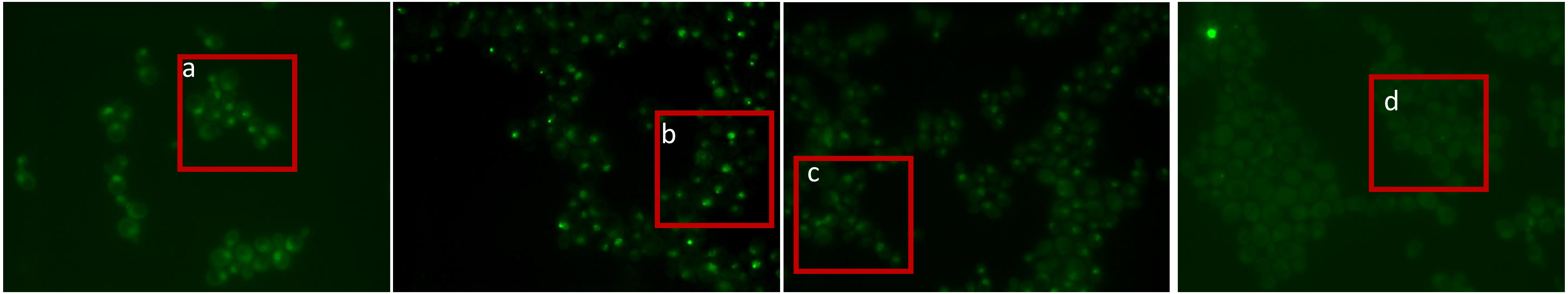

a
b
c
d

Figure 2

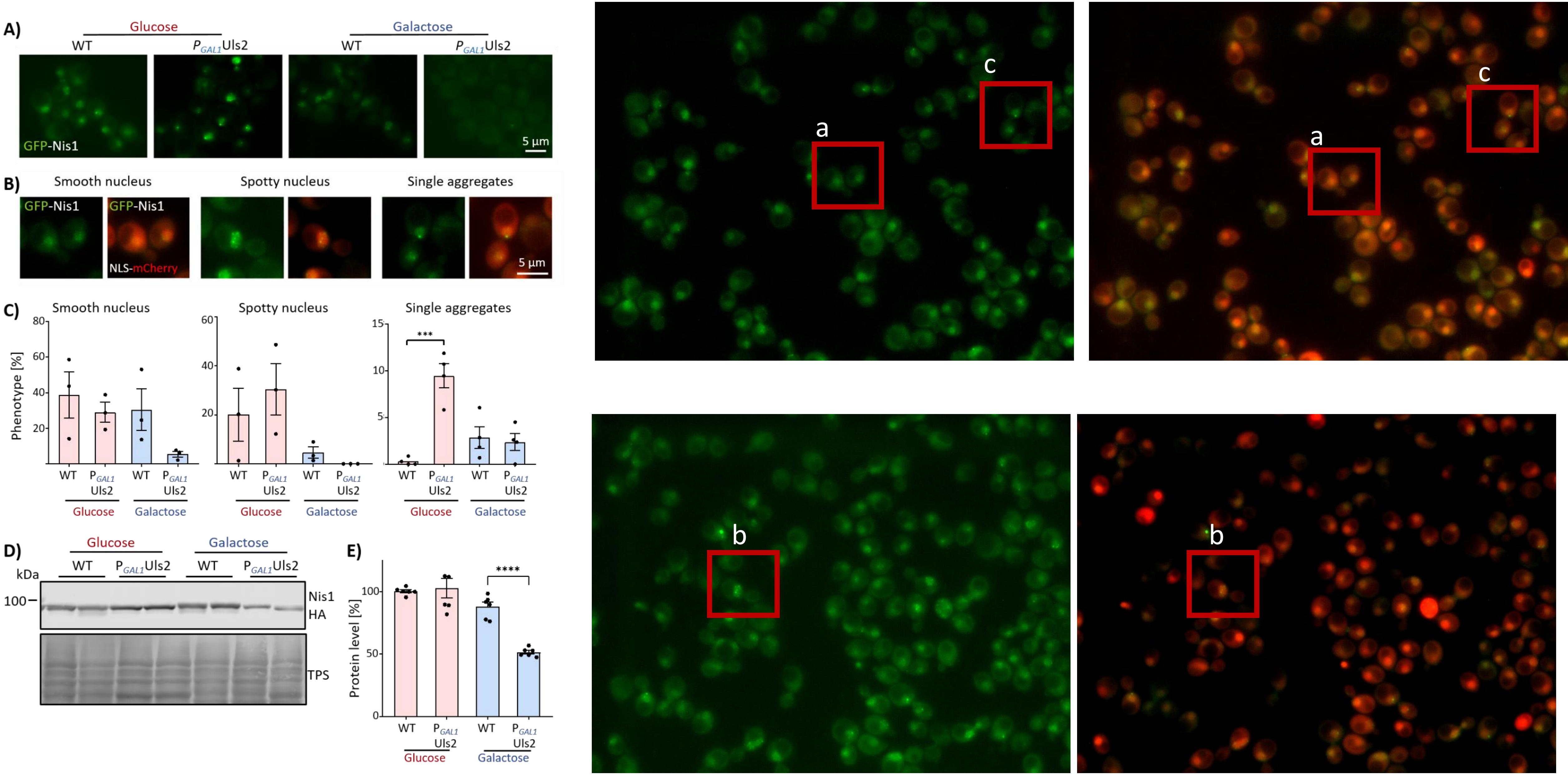
A)
Glucose
Galactose
WT
PGAL1Uls2
GFP-Nis1
5 µm
B)
Smooth nucleus
Spotty nucleus
Single aggregates
NLS-mCherry
C)
Phenotype [%]
***
D)
kDa
100
Nis1
HA
TPS
E)
Protein level [%]
****
a
b
c

Figure 2

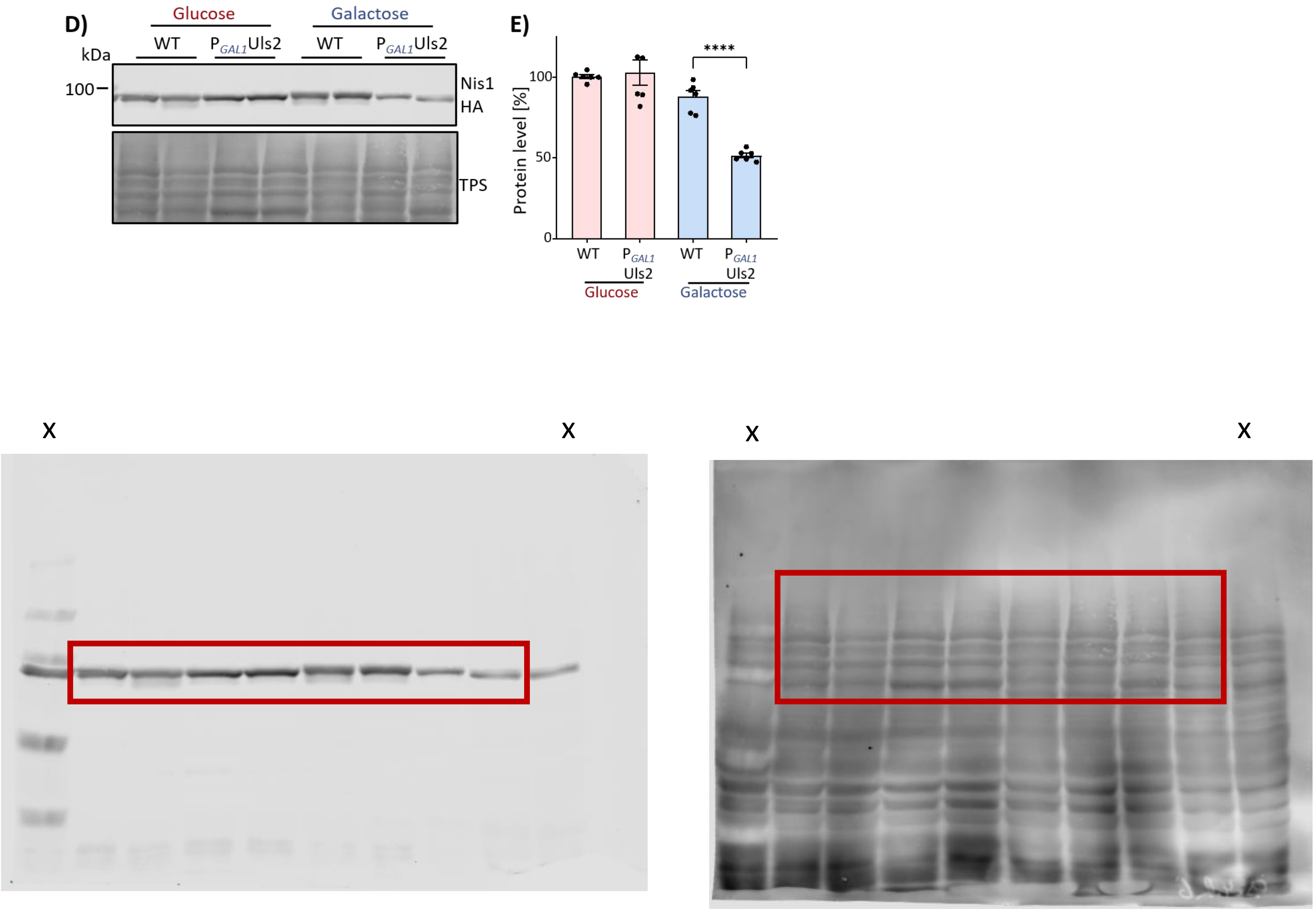
D)
Glucose
Galactose
WT
PGAL1Uls2
WT
PGAL1Uls2
kDa
100
Nis1
HA
TPS
E)
****
Protein level [%]
100
50
0
WT
PGAL1 Uls2
WT
PGAL1 Uls2
Glucose
Galactose
X
X
X
X

Figure 3

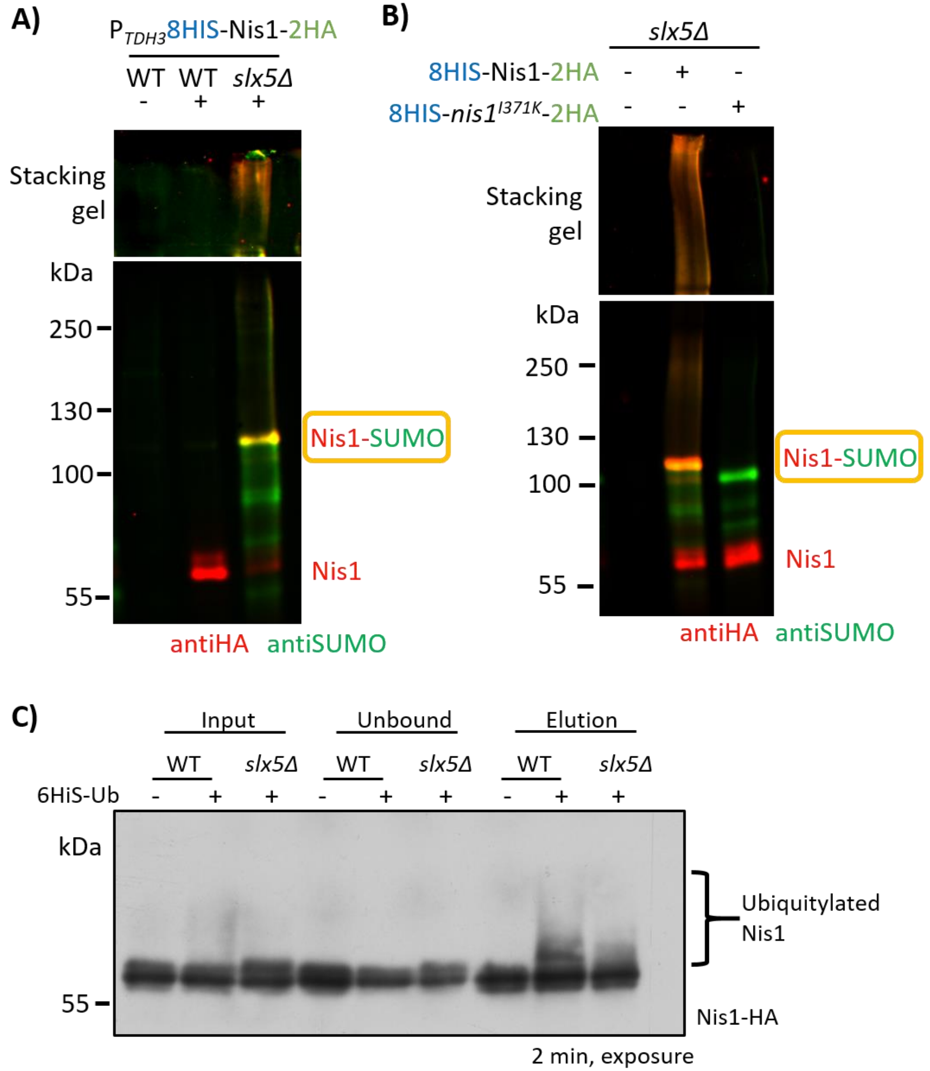

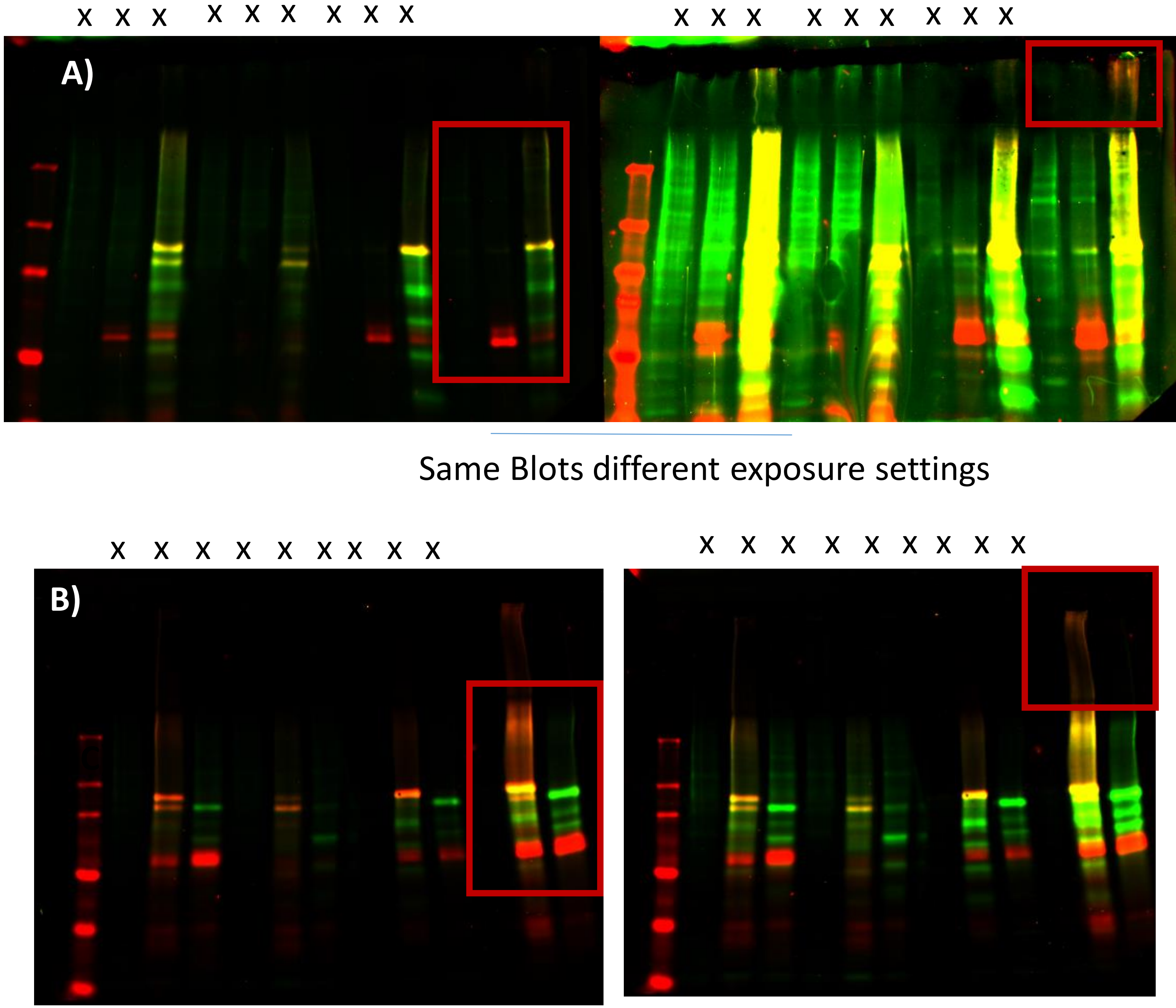

Same Blots different exposure settings

Same Blots different exposure settings

Figure 3

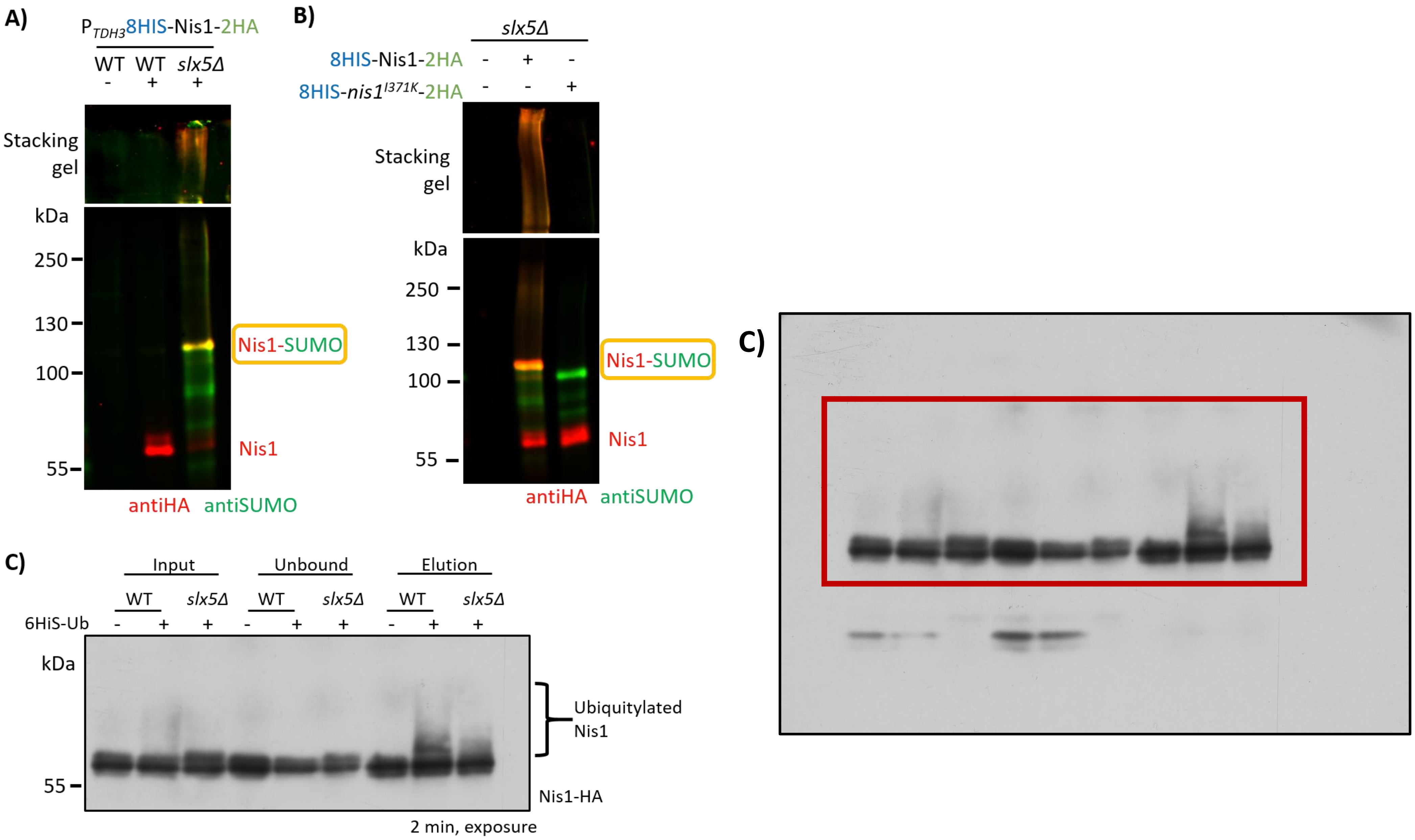

A)
P TDH3 8HIS-Nis1-2HA
WT WT slx5Δ
- + +
Stacking gel
kDa
250
130
100
55
Nis1-SUMO
Nis1
antiHA antiSUMO
B)
slx5Δ
8HIS-Nis1-2HA - + -
8HIS-nis1 I371K-2HA - - +
Stacking gel
kDa
250
130
100
55
Nis1-SUMO
Nis1
antiHA antiSUMO
C)
Input Unbound Elution
WT slx5Δ WT slx5Δ WT slx5Δ
6HiS-Ub - + + - + + - + +
kDa
55
Ubiquitylated Nis1
Nis1-HA
2 min, exposure
C)

Figure 4 and Figure S7

Figure 4

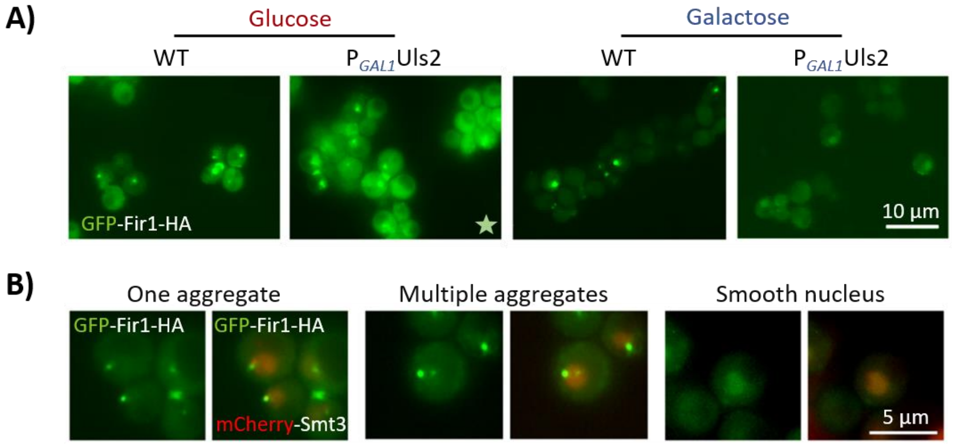

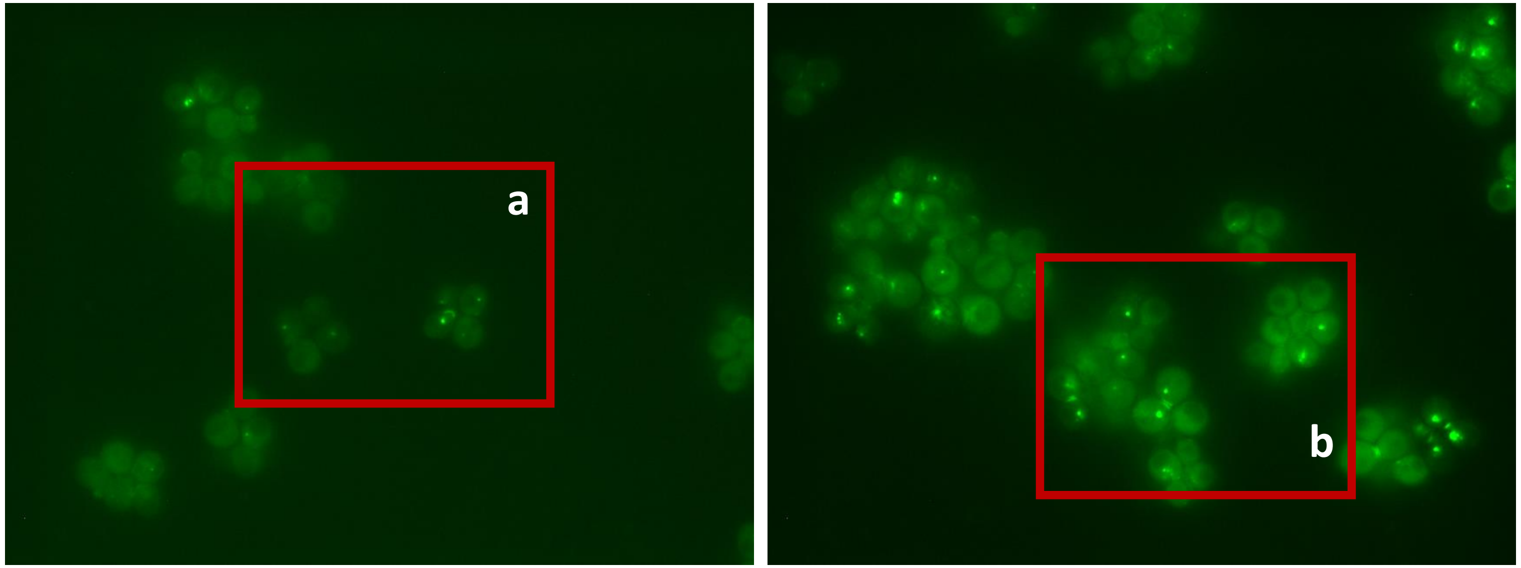

Figure S7

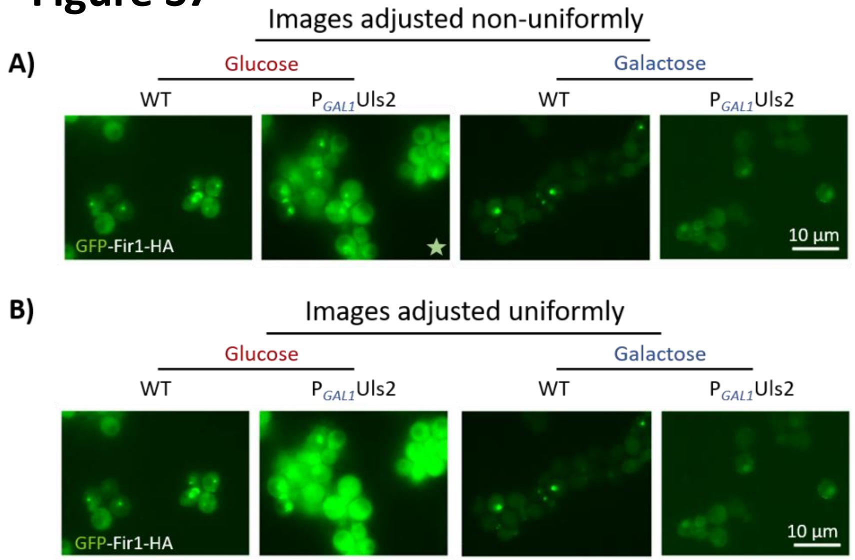

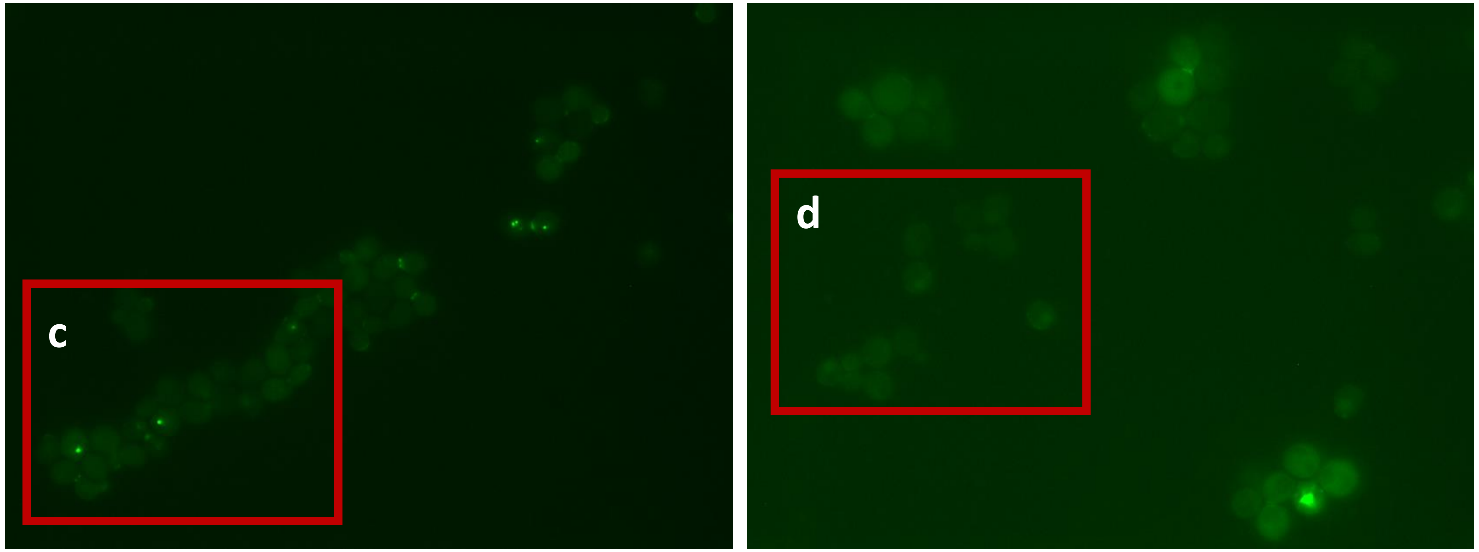

Figure 4

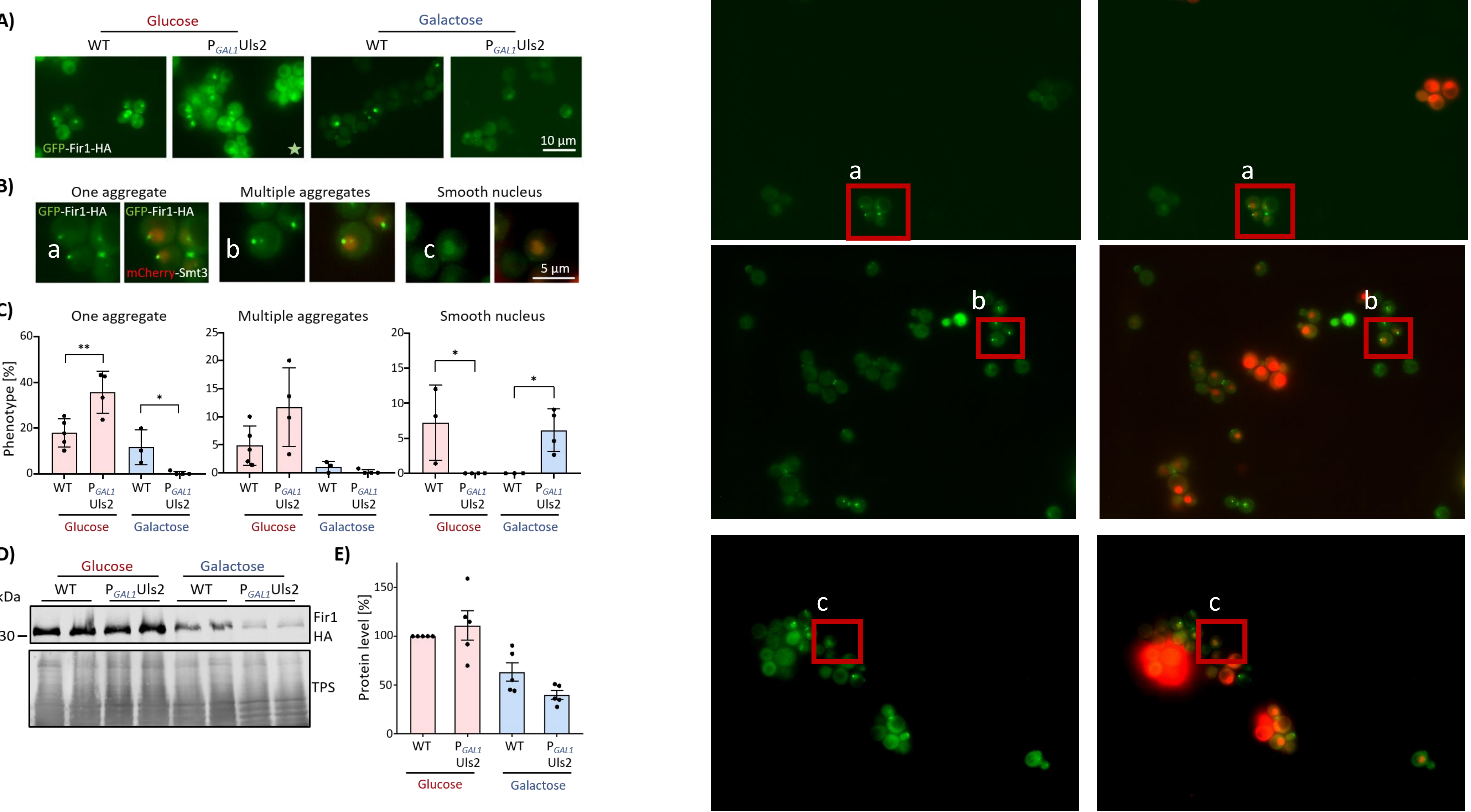
A)
Glucose
Galactose
WT
P GAL1 Uls2
WT
P GAL1 Uls2
GFP-Fir1-HA
10 µm
B)
One aggregate
Multiple aggregates
Smooth nucleus
GFP-Fir1-HA
GFP-Fir1-HA
mCherry-Smt3
a
b
c
5 µm
C)
One aggregate
Multiple aggregates
Smooth nucleus
Phenotype [%]
WT
P GAL1 Uls2
Glucose
Galactose
D)
kDa
30
Fir1
HA
TPS
E)
Protein level [%]
a
b
c

Figure 4

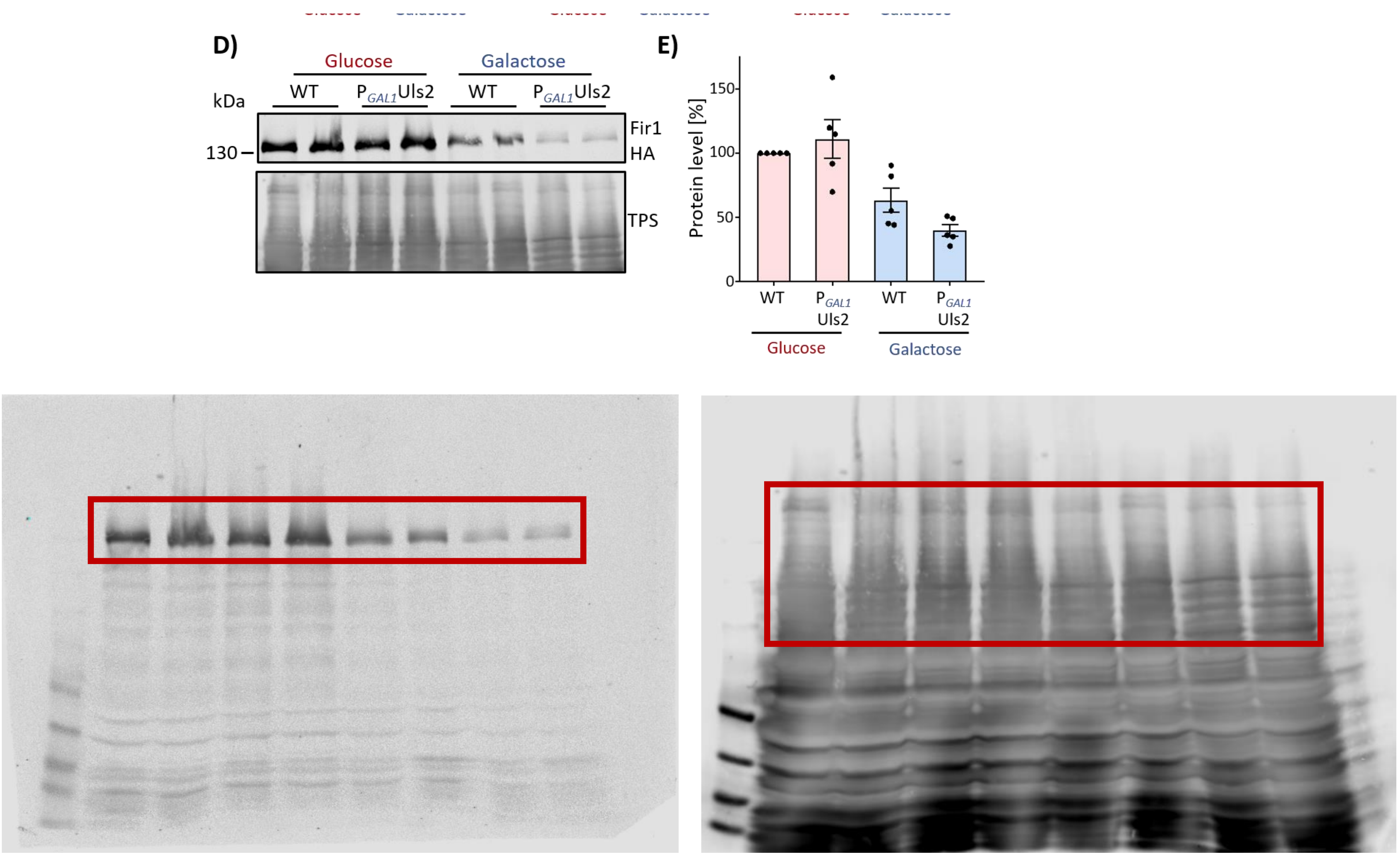

D)
Glucose
Galactose
WT
P GAL1 Uls2
WT
P GAL1 Uls2
kDa
130
Fir1
HA
TPS
E)
Protein level [%]
150
100
50
0
WT
P GAL1 Uls2
WT
P GAL1 Uls2
Glucose
Galactose

Figure 5

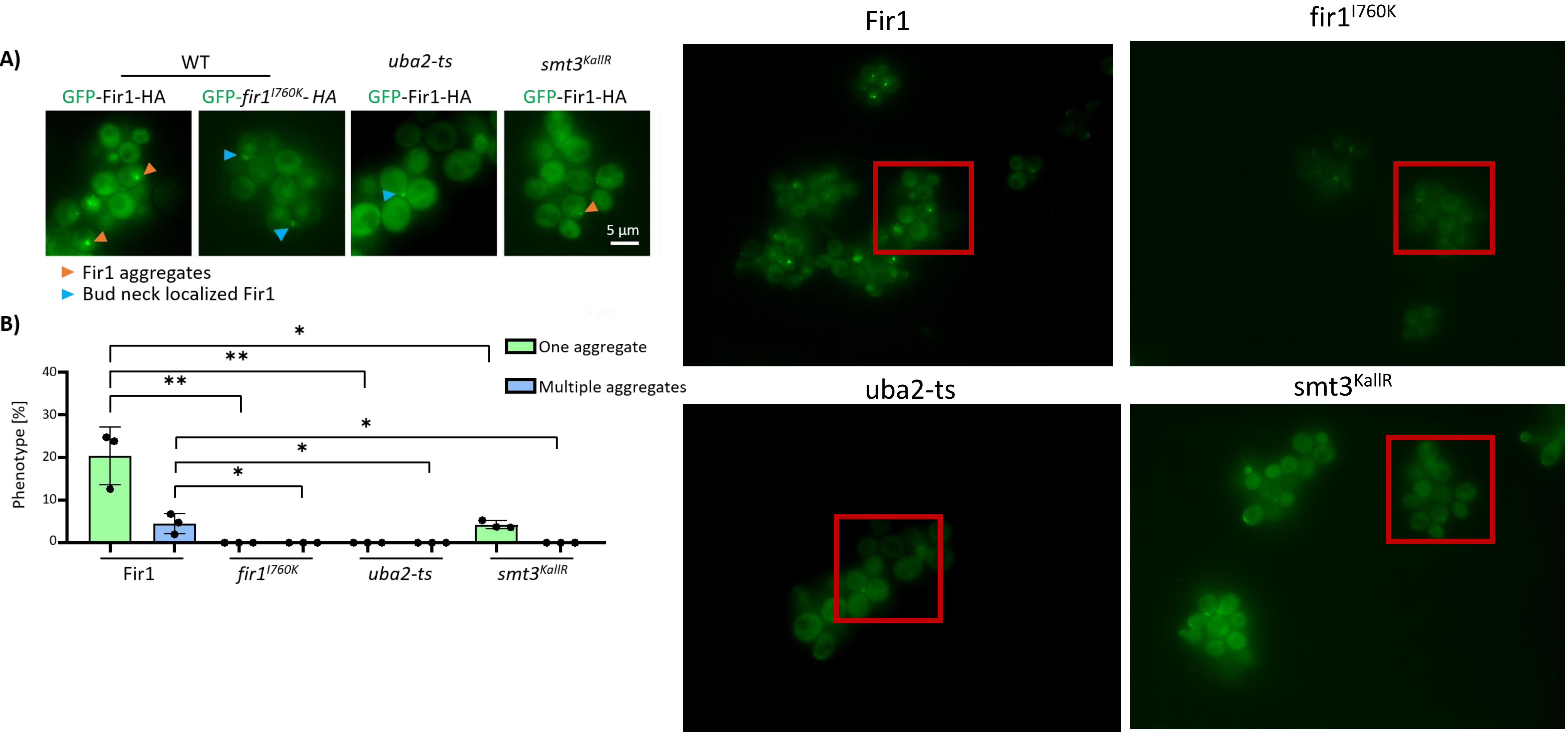

A)
WT
uba2-ts
smt3KallR
GFP-Fir1-HA
GFP-fir1I760K- HA
GFP-Fir1-HA
GFP-Fir1-HA
5 µm
Fir1 aggregates
Bud neck localized Fir1
B)
One aggregate
Multiple aggregates
Phenotype [%]
40
30
20
10
0
*
**
**
*
*
*
Fir1
fir1I760K
uba2-ts
smt3KallR
Fir1
fir1I760K
uba2-ts
smt3KallR

Figure 5

C)

GFP | mCherry | mCherry GFP | mCherry GFP

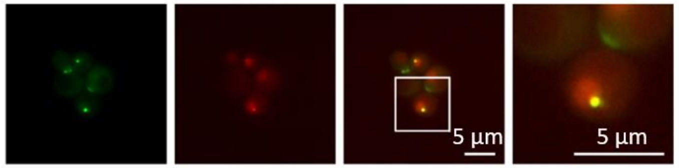

P$_{TDH3}$GFP-Fir1-HA

P$_{GAL1}$mCherry-Smt3

D)

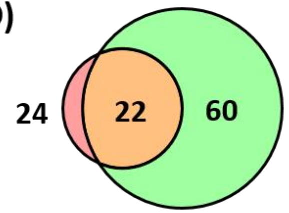

Total spots out of 316 cells

SUMO aggregates

colocalized aggregates

Fir1 aggregates

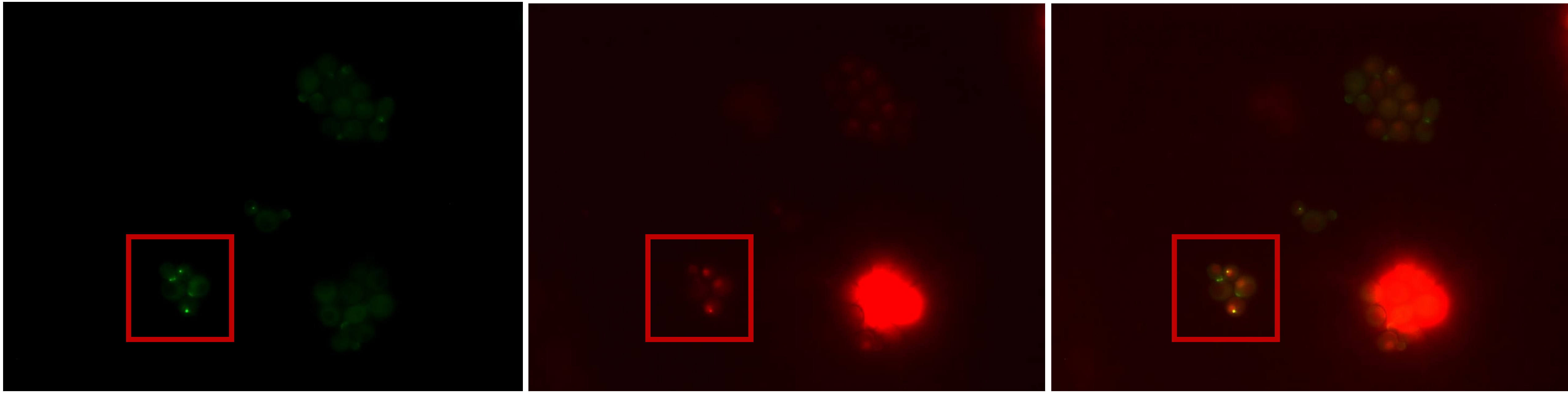

Figure 6 and Figure S8

Figure 6

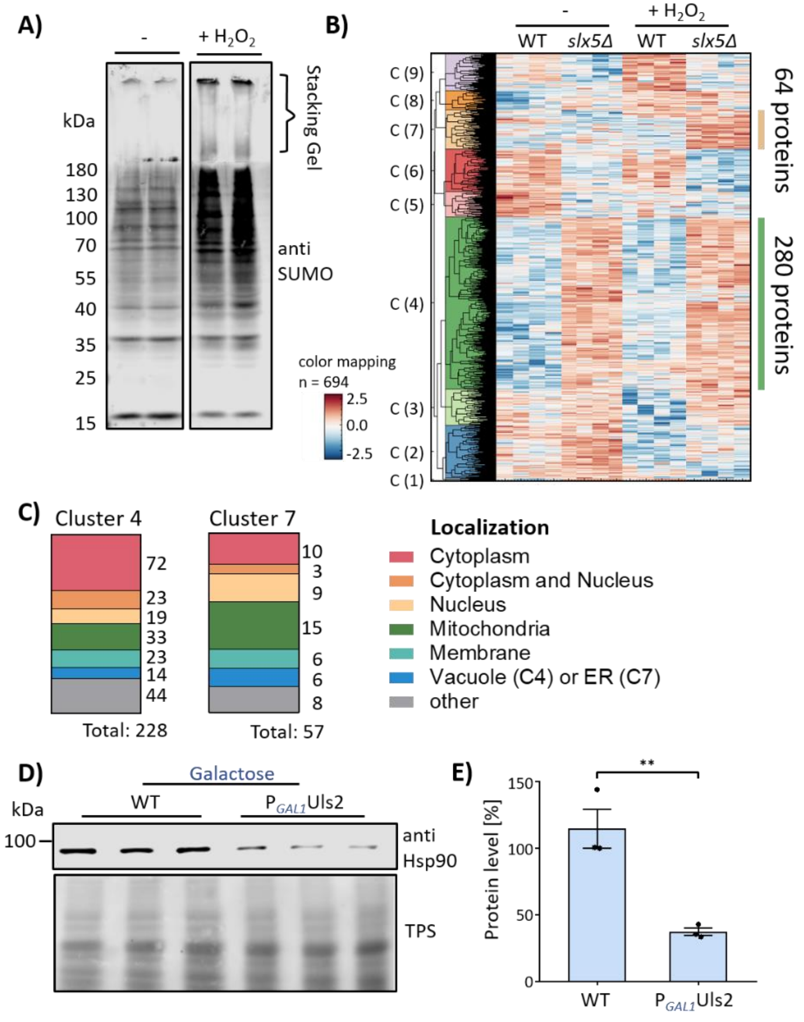

Figure S8

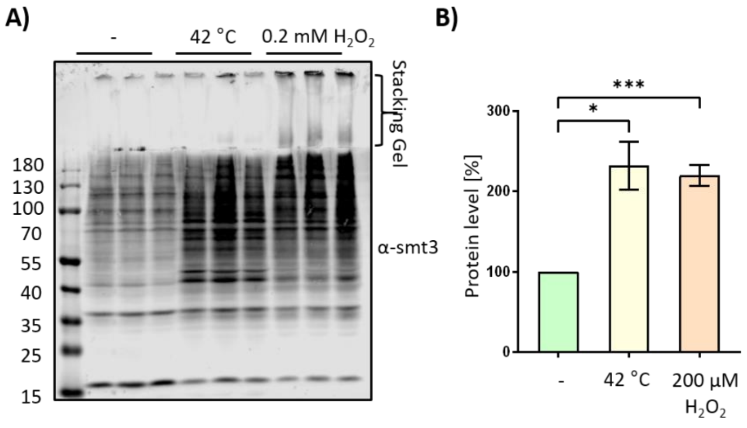

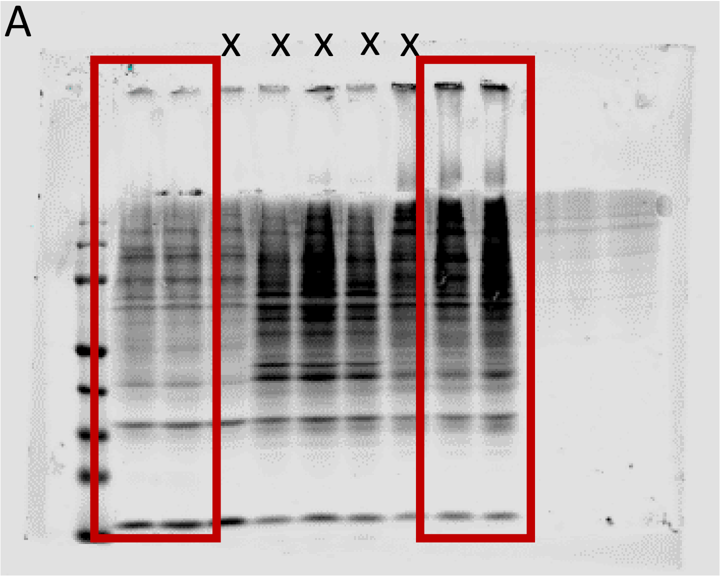

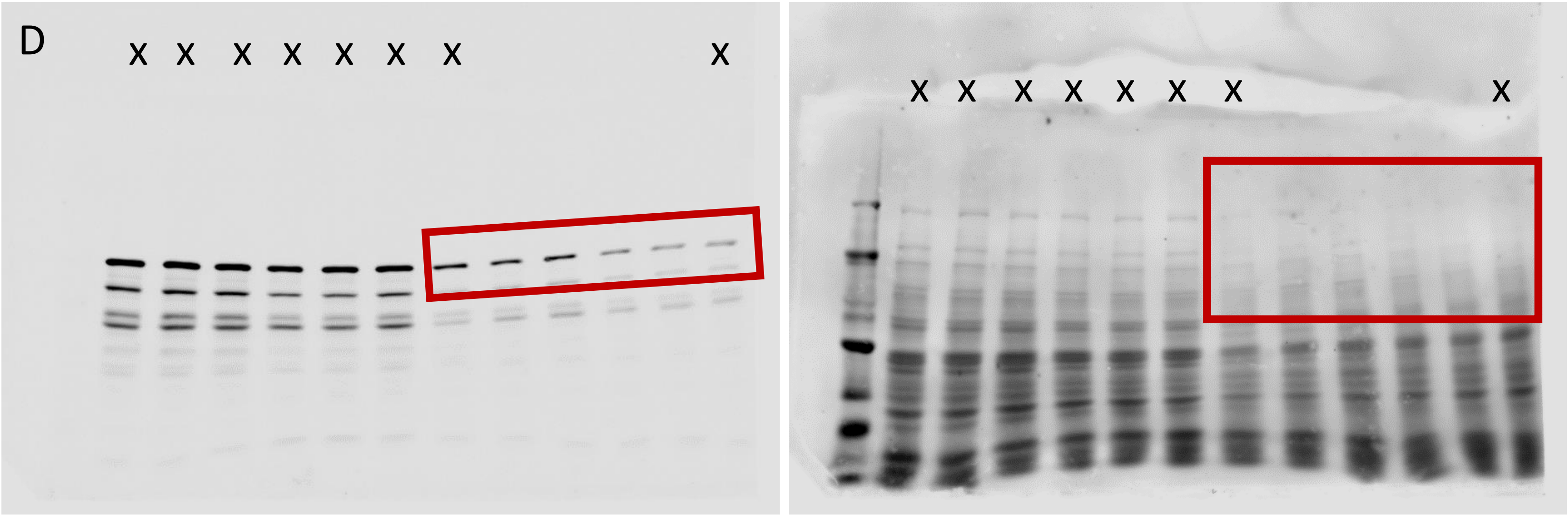

Figure 6

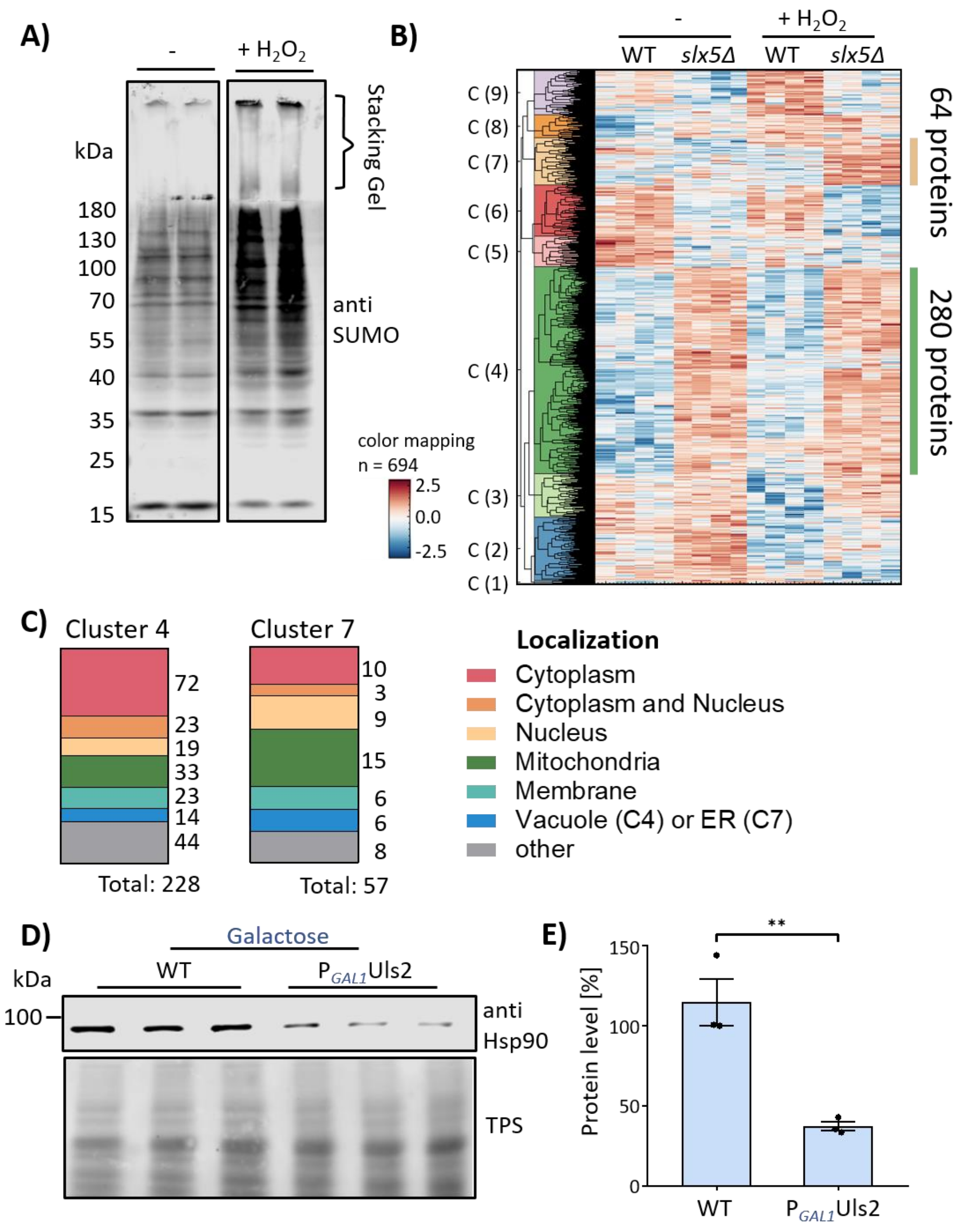

Columns in hCluster were switched
According for more appropriate order

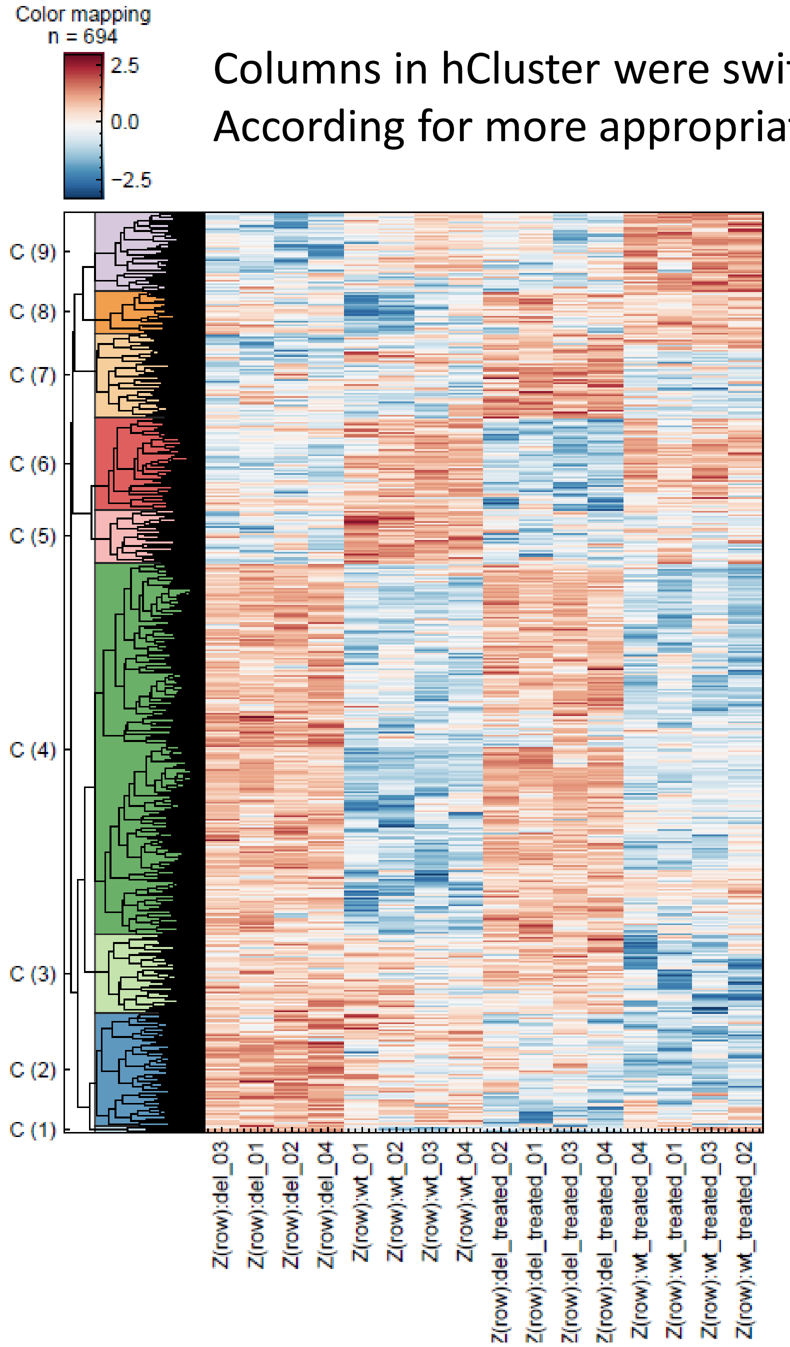

Figure S1

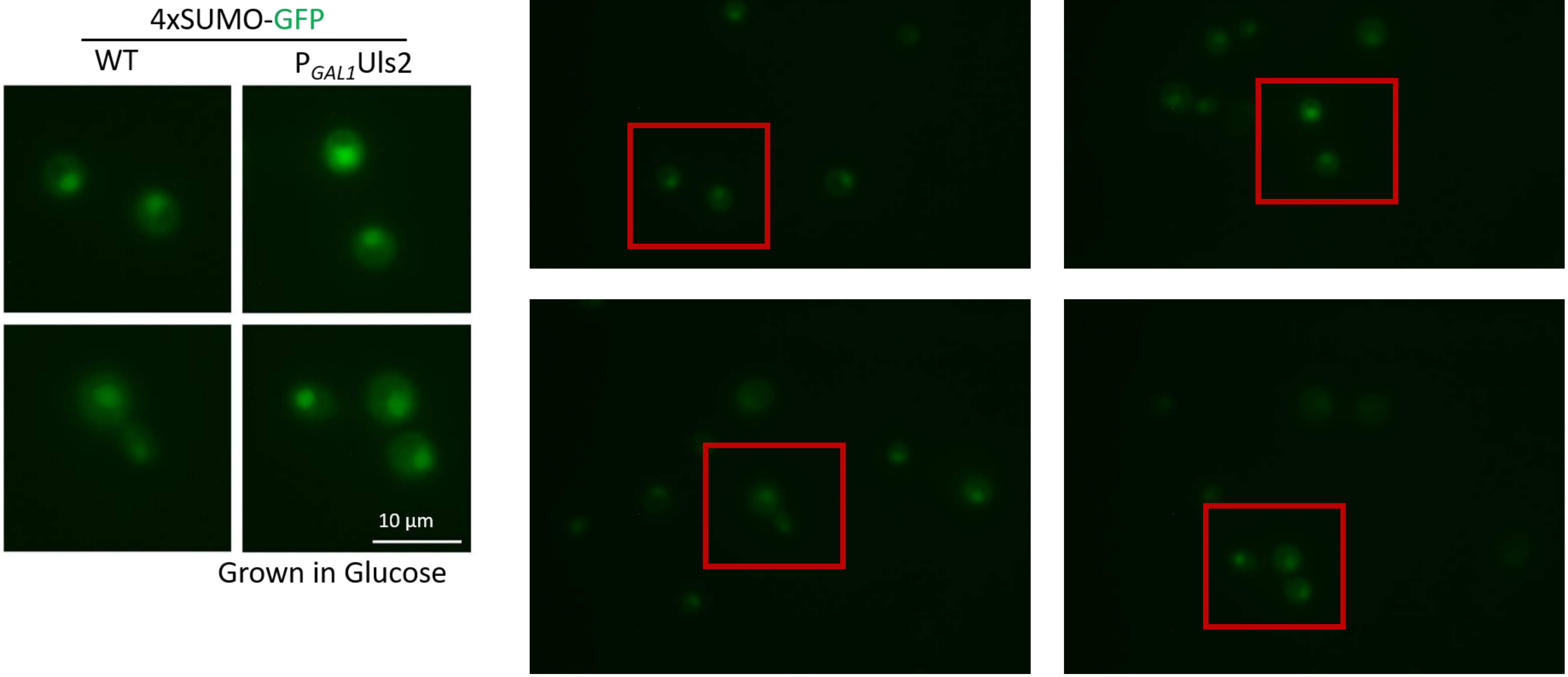

Figure S2

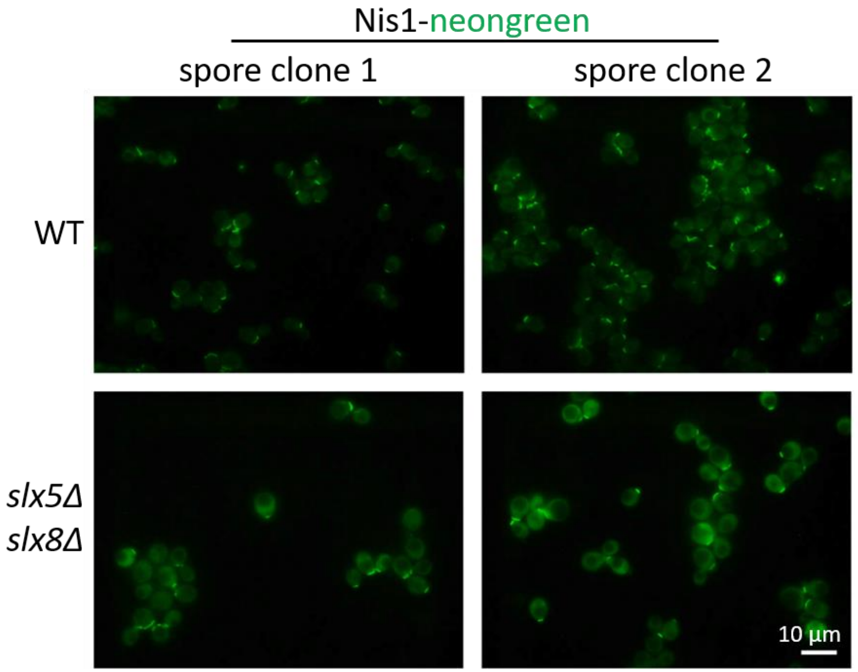
Nis1-neongreen
spore clone 1
spore clone 2
WT
slx5Δ
slx8Δ
10 µm

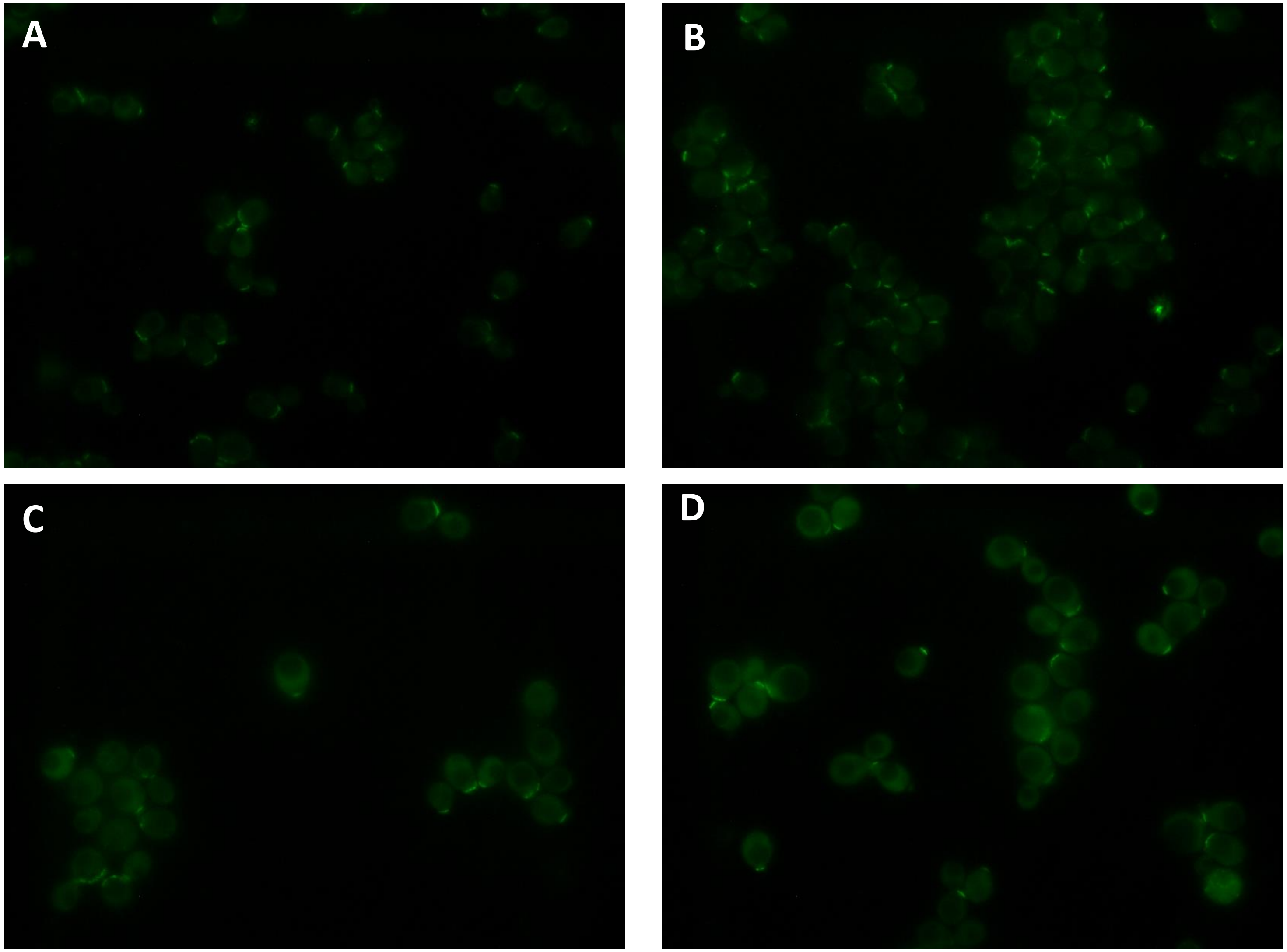
A
B
C
D

Figure S4

A)

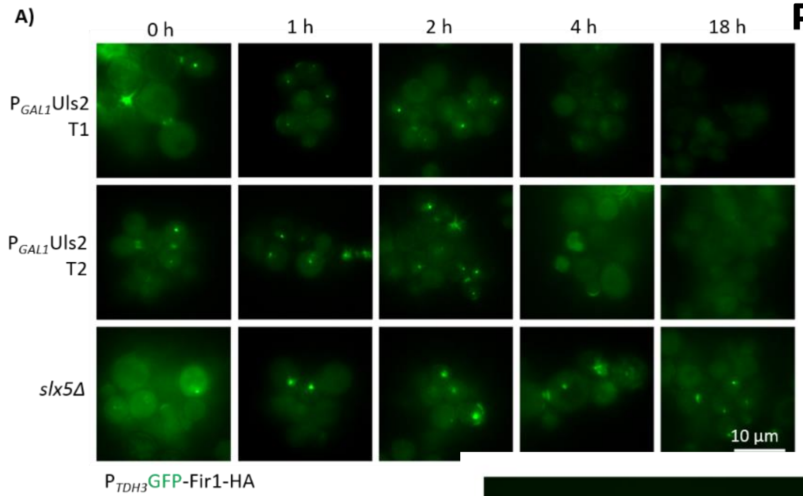
0 h
1 h
2 h
4 h
18 h
P$_{GAL1}$Uls2 T1
P$_{GAL1}$Uls2 T2
slx5Δ
10 µm
P$_{TDH3}$GFP-Fir1-HA

B)

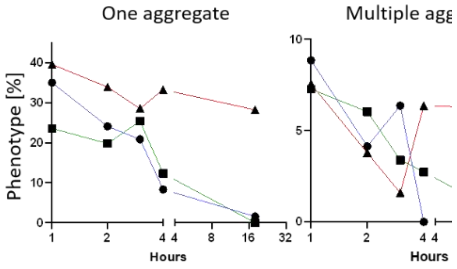
One aggregate
Multiple agg
Phenotype [%]
Hours
Hours

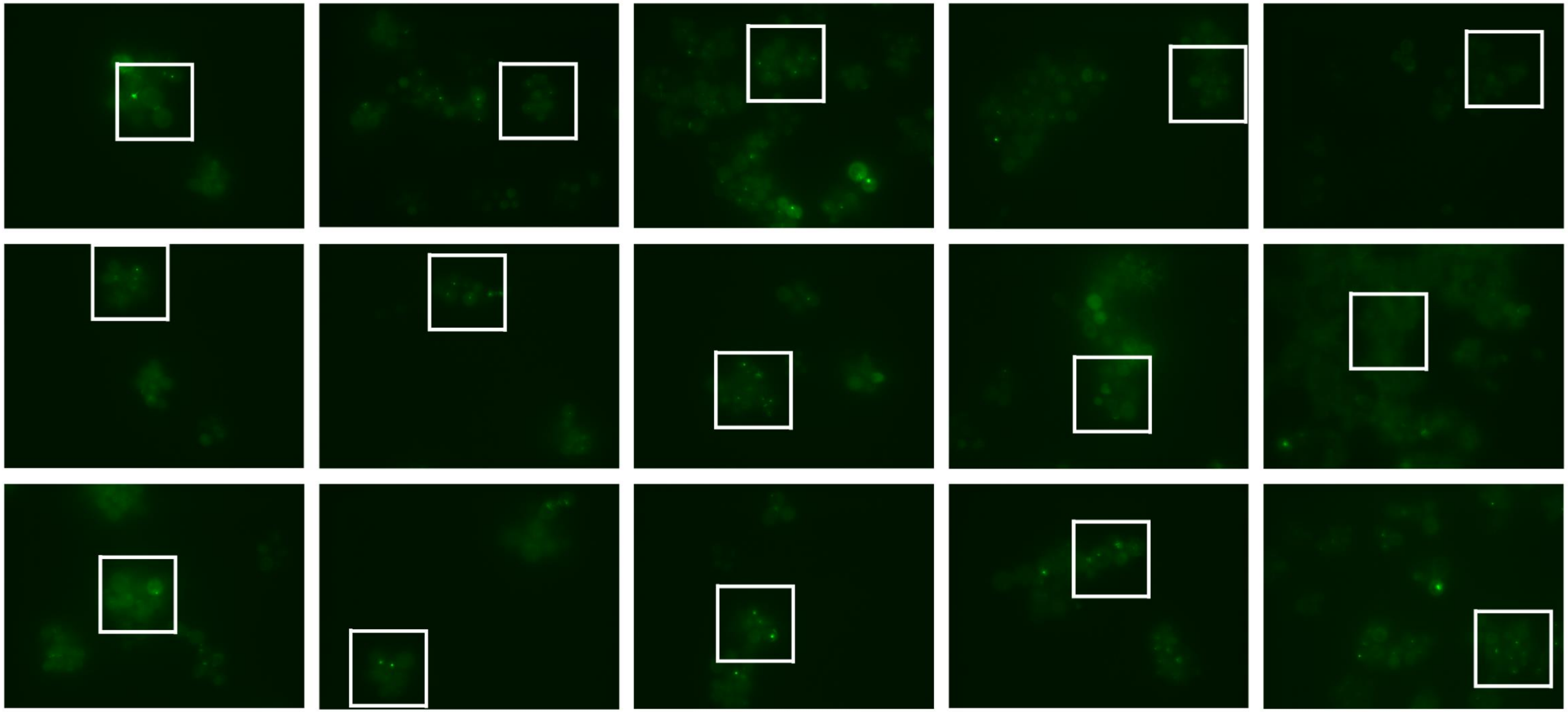

Figure S5

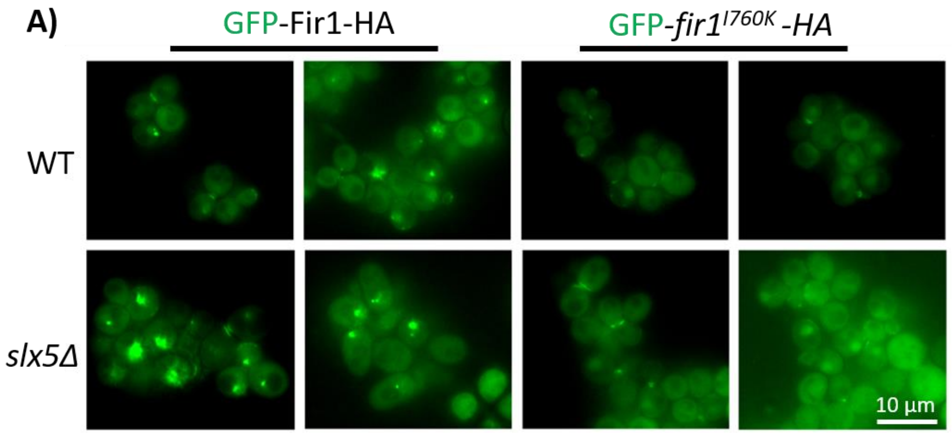

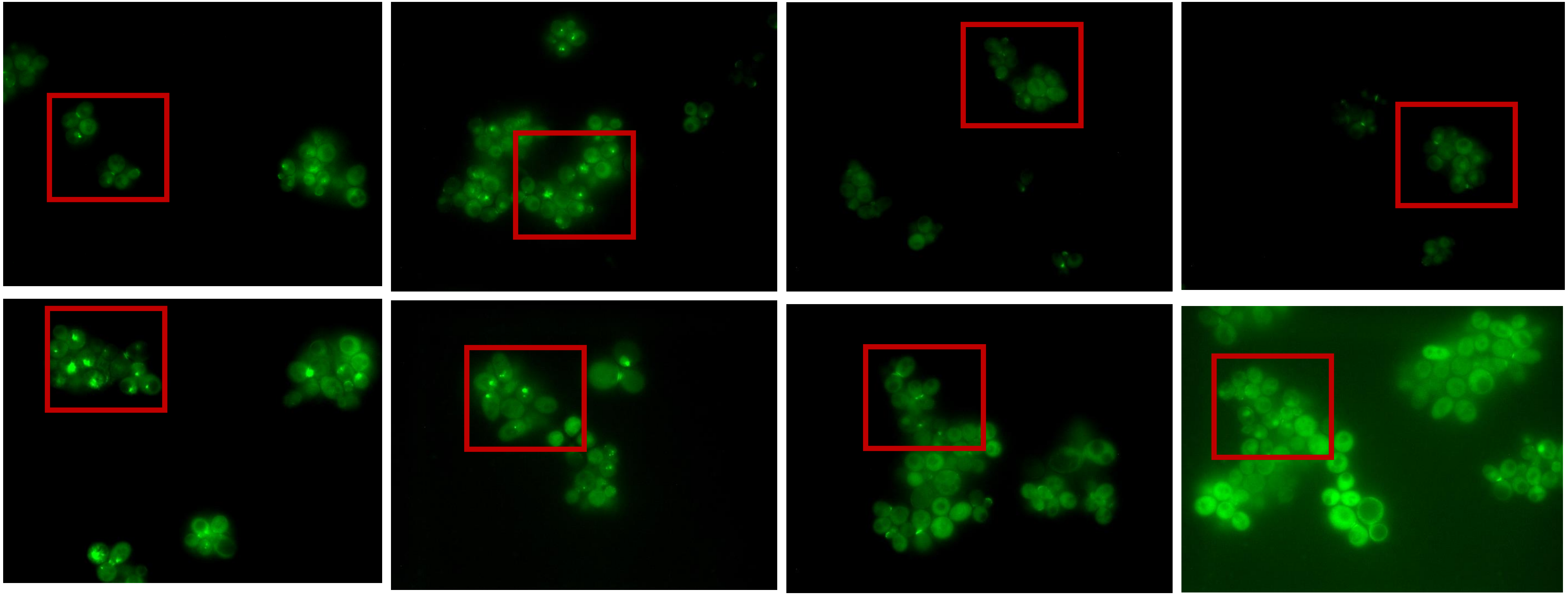

Figure S6

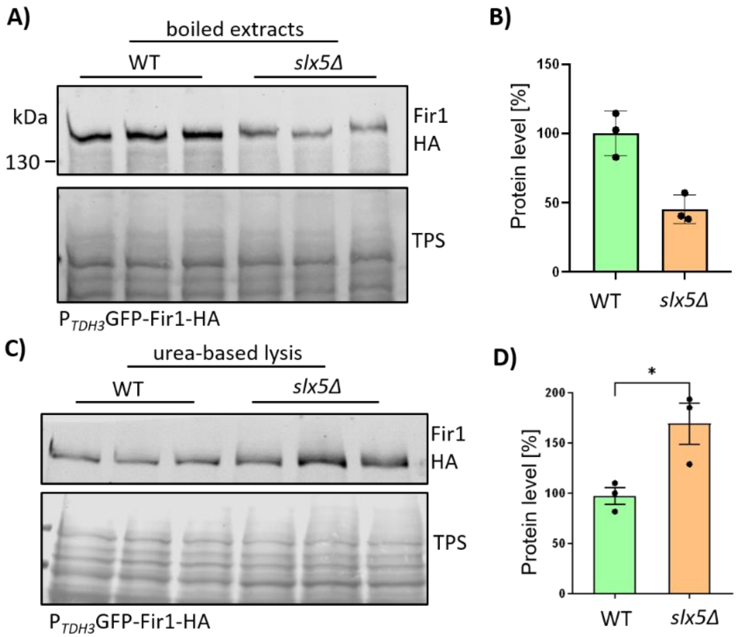
A)
boiled extracts
WT
slx5Δ
kDa
130
Fir1
HA
TPS
P TDH3 GFP-Fir1-HA
B)
Protein level [%]
150
100
50
0
WT
slx5Δ
C)
urea-based lysis
WT
slx5Δ
Fir1
HA
TPS
P TDH3 GFP-Fir1-HA
D)
*
Protein level [%]
200
150
100
50
0
WT
slx5Δ

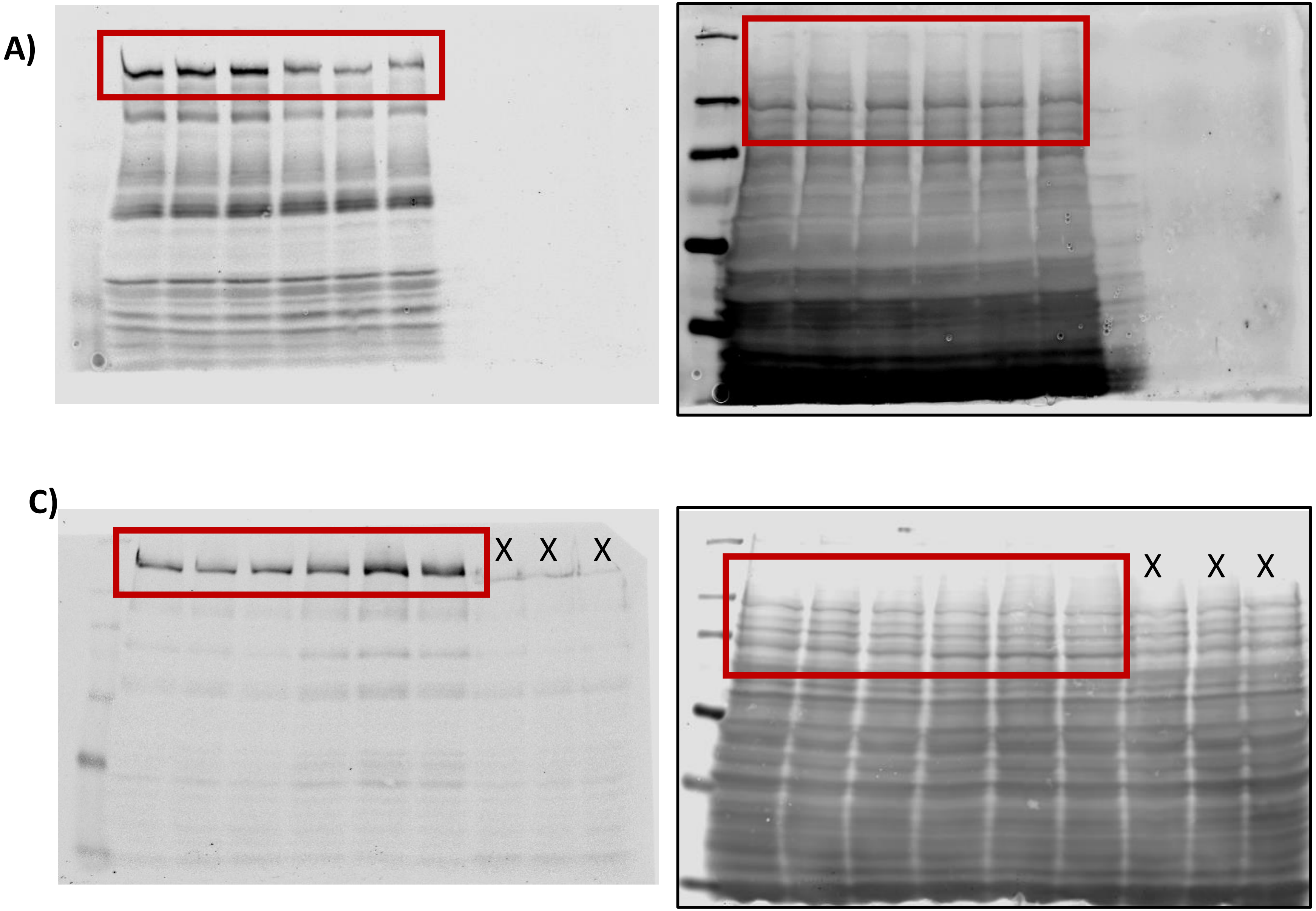
A)
C)
X
X
X
X
X
X

Figure S9

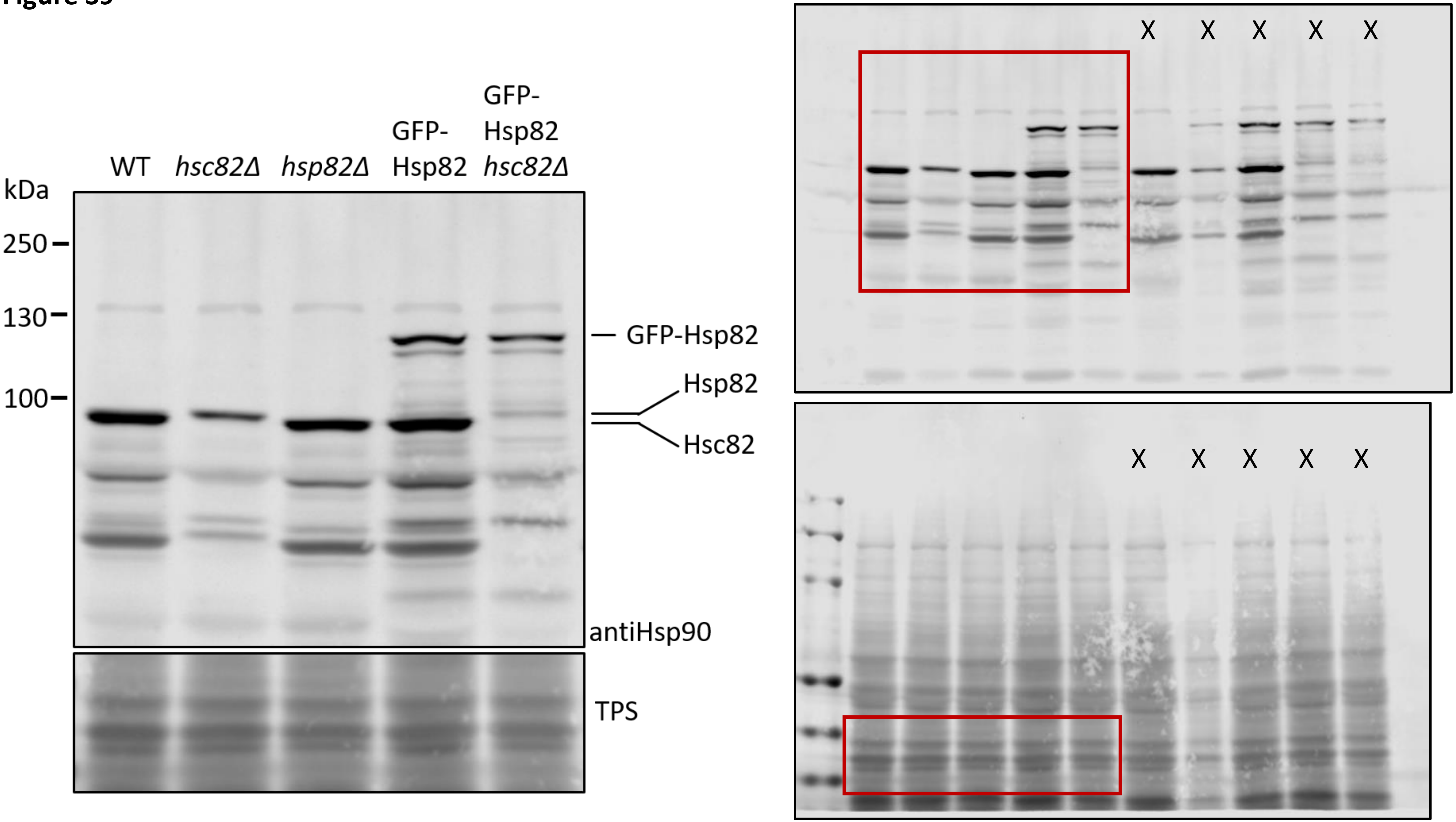
WT
hsc82Δ
hsp82Δ
GFP-Hsp82
GFP-Hsp82 hsc82Δ
kDa
250
130
100
GFP-Hsp82
Hsp82
Hsc82
antiHsp90
TPS
X
X
X
X
X
X
X
X
X
X
