# Supplementary material for: Role of Nuclear SUMO-Targeted Ubiquitin Ligase Uls2 (Slx5-Slx8) in Quality Control of Cytosolic Proteins
Source: Biomolecules. 2026 Sep 11;16(9):1322. doi: 10.3390/biom16091322 (PMC13604690; doi:10.3390/biom16091322)
Supplement: Supplementary file 1 [file biomolecules-16-01322-s001.zip › Profe_Austermann_Supplementary Figures.pdf]

Supplementary Material

Table S1. Yeast strains used in this study.

**Strain name**	**Relevant Genotype**
**JD47-13C**	*ADE2 leu2-3,112 lys2-801 his3Δ200 trp1Δ63 ura3-52* (*MATa)*
**YKU146**	*slx5Δ*::*TRP1* (*MATalpha)*
**YLA13**	P_{TDH3}*GFP-NIS1::LEU* (*MATa)*
**YLA17**	P_{TDH3}*GFP-NIS1::LEU2* P_{GAL1} *SLX5::kanMX4* P_{GAL1} *SLX8::HIS3* (*MATa*)
**YFP106**	*NIS1-6HA::TRP1*(*MATa*)
**YSP8**	*NIS1-6HA::TRP1 slx5Δ*::*TRP1* (*MATa*)
**YFP255**	P_{TDH3}*GFP-NIS1-HA::LEU2* (*MATa*)
**YFP256**	P_{TDH3}*GFP-NIS1-HA::LEU2* P_{GAL1} *SLX5::kanMX4* P_{GAL1} *SLX8::HIS3* (*MATa*)
**YFP257**	P_{TDH3}*8HIS-NIS1-HA::LEU2* (*MATa*)
**YFP258**	P_{TDH3}*8HIS-NIS1-HA::LEU2 slx5Δ::TRP1* (*MATalpha)*
**YFP259**	P_{TDH3}*8HIS-NIS1-HA::LEU2 slx5Δ::TRP1* (*MATalpha)*
**YFP102**	*NIS1-neongreen::nat*, *slx5Δ::TRP1 slx8Δ*::kanMX4 *MATa*
**YFP104**	*NIS1-neongreen::nat* (*MATa*)
**YFP167**	P_{TDH3}*yeGFP-FIR1-HA::LEU2* (*MATa*)
**YFP168**	P_{GAL1}*SLX5::KanMX* P_{GAL1}*SLX8::HIS3* P_{TDH3}yeGFP-FIR1-HA::LEU2 (*MATa*)
**YFP169**	P_{TDH3}*yeGFP-FIR1-HA::LEU2 slx5Δ*::*TRP1* (*MATalpha)*
**YFP172**	P_{TDH3}*yeGFP-fir1-I760K-HA::LEU2* (*MATa*)
**YFP175**	P_{TDH3}*yeGFP-fir1-I760K-HA::LEU2 slx5Δ*::*TRP1* (*MATalpha)*
**YFP237**	$smt3^{KallR}$*::KanMX* P_{TDH3}*yeGFP-FIR1-HA::LEU2* (*MATa*)
**YFP238**	*uba2-ts* P_{TDH3}*yeGFP-FIR1-HA::LEU2* (*MATa*)
**YLD19**	P_{GAL1}*SLX5:TRP1*, P_{GAL1}*SLX8:HIS3*
**YLD27-1**	P_{GAL1}*SLX5:TRP1*, P_{GAL1}*SLX8:HIS3*, *pdr5Δ:hphNT1*,P_{CUP1}-*smt3(ΔG98-Y101)-smt3(ΔM1-Q8, Delta G98-Y101)- GFP-2xHA-Tcyc1:LEU2* (*MATa*)
**YGA25**	*hsc82Δ::KanMX pdr5Δ::hphNT1* (*MATa)*
**YGA26**	*hsp82Δ::KanMX pdr5Δ::hphNT1* (*MATa)*
**YJM271**	*Fir1-GFP Myo1-Cherry*
**YAD4181**	*cdh1::hphNT1 Fir1-GFP Myo1-Cherry*
**YAD4455**	*cdh1Δ::hphNT1 spa2Δ::kan Fir1::Fir1-GFP TRP1*

Table S2. Plasmids used in this study.

Name	Insert	Backbone
**YCplac33**	-	YCplac33 [32]
**pLA6**	P_{TDH3}*yeGFP-NIS1::LEU2*	pUC21 [31]
**pFP4**	P_{PDA1}-mCherry-NLS-Tcyc1::Leu2	pUC21 [31]
**pFP43**	P_{TDH3}*yeGFP-FIR1-HA*-T_{CYC1}	pUC21 [31]
**pFP45**	P_{TDH3}yeGFP-*fir1-I760K*-HA-T_{CYC1}	pUC21 [31]
**pFP68**	P_{TDH3}*8HIS-NIS1-HA*-T_{CYC1}	pUC21 [31]
**pFP69**	P_{TDH3}8HIS-*nis1-I391K*-HA-T_{CYC1}	pUC21 [31]
**pLD5**	P_{CUP1}*FLAG-His-GFP-2xHA*-T_{CYC1}	YIplac211 [32]
**pLD7**	P_{CUP1}*FLAG-His-1xSMT3-TEV-GFP-2xHA*-T_{CYC1}	YIplac211 [32]
**pLD9**	P_{CUP1}-*FLAG-His-1xSMT3-TEV-3xSMT3*$_{\Delta 1\text{-}8}$-*GFP-2xHA*-T_{CYC1}	YIplac211 [32]
**pLD11**	P_{CUP1}-*FLAG-His-1xSMT3-TEV-1xSMT3*$_{\Delta 1\text{-}8}$-*GFP-2xHA*-T_{CYC1}	YIplac211 [32]
**pLD13**	P_{CUP1}-*FLAG-His-1xSmt3-TEV 2xSmt3*$_{\Delta 1\text{-}8}$-*GFP-2xHA*-T_{CYC1}	YIplac211 [32]
**pKU103**	P_{GAL1}*6HIS-UBIQUITIN*:: *URA3*	YCplac33 [32]
**pMM86**	P_{GAL1}*mCherry-SMT3*:: *URA3*	pRS316 [33]
**pML108**	P_{CUP1}-*UBA2-dsRED*	YEplac181[32]

[31] Vieira, J.; Messing, J. New pUC-derived cloning vectors with different selectable markers and DNA replication origins. *Gene* **1991,** *100*, 189-194.

[32] Gietz, R. D.; A. Sugino, A. New yeast-Escherichia coli shuttle vectors constructed with in vitro mutagenized yeast genes lacking six-base pair restriction sites. *Gene* **1988,** *74*, 527-534.

[33] Sikorski, R.S.; Hieter, P. A system of shuttle vectors and yeast host strains designed for efficient manipulation of DNA in Saccharomyces cerevisiae. *Genetics* **1989**, 122, 19-27.

Table S3. Antibodies used in this study.

Target	Host	Dilution	Supplier
**αHA (3F10)**	rat, monoclonal	1:2.000	Sigma-Aldrich
**αHSP90 (5833)**	rabbit, polyclonal	1:5.000	Self made
**αSMT3**	rabbit, polyclonal	1:20.000	Self made
**αrat DyLight 800**	goat, polyclonal	1:5.000	Biotechnology
**αrat Alexa Fluor 680**	goat, polyclonal	1:5.000	Santa Cruz
**αrat HRP**	Goat, polyclonal	1:5.000	abcam
**αrabbit Alexa Fluor800+**	goat, polyclonal	1:5.000	ThermoFisher

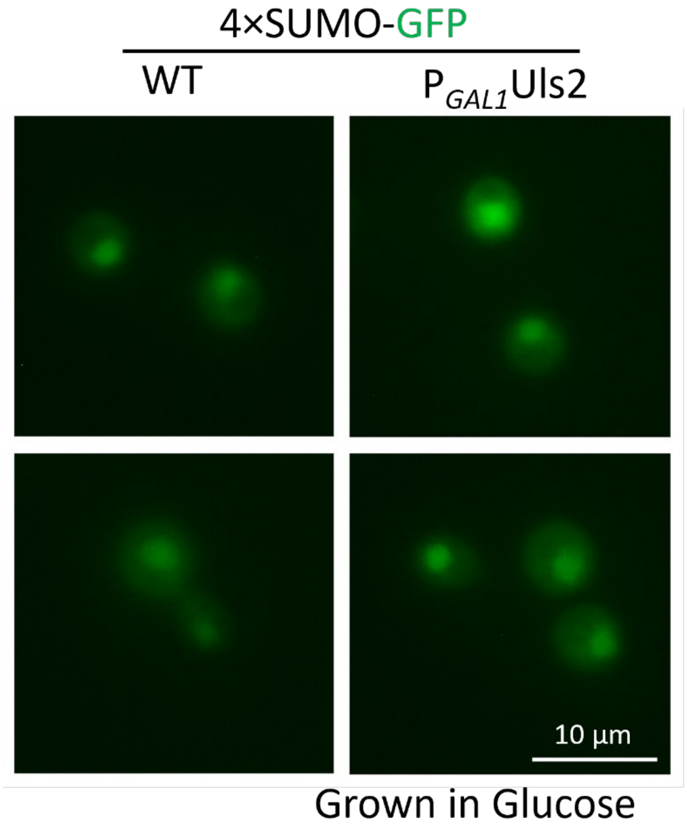

Figure S1. PolySUMO reporter localizes to the nucleus when *SLX5 and SLX8* are shut off. SUMO chain reporter is retained in the nucleus independent of Uls2. A *CEN* plasmid expressing GFP with 4×SUMO was introduced into a wild-type (WT) and a P_{GAL1}*SLX5* P_{GAL1}*SLX8* yeast strain. Cells of the obtained transformants were examined using life-cell fluorescent microscopy after growth in synthetic selective (S) media after growth for 8 h supplemented with 2% glucose resulting in the shut off of *SLX5* and *SLX8* transcription. Scale bar: 10 µM.

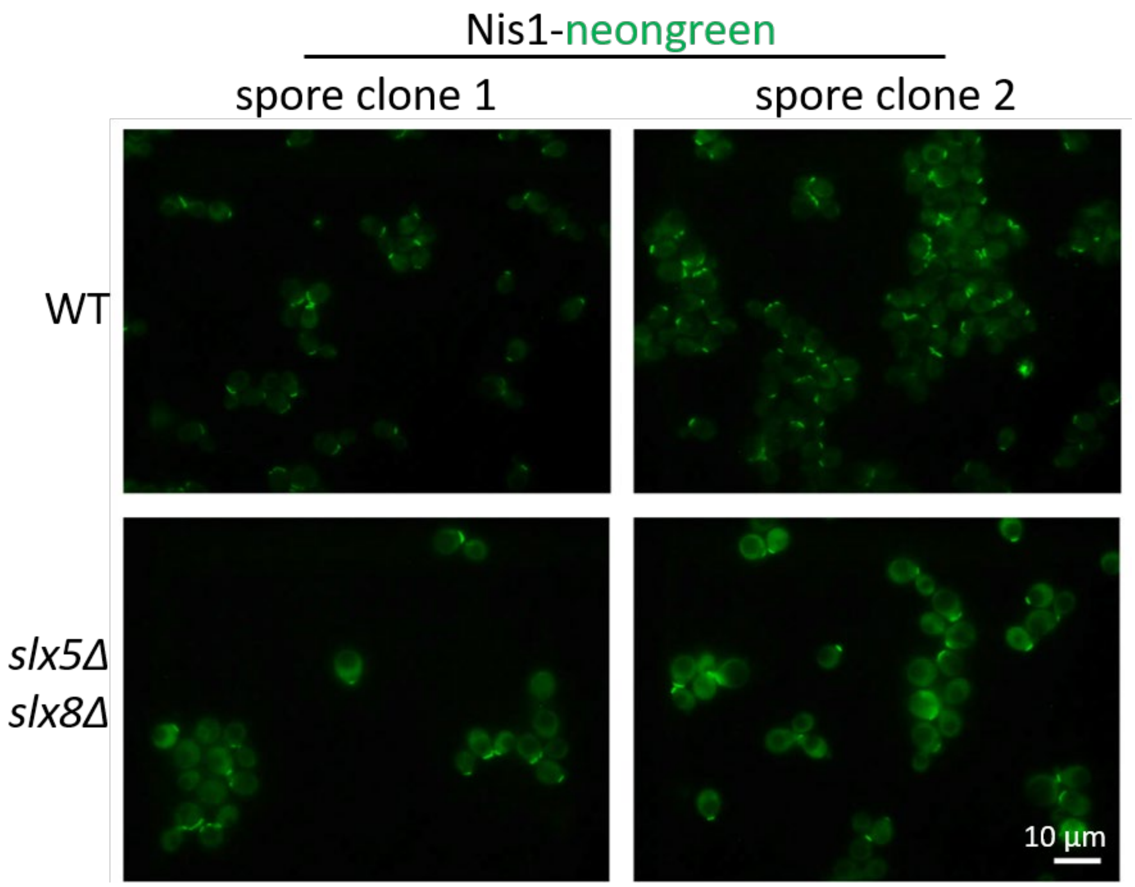

Figure S2. Endogenous Nis1 accumulates to higher levels in a *slx5Δ slx8Δ* strain. Nis1 was C-terminally tagged with neongreen and expressed from its native promoter at the endogenous gene locus. The tag was first introduced into the wild-type (WT) and subsequently into the *slx5Δ slx8Δ* background by crossing and tetrad dissection. Live cells were examined using fluorescence microscopy after growth in YP glucose. The experiments were performed with two independent spore clones each for WT and *slx5Δ slx8Δ*. All images were adjusted with +60% brightness and +20% contrast using PowerPoint (Microsoft). Scale bar: 10 µM.

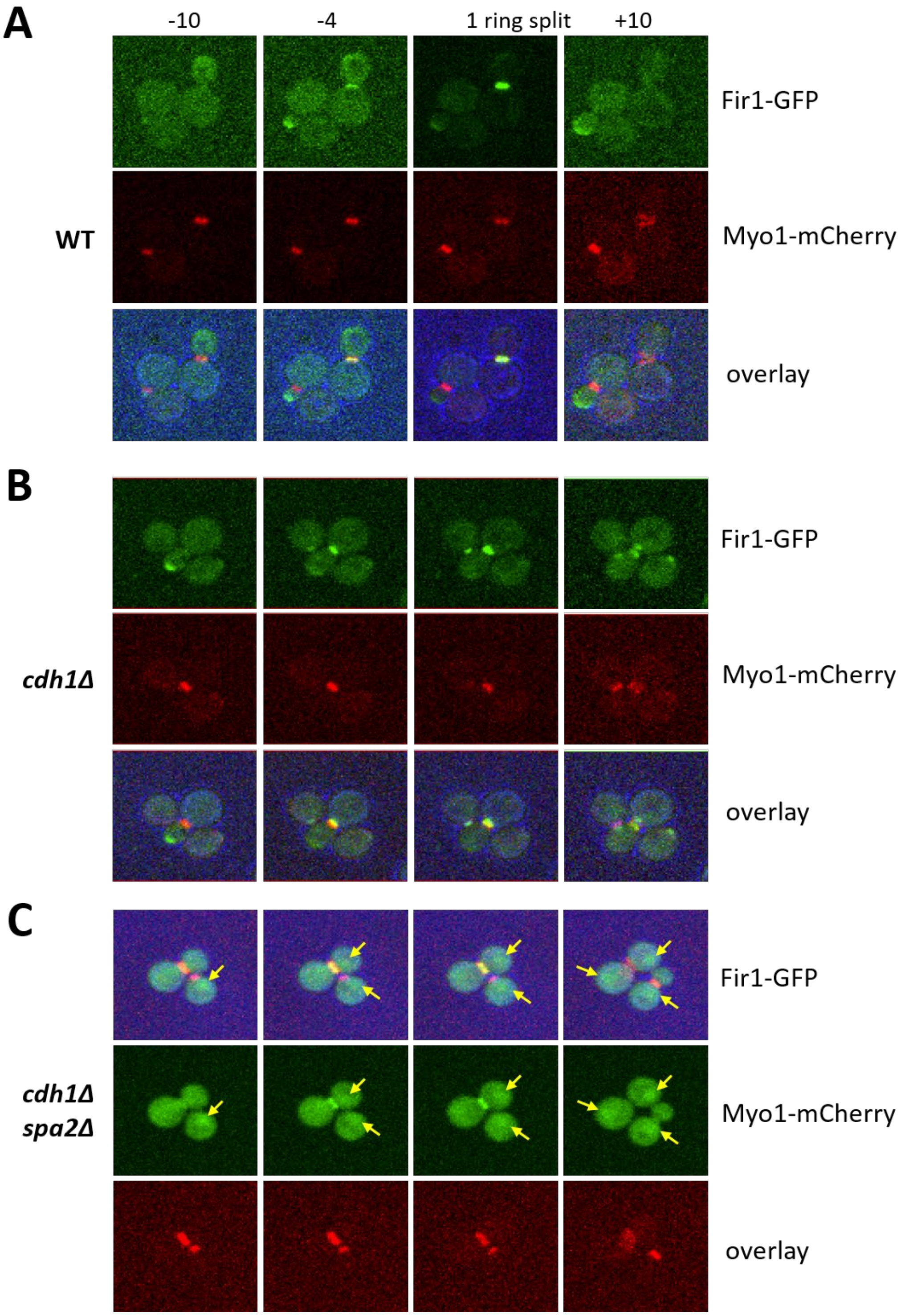

Figure S3. Fir1 relocalizes to the cell nucleus in the absence of Spa2. (**A**) Wild-type (WT), (**B**) *cdh1Δ*, or (**C**) *cdh1Δ spa2Δ* yeast cells expressing Fir1-GFP and Myo1-mCherry were analyzed by fluorescence microscopy as described in Müller et al. 2023. Images were taken 10 min (-10) or 4 min (-4) before septin ring splitting (0), at the time of ring splitting (0), or 10 min (10) after ring splitting. Arrows point to nuclear Fir1-GFP signals.

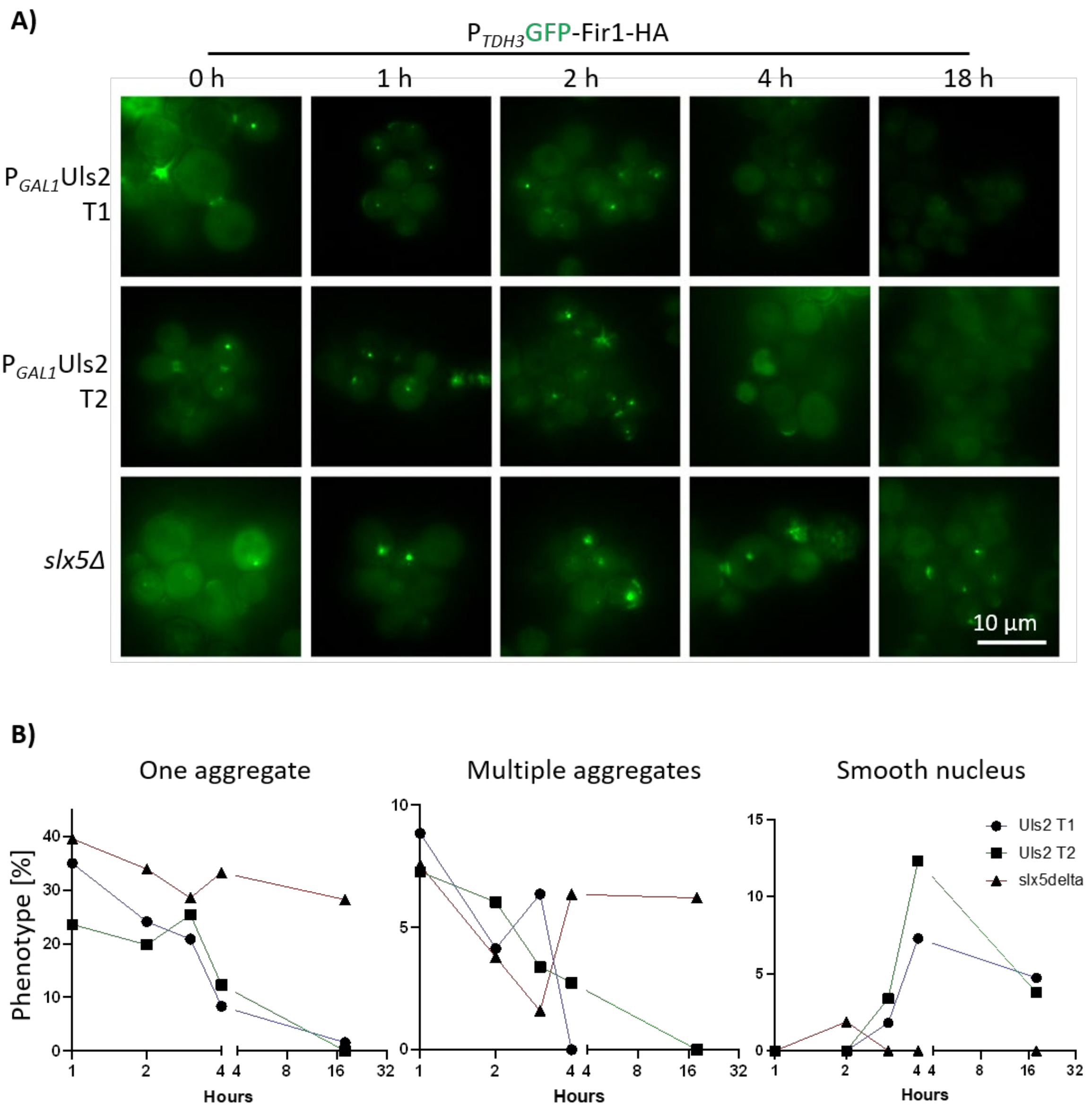

Figure S4. Fir1 levels decrease after Uls2 induction. **(A)** Overexpressed Fir1 forms aggregates in cells without Uls2 (*slx5Δ* and P_{GAL1}Uls2 in raffinose medium, timepoint 0 h). A shift to galactose medium leads to a decrease in the number of aggregates over time in the P_{GAL1}Uls2 background, whereas the number of aggregates remains stable in *slx5Δ.* A cassette expressing Fir1 N-terminally tagged with GFP and C-terminally tagged with HA under the constitutive P_{TDH3} promoter was integrated at the *LEU2* locus in *slx5Δ* or P_{GAL1}Uls2 background. Live cells were examined using fluorescence microscopy after growth in YP raffinose (Timepoint 0) and then incubated in YP galactose medium and imaged after 1 h, 2 h, 3h, 4h and 18 h. All images were adjusted with +30% brightness using PowerPoint (Microsoft). Scale bar: 10 µM. T1/2: Transformant 1 or 2. **(B)** Quantitative analysis of the cells shown in (A). Different nuclear phenotypes were quantified: cells with one large aggregate ('one aggregate'), cells with multiple aggregates ('multiple aggregates'), and cells with an evenly stained nucleus ('smooth nucleus').

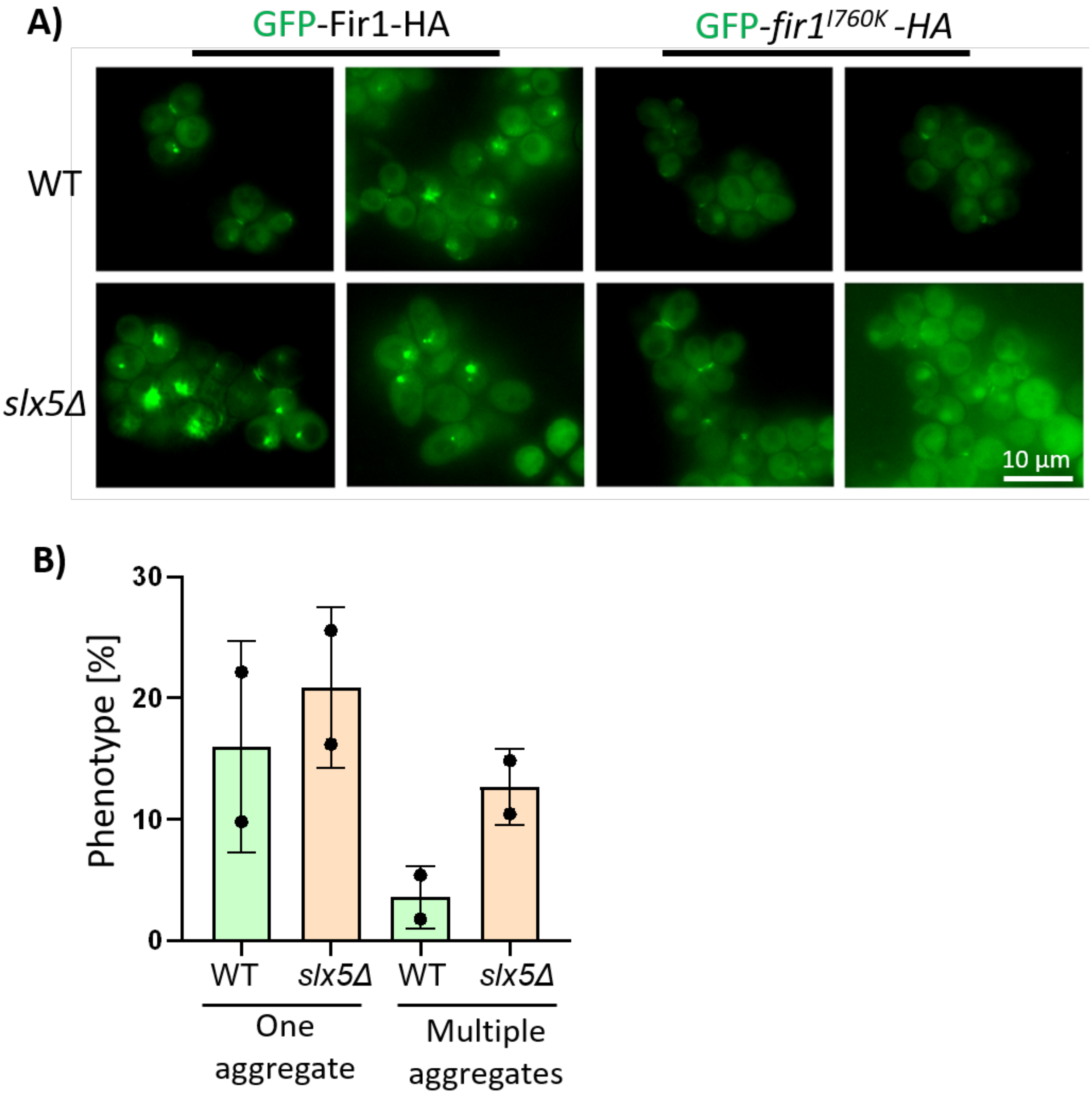

Figure S5. Fir1 aggregates accumulate in a *slx5Δ* strain. **(A)** Fir1 or Fir1-I760K N-terminally tagged with GFP and C-terminally tagged with HA were expressed from the constitutive P_{TDH3} promoter using cassettes integrated at the *LEU2* locus in wild-type (WT) or *slx5Δ* background. Live cells were examined using fluorescence microscopy after growth in YP glucose. All images were adjusted with +30% brightness and +20% contrast using PowerPoint (Microsoft). Scale bar: 10 µM. **(B)** Quantitative analysis of the cells shown in (A). (n=2)

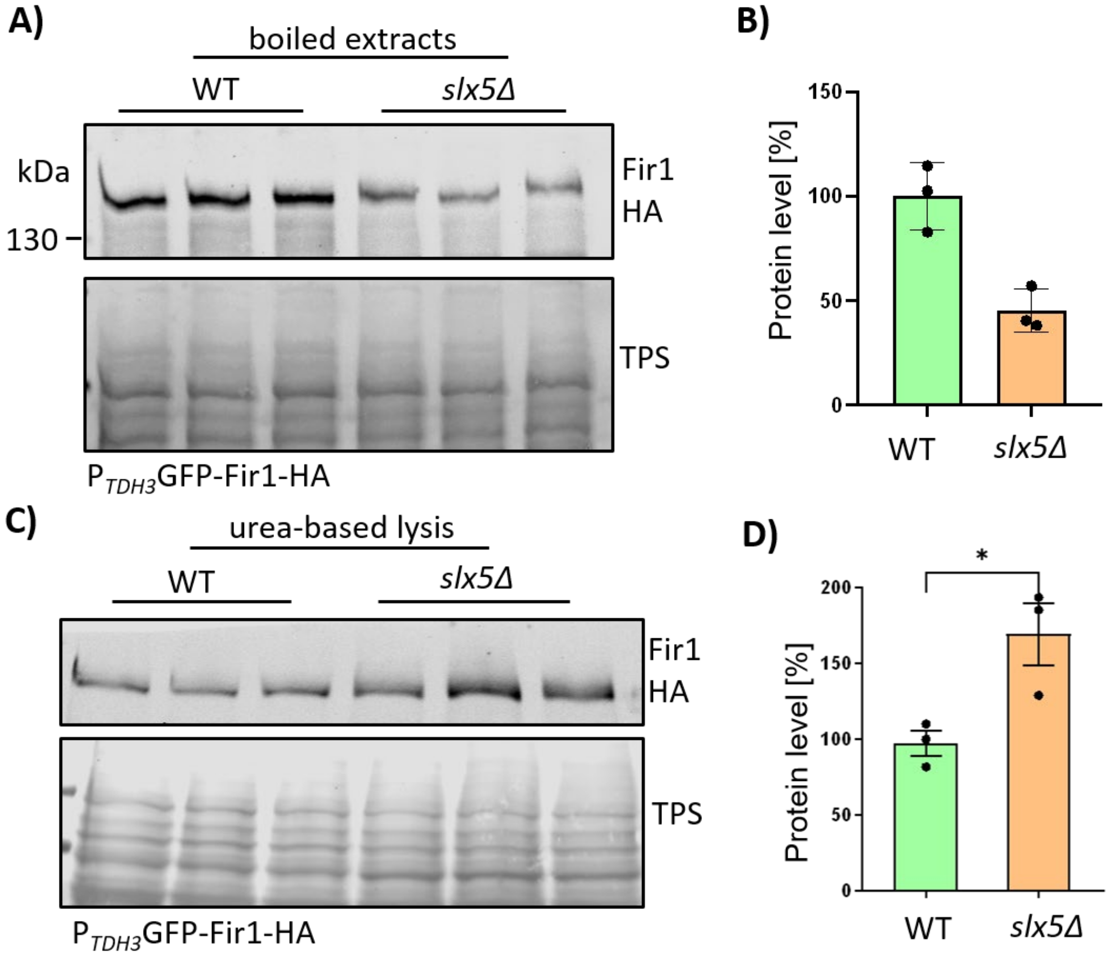

Figure S6. Levels of insoluble Fir1 are increased in a *slx5Δ* strain. **(A)** Cell lysis with Laemmli buffer does not resolve Fir1 aggregates. A cassette expressing Fir1 N-terminally tagged with GFP and C-terminally tagged with HA, expressed from the P_{TDH3} promoter, was integrated at the *LEU2* locus in wild-type (WT) or *slx5Δ* background. Cell extracts were obtained by boiling in Laemmli buffer. **B)** Quantitative analysis of the Western blot shown in (A). **C)** Increased amounts are extracted from *slx5Δ* cells using urea-based lysis. Fir1 is extracted from aggregates using a cell lysis protocol that employs 8 M urea and 4%. These cell extracts were obtained by resuspending pellets in 8 M Urea, cell disruption with glass beads, addition of 4% SDS, and subsequent heating for 5 min at 65 °C. **D)** Quantitative analysis of the Western blot shown in (A). Student's t-test: n=3; *: $p \leq 0.05$.

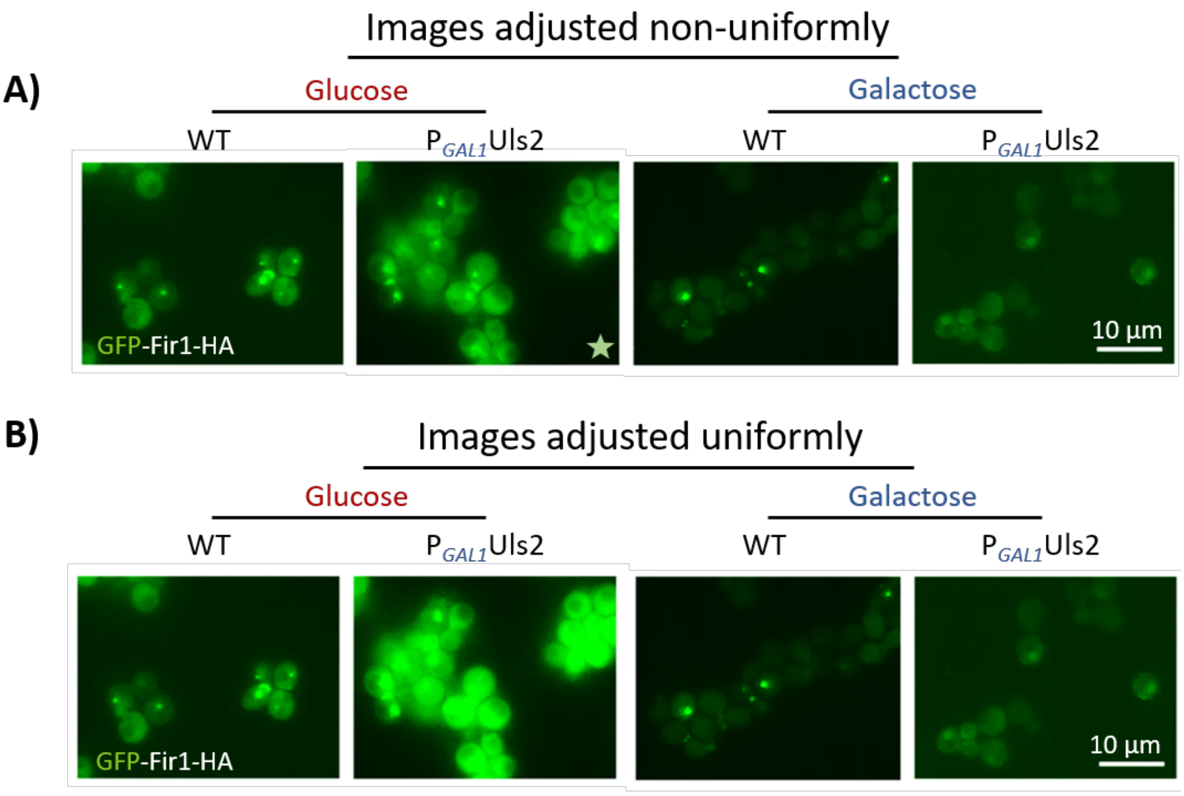

Figure S7. Comparison of microscope images from Figure 5A with images adjusted uniformly to show the much stronger GFP-Fir1 signals after repression of Uls2. **(A)** Same as Figure 5: A cassette expressing Fir1 N-terminally tagged with GFP and C-terminally tagged with HA under the constitutive P_{TDH3} promoter was integrated at the *LEU2* locus in wild-type (WT) or P_{GAL1}*SLX5* P_{GAL1}*SLX8* (P_{GAL1}*ULS2*) background. Live cells were examined using fluorescence microscopy after growth in YP medium supplemented with either 2% glucose or 2% galactose for 5 h. All images, except for P_{GAL1}*ULS2* with glucose (indicated by a green star), were adjusted with +30% brightness and +20% contrast using PowerPoint (Microsoft). **(B)** All images were adjusted with +30% brightness and +20% contrast using PowerPoint (Microsoft). Scale bar: 10 µM.

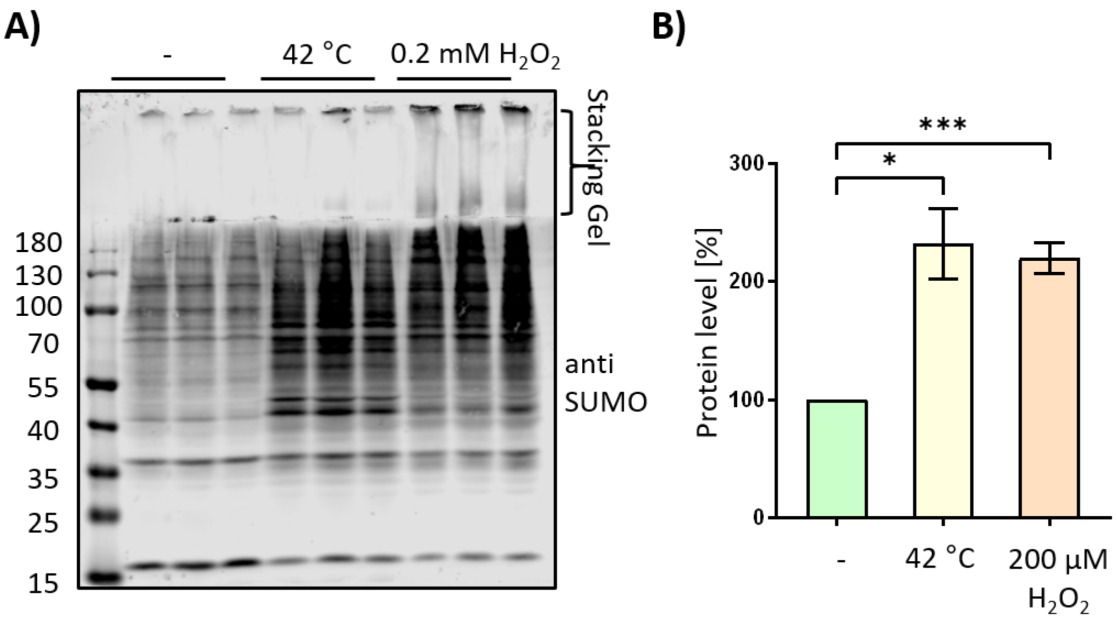

Figure S8. Oxidative stress increases high-molecular-weight SUMO conjugates. **(A)** Full Western blot of Figure 5 A comparing oxidative stress and heat stress to untreated cells. SUMOylation is increased in cells treated with H_2O_2 and grown at 42 °C. Cell lysates of wild-type (WT) cells either untreated or treated with 200 µM H_2O_2 were compared by Western blot. Cells were grown in yeast peptone (YP) medium for 4 h and, after the addition of 200 µM H_2O_2, grown for another 45 min, or without H_2O_2, grown at 42 °C. **(B)** Quantification of Blot in (A). Student's t-test: n=3; *: $p \leq 0.05$; ***:$p \leq 0.001$.

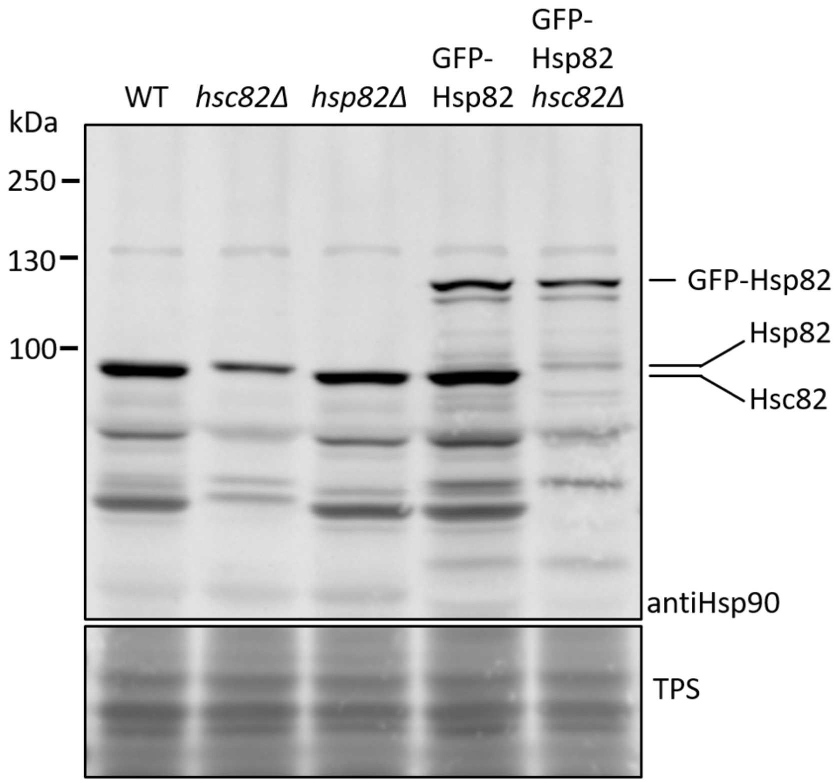

Figure S9. Verification of Hsp90 antibody specificity. The Hsp90 antibody made during this study detects Hsp82 and Hsc82. Hsp90 antibody was tested uspUC-derived cloning vectors with differenting different strains either lacking Hsc82 (*hsc82Δ*) or Hsp82 (*hsp82Δ*). Additionally, Hsp82 was put under the control of the *TDH3* promoter and N-terminally tagged with GFP using the plasmid pYM-N17 [41].

[41] Janke, C.; Magiera, M.M.; Rathfelder, N.; Taxis, C.; Reber, S.; Maekawa, H.; Moreno-Borchart, A.; Doenges, G.; Schwob, E.; 784 Schiebel, E.; et al. A versatile toolbox for PCR-based tagging of yeast genes: new fluorescent proteins, more markers and 785 promoter substitution cassettes. *Yeast* **2004**, *21*, 947-962.
